# Palladium-Catalyzed Selenylative Alkoxycarbonylation of Alkynes toward β‑Selenated Cinnamic Acid Esters

**DOI:** 10.1021/acs.orglett.5c02491

**Published:** 2025-07-04

**Authors:** Fengxiang Zhu, Huanan Wu, Xiao-Feng Wu

**Affiliations:** † School of Chemistry and Chemical Engineering, 12441 Shanxi University, Taiyuan 030006, China; ‡ Dalian National Laboratory for Clean Energy, Dalian Institute of Chemical Physics, Chinese Academy of Sciences, Dalian 116023, China; § 28392 Leibniz-Institut für Katalyse e.V., Rostock 18059, Germany

## Abstract

Herein, we report a palladium-catalyzed alkoxycarbonylation strategy enabling the direct synthesis of trisubstituted selenated cinnamate esters from commercially available starting materials. This method addresses the limitations of conventional approaches in constructing sterically congested trisubstituted derivatives by achieving efficient regio- and stereoselective installation of vinyl selenides in a single catalytic operation. The protocol establishes a robust platform for synthesizing architecturally complex cinnamate frameworks with potential applications in synthetic and medicinal chemistry. Key advantages include operational simplicity, gram-scale scalability, high regiocontrol and *Z* stereocontrol, and broad functional group tolerance, while the retained C–Se bond offers versatility for downstream derivatization.

## Graphical abstract

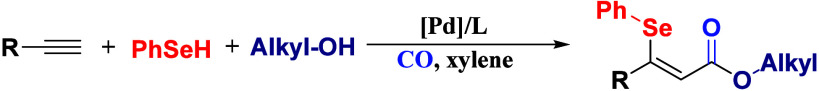

Substituted alkenes constitute fundamental structural motifs in modern chemical science, serving as critical building blocks for functional polymers, therapeutic agents, and specialty chemicals.^1–7^ Among these diverse systems, α,β-unsaturated aryl and alkyl esters (cinnamate ester derivatives), abundant in plants,^8–10^ have attracted significant attention due to their unique combination of bioactivity and synthetic versatility (Scheme 1A). These compounds exhibit broad pharmaceutical and pesticide activities, including anticancer,^11^ antioxidant,^12^ antimicrobial,^13^ insecticidal,^14^ and herbicidal effects,^15^ with favorable pharmacokinetic profiles enhancing their therapeutic potential.^16^ Beyond pharmaceutical and pesticide applications, their structural adaptability enables widespread use in food additives, spice formulations, advanced materials, and sustainable agrochemicals,^11,13^ establishing them as privileged scaffolds in interdisciplinary research.

**Scheme 1:**
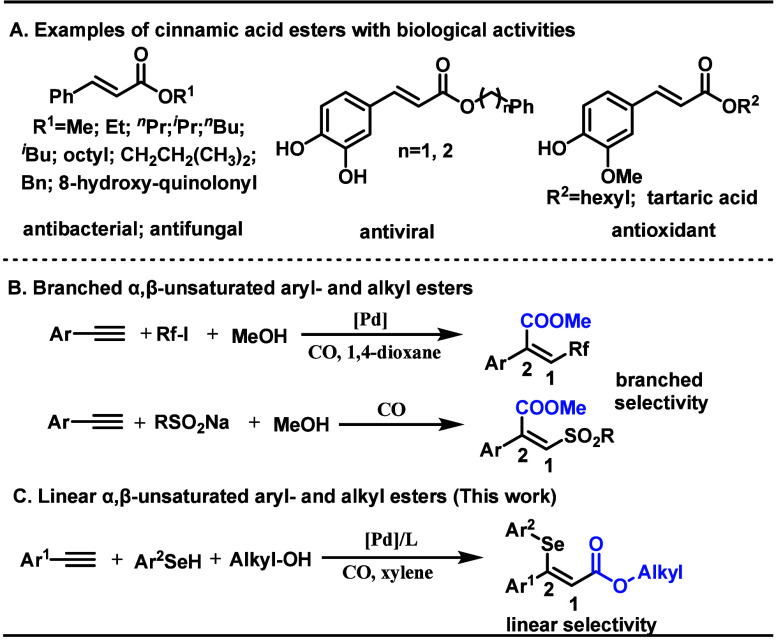
Examples of Bioactive Compounds and Reaction Design

Conventional methods for synthesizing disubstituted cinnamates such as Wittig olefination,^17,18^ Julia–Kocienski coupling,^19,20^ Peterson elimination,^21,22^ Heck reaction,^23–25^ and Horner–Wadsworth–Emmons (HWE) reactions.^26,27^ face inherent limitations when applied to constructing trisubstituted derivatives. This synthetic deficiency is particularly significant, given the growing demand for complex bioactive molecules in drug discovery. Although transition metal-catalyzed alkyne functionalization^28–32^ has recently provided partial solutions, these methods require the presynthesis of specialized alkyne ester precursors, compromising step economy and scalability (Scheme 1B). Therefore, developing efficient synthetic routes to trisubstituted cinnamate esters remains a priority.

Vinyl selenides have emerged as valuable structural motifs in medicinal chemistry due to their redox-responsive properties and metabolic stability.^33–35^ The C–Se bond’s versatility allows efficient postfunctionalization through selenoxide elimination or transition metal-catalyzed cross-couplings, offering strategic advantages for molecular diversification.^36–38^ Building on these characteristics, we developed a palladium-catalyzed alkoxycarbonylation to develop a unified platform for synthesizing selenated cinnamate esters (Scheme 1C). This approach achieves atom-economical construction of geometrically defined products through simultaneous regio- and stereocontrol in a single catalytic operation, circumventing traditional multistep sequences. Crucially, the reaction avoids free radical pathways, thereby suppressing competing formation of 2-phenyl acrylate derivatives commonly observed in alkyne difunctionalization–carbonylation processes.^39,40^

The reaction conditions for the synthesis of β-selenyl acrylate **4aaa** were systematically optimized using phenylacetylene (**1a**, 2.5 equiv), phenylselenol (**2a**, 1.0 equiv), ethanol (**3a**, 4.0 equiv), and carbon monoxide (Table 1). Initial screening with PdCl_2_ (10 mol %) and PPh_3_ (20 mol %) in 1,4-dioxane under 15 bar of CO at 120 °C for 15 h afforded **4aaa** in 88% GC yield (entry 1). Control experiments confirmed the indispensability of PdCl_2_, as its exclusion resulted in complete suppression of product formation (entry 2). Subsequent ligand screening established PPh_3_ as the optimal ligand (entries 7–19). Electron-deficient ligands such as P­(*p*-F-C_6_H_4_)_3_ (entry 18) and bidentate DPPF (entry 15) demonstrated moderate catalytic activity, whereas sterically encumbered ligands (^*t*^Bu_3_P) and flexible diphosphines (DPPBz) significantly impeded the reaction (entries 10 and 11, respectively). Evaluation of palladium precursors revealed that (PhCN)_2_PdCl_2_ delivered **4aaa** in 77% GC yield (entry 6), while Pd­(OAc)_2_, Pd­(TFA)_2_, and Pd­(acac)_2_ exhibited inferior performance (entries 3–5, respectively). Solvent studies emphasized the critical importance of 1,4-dioxane; substitutions with xylene, THF, DCM, and acetonitrile diminished yields by 13–75% (entries 20–23, respectively), and polar aprotic solvents (DMF and DMSO) resulted in negligible product formation (entries 24 and 25, respectively). Notably, employing ethanol (**3a**) as the solvent drastically reduced the efficiency (entry 26, 16% GC yield). Further optimization of the CO pressure determined the optimal condition to be 15 bar, with deviations to 10 or 20 bar leading to diminished yields (entry 27 or 28, respectively). Intriguingly, the addition of bases (Na_2_CO_3_, Et_3_N, etc.) suppressed catalytic activity (entries 29–32), indicating that external activation of the nucleophilic species is unnecessary.

**Table 1:**
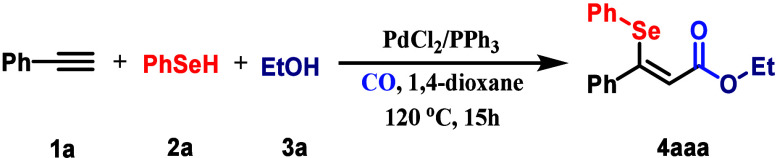
Screening of the Optimal Reaction Conditions^a^

entry	variation from the standard conditions	yield (%)
1	–	88
2	without PdCl_2_	–
3	Pd(OAc)_2_ instead of PdCl_2_	38
4	Pd(TFA)_2_ instead of PdCl_2_	41
5	Pd(acac)_2_ instead of PdCl_2_	45
6	(C_6_H_5_CN)_2_PdCl_2_ instead of PdCl_2_	77
7	1,10-Phen instead of PPh_3_	23
8	BuPAd_2_ instead of PPh_3_	54
9	Cy_3_P instead of PPh_3_	21
10	^*t*^Bu_3_P instead of PPh_3_	19
11	DPPBz instead of PPh_3_	trace
12	JohnPhos instead of PPh_3_	27
13	DPEPhos instead of PPh_3_	24
14	XantPhos instead of PPh_3_	29
15	DPPF instead of PPh_3_	55
16	DPPP instead of PPh_3_	35
17	(*p*-OMe-C_6_H_4_)_3_P instead of PPh_3_	49
18	(*p*-F-C_6_H_4_)_3_P instead of PPh_3_	71
19	(*p*-CF_3_-C_6_H_4_)_3_P instead of PPh_3_	66
20	xylene instead of 1,4-dioxane	75
21	THF instead of 1,4-dioxane	34
22	DCM instead of 1,4-dioxane	71
23	CH_3_CN instead of 1,4-dioxane	13
24	DMF instead of 1,4-dioxane	–
25	DMSO instead of 1,4-dioxane	–
26	EtOH instead of 1,4-dioxane	16
27	CO (10 bar)	45
28	CO (20 bar)	73
29	with an extra 2.0 equiv of Na_2_CO_3_	35
30	with an extra 2.0 equiv of EtONa	33
31	with an extra 2.0 equiv of ^*t*^BuOK	28
32	with an extra 2.0 equiv of Et_3_N	22

a Reaction conditions: **1a** (0.25 mmol, 2.5 equiv), **2a** (0.1 mol, 1.0 equiv), **3a** (0.4 mmol, 4.0 equiv), CO (15 bar), PdCl_2_ (10 mol %), PPh_3_ (20 mol %), 1,4-dioxane (1 mL), stirred at 120 °C for 15 h. Yields were determined by GC analysis using hexadecane as the internal standard. Abbreviations: 1,10-Phen, 1,10-phenanthroline; BuPAd_2_, butyldi-1-adamantylphosphine; DPPBz, 1,2-bis­(diphenylphosphanyl)­benzene; JohnPhos, (2-biphenyl)-di-*tert*-butylphosphine; DPEPhos, bis­(2-diphenyphosphinophenyl)­ether; XantPhos, 4,5-bis­(diphenylphosphino)-9,9-dimethylxanthene; DPPF, 1,1′-bis­(diphenylphosphino)­ferrocene; DPPP, 1,3-bis­(diphenylphosphino)­propane.

To assess the versatility of this alkoxycarbonylation platform, we first investigated various alcohols and phenylselenols (Scheme 2). Primary alcohols exhibited excellent reactivity, with methanol (**3b**), ethanol (**3a**), and *n*-butanol (**3c**) delivering β-selenyl acrylates **4aaa–4aac**, respectively, in 81–88% isolated yields. Secondary alcohols demonstrated moderate efficiency, as evidenced by isopropyl alcohol (**3d**, 71%) and *iso*-butanol (**3e**, 69%), while cyclohexanol (**3f**) retained high reactivity (87% yield), indicating that the cyclic structure did not significantly impede reactivity. Notably, tertiary alcohol **3g** remained compatible, affording **4aag** in 63% yield, despite inherent steric constraints. The methodology tolerated diverse functional groups on aliphatic chains. Ethylene glycol monoethyl ether (**3h**) containing an ethoxy group provided **4aah** in 58% yield, while halogenated alcohols bearing -F, -CF_2_H, -Br, -Cl, and -CCl_3_ substituents efficiently generated **4aai–4aam**, respectively, with products displaying potential for downstream derivatization. Additionally, but-3-en-1-ol (**3n**) delivered **4aan** in 66% yield. Substituted benzyl alcohols also performed effectively, with *p*-phenylbenzyl alcohol (**3o**) and *p*-methoxybenzyl alcohol (**3p**) affording **4aao** (50%) and **4aap** (73%), respectively. However, no desired product was detected when phenol was tested as the substrate here.^41^ Finally, we also extended the scope to some substituted phenylselenols. 4-Fluorophenylselenol (**2b**) and 4-chlorophenylselenol (**2c**) furnished compounds **4aba** and **4aca**, respectively, in 81–83% yields. In contrast, dimethyl diselenide (**2d**) displayed negligible reactivity (<5% yield) under the standard conditions perhaps due to its low reactivity toward oxidative addition with palladium.

**Scheme 2:**
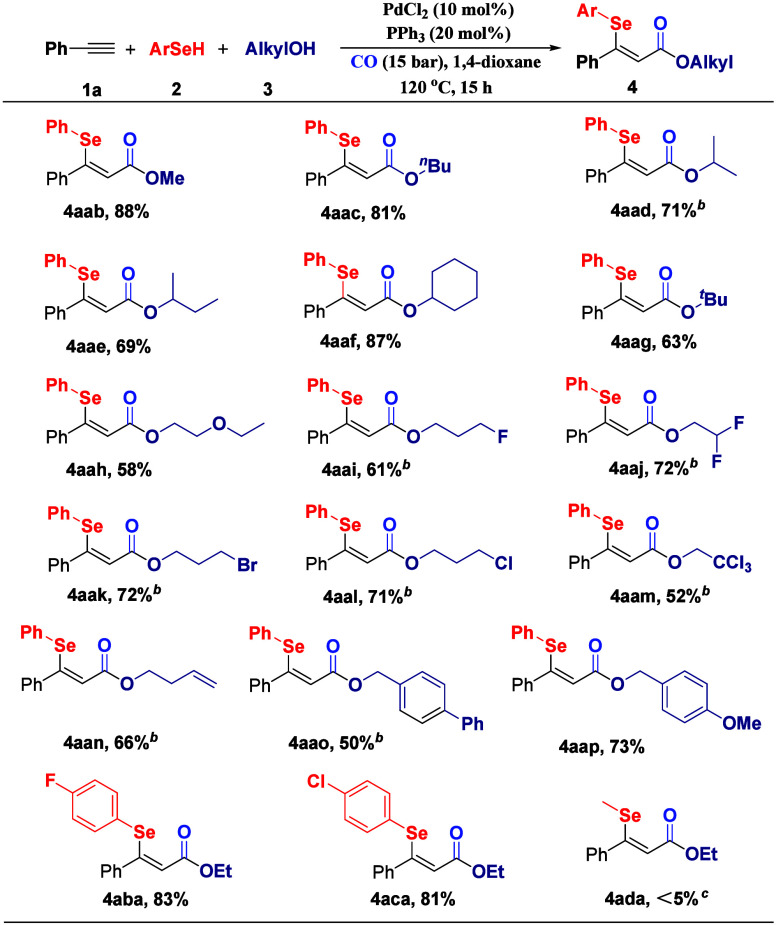
Variation of the Alcohols and Selenols^a^

a Conditions: **1a** (0.25 mmol, 2.5 equiv), **2** (0.1 mol, 1.0 equiv), **3** (0.4 mmol, 4.0 equiv), CO (15 bar), PdCl_2_ (10 mol %), PPh_3_ (20 mol %), 1,4-dioxane (1 mL), stirred at 120 °C for 15 h. Isolated yields.
b With 0.8 mmol of **2**.
c
**2**: dimethyldiselenide.

To further demonstrate the synthetic utility of this catalytic system, we investigated a diverse range of alkyne substrates (Scheme 3). Arylacetylenes with neutral substituents exhibited excellent reactivity. Methyl-substituted phenylacetylene (**1b**) afforded product **4baa** in 81% yield, whereas *p*-methoxyphenylacetylene (**1c**) was less efficient (62% yield), likely due to electronic deactivation of the alkyne by the electron-donating methoxy group. Halogenated substrates performed admirably, with *p*-fluorophenylacetylene (**1d**) and *p*-chlorophenylacetylene (**1e**) providing **4daa** and **4eaa** in 76% and 77% yields, respectively. *o*-Chlorophenylacetylene (**1f**) displayed an attenuated reactivity (**4faa**, 61%), attributed to steric hindrance. Substrates bearing electron-withdrawing groups, including -CF_3_, -OCF_3_, and -CN, were effectively incorporated into the acrylates (**4gaa–4iaa**, respectively). Acetyl-substituted (**1j**) and phenyl-substituted (**1k**) alkynes exhibited negligible electronic interference, yielding **4jaa** (74%) and **4kaa** (78%), respectively. The protocol extended successfully to extended π-systems and heterocycles. 2-Naphthylacetylene (**1l**) and 3-ethynylthiophene (**1m**) generated **4laa** (74%) and **4maa** (62%), respectively, with preserved stereoselectivity. Linear aliphatic alkynes, such as 1-phenylpropyne (**1n**) and 1-octyne (**1o**), also proved to be effective substrates with excellent *Z*/*E* selectivity.

**Scheme 3:**
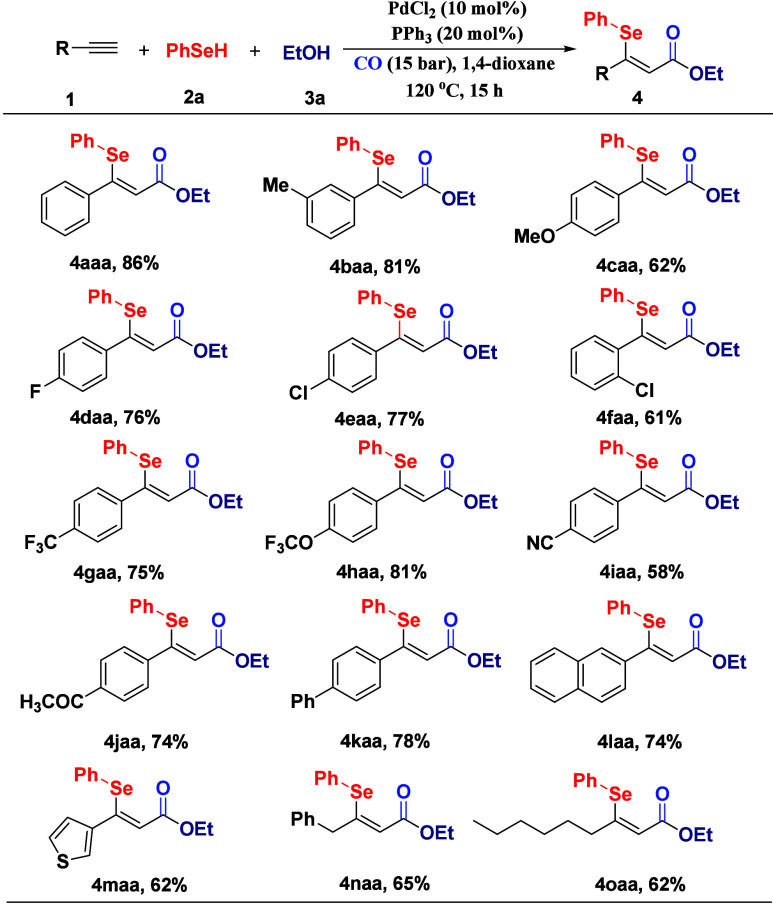
Variation of the Alkynes^a^

a Conditions: **1** (0.25 mmol, 2.5 equiv), **2a** (0.1 mol, 1.0 equiv), **3a** (0.4 mmol, 4.0 equiv), CO (15 bar), PdCl_2_ (10 mol %), PPh_3_ (20 mol %), 1,4-dioxane (1 mL), stirred at 120 °C for 15 h. Isolated yields.

To demonstrate the practical utility of this catalytic protocol, a gram-scale reaction was performed using phenylacetylene (**1a**), phenylselenol (**2a**, 3 mmol), and ethanol (**3a**) under the optimized conditions. Target β-selenyl acrylate **4aaa** was detected in 81% GC yield, confirming the scalability of this method (Scheme 4, section 1). Subsequent derivatization experiments further underscored the synthetic versatility of the products. Under the catalytic conditions of Pd­(PPh_3_)_4_ and Cu­(OAc)_2_ as the oxidant in DMF at 80 °C, treatment of **4aaa** with phenylboronic acid (1.5 equiv) resulted in substitution of the phenylselanyl group with a phenyl moiety, affording β-phenyl acrylate **5** in 81% yield (Scheme 4, section 2a). Similarly, exposure of **4aaa** to *N*-bromosuccinimide (NBS, 1.5 equiv) in dichloromethane at 60 °C delivered β-bromo acrylate **6** in 85% yield (Scheme 4, section 2b), highlighting the potential for postfunctionalization via C–Se bond cleavage.

**Scheme 4:**
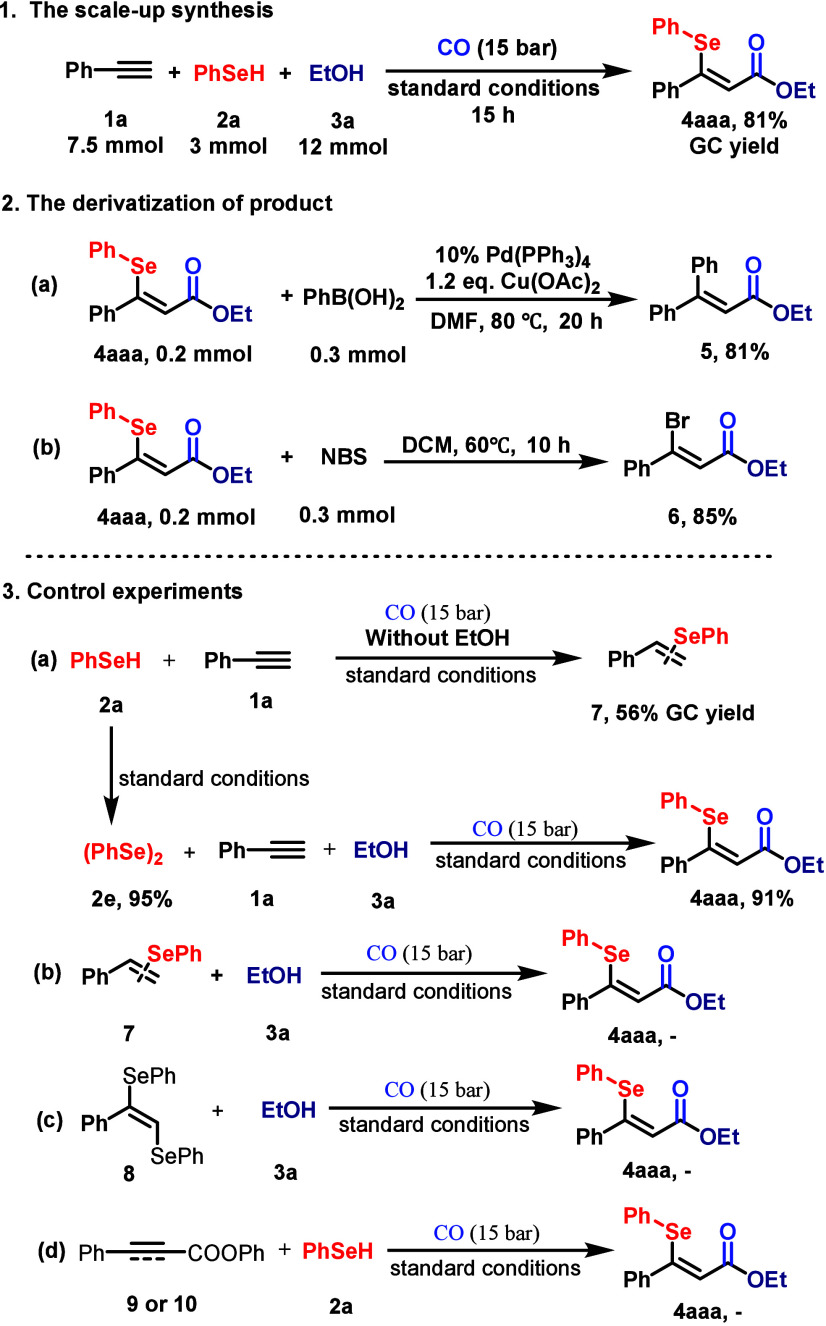
Synthetic Utility and Control Experiment

Mechanistic investigations were conducted to elucidate the reaction pathway (Scheme 4, section 3). Under the standard conditions without ethanol, phenylacetylene (**1a**) and phenylselenol (**2a**) underwent hydroselenation to afford phenyl­(styryl)­selane (**7**) as a potential intermediate.^42^ However, subjecting **7** to the catalytic system with ethanol failed to generate **4aaa**, excluding its role as an intermediate (Scheme 4, section 3b). Similarly, (*Z*)-(1-phenylethene-1,2-diyl)­bis­(phenylselane) (**8**) did not produce **4aaa** when reacted with ethanol and CO (Scheme 4, section 3c). Notably, phenylselenol (**2a**) self-coupled to form 1,2-diphenyldiselane (**2e**) in the absence of an alkyne and alcohol. This reaction may start with oxidative addition between LPd(0) and PhSeH to give PhSe-LPd-H and then X-ligand exchange with another molecule of PhSeH to give PeSe-Pd-SePh that will release PhSeSePh after reductive elimination. Crucially, **2e** reacted efficiently with phenylacetylene (**1a**), CO, and ethanol under the standard conditions, delivering **4aaa** in 91% yield (Scheme 4, section 3a), implicating **2e** as a plausible catalytic intermediate. Finally, pathways involving prealkoxycarbonylation of the alkyne followed by selenation were conclusively ruled out (Scheme 4, section 3d).

Based on the experimental results and control studies, we propose the following catalytic cycle (Scheme 5). Initially, the Pd(0) catalyst undergoes oxidative addition with 1,2-diphenyldiselane (**2e**), generated from phenylselenol (**2a** (Scheme 4, eq 3a)), to form Pd­(II)-bis­(selenide) complex **A**. Subsequently, coordination and *syn*-insertion of the alkyne (**1**) into the Pd–Se bond of complex **A** generates *Z*-configured alkenylpalladium­(II) intermediate **B**. Carbon monoxide coordinates to palladium intermediate **B**, followed by the nucleophilic attack of alcohol **3** to form palladium carbonyl intermediate **C**. Finally, intermediate **C** undergoes reductive elimination to furnish the β-selenyl acrylate product (**4**) while regenerating the Pd(0) catalyst.

**Scheme 5:**
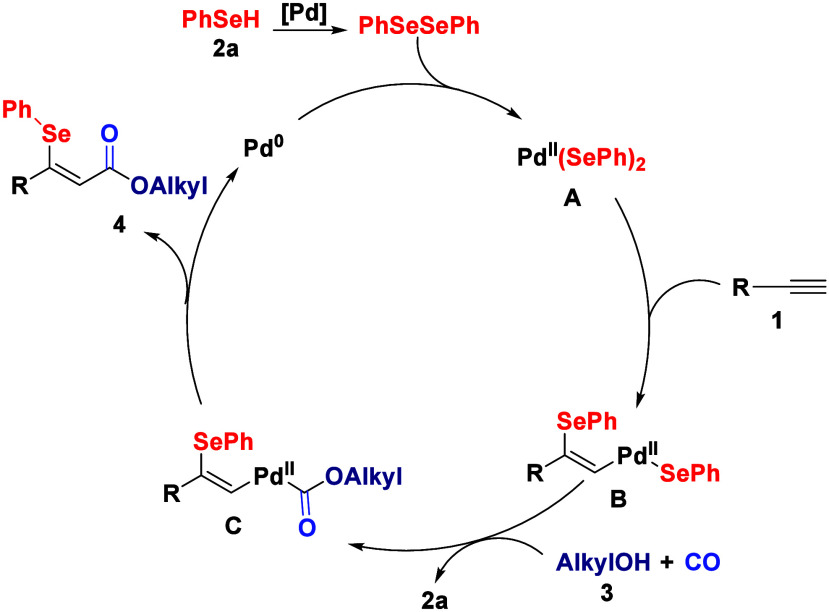
Proposed Mechanism

In conclusion, we have disclosed a palladium-catalyzed alkoxycarbonylation strategy that provides efficient access to trisubstituted selenocinnamate esters with high regiocontrol and *Z* stereocontrol. By addressing the limitations of traditional approaches, this method enables the concise synthesis of vinyl selenides from commercially available precursors. The protocol demonstrates operational simplicity, gram-scale scalability, and excellent functional group tolerance, while the preserved C–Se bond offers a versatile handle for downstream derivatization. These advances not only broaden the scope of synthetic methodologies in organoselenium chemistry but also establish a platform for designing functionalized cinnamate derivatives with potential applications.

## Supplementary Material



## Data Availability

The data underlying this study are available in the published article and its Supporting Information.

## Notes

Experimental details, characterization data, and NMR spectra (PDF)
