# Supplementary material for: Palladium-Catalyzed Selenylative Alkoxycarbonylation of Alkynes toward β‑Selenated Cinnamic Acid Esters
Source: Org Lett. 2025 Jul 4;27(28):7714–8. doi: 10.1021/acs.orglett.5c02491 (PMC12281563; doi:10.1021/acs.orglett.5c02491)

Electronic Supplementary Information For

Palladium-Catalyzed Selenylative Alkoxycarbonylation of Alkynes toward β-Selenated Cinnamic Acid Esters

Fengxiang Zhu,*[a] Huanan Wu [a] and Xiao-Feng Wu*[b]

[a] School of Chemistry and Chemical Engineering, Shanxi University, Taiyuan 030006 (China).

[b] Institution Dalian National Laboratory for Clean Energy, Dalian Institute of Chemical Physics, Chinese Academy of Sciences, Dalian 116023 (China); Leibniz-Institut für Katalyse e.V., Rostock 18059 (Germany)

E-mail: zfx201989@sxu.edu.cn (Fengxiang Zhu); xwu2020@dicp.ac.cn; xiao-feng.wu@catalysis.de (Xiao-Feng Wu).

Table of Content

1. General Methods S2

2. Typical reaction procedure for the synthesis of β-selenyl acrylates S2

3. Characterization data for products S2-S9

4. NMR Spectrum Copies S10-S47

1. General Methods

NMR spectra were recorded on Bruker Avance NEO 600 M and 400 M. Chemical shifts (ppm) are given relative to solvent: references for $CDCl_3$ were 7.26 ppm (^{1}H-NMR) and 77.0 ppm (^{13}C-NMR). ^{13}C NMR spectra were acquired on a broad band decoupled mode. Multiplets were assigned as s (singlet), d (doublet), t (triplet), dd (doublet of doublet), m (multiplet) and br. s (broad singlet). All measurements were carried out at room temperature unless otherwise stated. Gas chromatography analysis was performed on a Shimadzu 2014 instrument with an FID detector and HP-5 capillary column (polydimethylsiloxane with 5% phenyl groups, 30 m, 0.32 mm i.d., 0.25 μm film thickness) using N_2 as carrier gas. HRMS was obtained on a Bruker Daltonics Bio-TOF-Q mass spectrometer by the ESI method. The products were isolated from the reaction mixture by column chromatography on silica gel 60, 0.063-0.2 mm, 70-230 mesh. All reactions were carried out under air atmosphere. All the reagents were purchased from Heowns, Rhawn, and Laajoo chemical company.

2. Typical reaction procedure for the synthesis of:

General procedure: A 4 mL screw-cap vial was charged with $PdCl_2$ (10 mol%), PPh_3 (20 mol%), phenyl acetylene (0.25 mmol), PhSeH (0.1 mmol), ethanol (0.4 mmol), 1,4-dioxane (1 mL) and a stirring bar. The vial was closed by a Teflon septum and a phenolic cap and connected to the atmosphere through a needle. Then the vial was fixed in an alloy plate and put into Paar 4560 series autoclave (300 mL). At room temperature, the autoclave is flushed with CO for three times and 15 bar of CO was charged. The autoclave was placed on a heating plate equipped with magnetic stirring and an aluminum block. The reaction was heated at 120 °C for 15 hours. Afterwards, the autoclave was cooled to room temperature and the pressure carefully released. Upon completion, the reaction mixture was concentrated under vacuum. The residue was purified by silica gel column chromatography using a petroleum ether/AcOEt (200:1) as the eluent to give the corresponding products.

Large scale procedure: A 20 mL screw-cap vial was charged with $PdCl_2$ (10 mol%), PPh_3 (20 mol%), phenyl acetylene (7.5 mmol), PhSeH (30 mmol), ethanol (12 mmol), 1,4-dioxane (10 mL) and a stirring bar. The vial was closed by a Teflon septum and a phenolic cap and connected to the atmosphere through a needle. Then the vial was fixed in an alloy plate and put into Paar 4560 series autoclave (300 mL). At room temperature, the autoclave is flushed with CO for three times and 15 bar of CO was charged. The autoclave was placed on a heating plate equipped with magnetic stirring and an aluminum block. The reaction was heated at 120 °C for 15 hours. Afterwards, the autoclave was cooled to room temperature and the pressure carefully released. Upon completion, the reaction mixture was concentrated under vacuum. The residue was purified by silica gel column chromatography using a petroleum ether/AcOEt (200:1) as the eluent to give the corresponding product **4aaa** in 80% yield (796.8 mg).

3. Characterization data for products

ethyl (Z)-3-phenyl-3-(phenylselanyl)acrylate

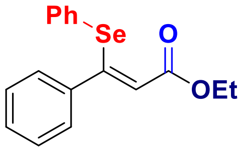

4aaa

Purification by flash column chromatography (petroleum ether/EtOAc = 200:1). Yellow oily (28.6 mg, 86%). ^{1}H NMR (600 MHz, Chloroform-d) δ 7.28 (d, J = 1.7 Hz, 1H), 7.25 (d, J = 7.7 Hz, 2H), 7.10 (t, J = 6.9 Hz, 2H), 7.07 – 7.06 (m, 1H), 7.06 – 7.02 (m, 3H), 7.01 (d, J = 6.7 Hz, 1H), 6.34 (s, 1H), 4.32 (q, J = 7.1 Hz, 2H), 1.37 (t, J = 7.1 Hz, 3H). ^{13}C NMR (151 MHz, Chloroform-*d*) δ 166.8, 161.3, 139.1, 136.3, 129.2, 128.6, 128.4, 128.0, 127.9, 127.5, 117.0, 60.5, 14.4. GC-MS(EI-70eV): m/z (%) 332(43), 286(15), 259(29), 175(25), 103(100). HRMS (ESI): calcd for $C_{17}H_{17}O_2Se^+$ [M+H]$^+$: 333.0388, found: 333.0391.

methyl (Z)-3-phenyl-3-(phenylselanyl)acrylate

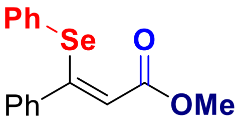

4aab

Purification by flash column chromatography (petroleum ether/EtOAc = 200:1). Yellow oily (28.0mg, 88%). ^{1}H NMR (500 MHz, Chloroform-*d*) δ 7.25 (d, *J* = 7.0 Hz, 2H), 7.14 – 7.08 (m, 1H), 7.08 – 7.05 (m, 2H), 7.05 – 7.02 (m, 4H), 7.00 (d, *J* = 7.4 Hz, 1H), 6.36 (s, 1H), 3.85 (s, 3H). ^{13}C NMR (126 MHz, Chloroform-*d*) δ 167.1, 161.7, 139.1, 136.3, 129.1, 128.6, 128.4, 128.0, 127.9, 127.5, 116.5, 51.6. GC-MS(EI-70eV): m/z (%) 318 (33), 286 (15), 259 (18), 161 (100), 157(32). HRMS (ESI): calcd for $C_{16}H_{15}O_2Se^+$ [M+H]$^+$: 319.0232, found: 319.0229.

butyl (Z)-3-phenyl-3-(phenylselanyl)acrylate

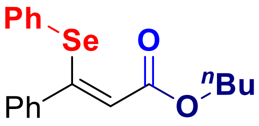

4aac

Purification by flash column chromatography (petroleum ether/EtOAc = 200:1). Yellow oily (29.2 mg, 81%). ^{1}H NMR (500 MHz, Chloroform-*d*) δ 7.23 (d, *J* = 7.0 Hz, 2H), 7.08 (t, *J* = 7.4 Hz, 1H), 7.06 – 7.01 (m, 4H), 7.01 (d, *J* = 3.7 Hz, 1H), 7.00 - 6.95 (m, 2H), 6.33 (s, 1H), 4.24 (t, *J* = 6.7 Hz, 2H), 1.75 – 1.67 (m, 2H), 1.50 – 1.41 (m, 2H), 0.96 (t, *J* = 7.4 Hz, 3H). ^{13}C NMR (126 MHz, Chloroform-*d*) δ 166.8, 161.2, 139.1, 136.3, 129.2, 128.6, 128.4, 127.9, 127.9, 127.5, 117.1, 64.5, 30.8, 19.2, 13.7. GC-MS(EI-70eV): m/z (%) 360 (53), 287 (15), 259 (32), 157 (19), 147 (100). HRMS (ESI): calcd for $C_{19}H_{21}O_2Se^+$ [M+H]$^+$: 361.0701, found: 361.0703.

isopropyl (Z)-3-phenyl-3-(phenylselanyl)acrylate

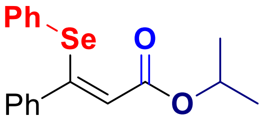

4aad

Purification by flash column chromatography (petroleum ether/EtOAc = 200:1). Yellow oily (24.6 mg, 71%). ^{1}H NMR (500 MHz, Chloroform-*d*) δ 7.25 (d, *J* = 7.4 Hz, 2H), 7.13 – 7.08 (m, 1H), 7.05 – 7.01 (m, 5.7 Hz, 5H), 7.00 (d, *J* = 7.5 Hz, 2H), 6.32 (s, 1H), 5.24 – 5.17 (m, 1H), 1.36 (d, *J* = 6.2 Hz, 6H). ^{13}C NMR (126 MHz, Chloroform-*d*) δ 166.3, 160.8, 139.2, 136.3, 129.3, 128.6, 128.3, 127.9, 127.8, 127.4, 117.5, 68.0, 22.1. GC-MS(EI-70eV): m/z (%) 346 (69), 287 (19), 259 (37), 157 (27), 147 (100). HRMS (ESI): calcd for $C_{18}H_{19}O_2Se^+$ [M+H]$^+$: 347.0545, found: 347.0541.

Sec-butyl (Z)-3-phenyl-3-(phenylselanyl)acrylate

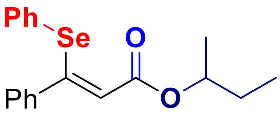

4aae

Purification by flash column chromatography (petroleum ether/EtOAc = 200:1). Light yellow solid (24.8 mg, 69%). ^{1}H NMR (400 MHz, Chloroform-*d*) δ 7.37 - 7.31 (m 1H), 7.16 (d, *J* = 7.1 Hz, 2H), 7.01 (t, *J* = 7.4 Hz, 1H), 6.98 – 6.92 (m, 5H), 6.91 (d, *J* = 7.3 Hz, 1H), 6.24 (s, 1H), 4.98 – 4.90 (m, 1H), 1.66 – 1.55 (m, 2H), 1.24 (d, *J* = 6.3 Hz, 3H), 0.89 (t, *J* = 7.4 Hz, 3H). ^{13}C NMR (151 MHz, Chloroform-*d*) δ 166.5, 160.8, 139.2, 136.3, 129.3, 128.6, 128.3, 127.9, 127.8, 127.4, 117.5, 72.5, 29.0, 19.6, 9.7. GC-MS(EI-70eV): m/z (%) 360 (49), 287 (20), 259 (24), 157 (22), 57(100). HRMS (ESI): calcd for $C_{19}H_{21}O_2Se^+$ [M+H]$^+$: 361.0701, found: 361.0699.

cyclohexyl (Z)-3-phenyl-3-(phenylselanyl)acrylate

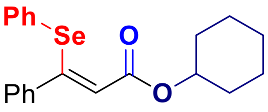

4aaf

Purification by flash column chromatography (petroleum ether/EtOAc = 200:1). Yellow oily (33.6 mg, 87%). ^{1}H NMR (400 MHz, Chloroform-*d*) δ 7.42 (t, *J* = 6.3 Hz, 1H), 7.23 (d, *J* = 7.3 Hz, 2H), 7.08 (t, *J* = 7.4 Hz, 1H), 7.03 – 7.00 (m, 5H), 6.97 (d, *J* = 7.4 Hz, 1H), 6.31 (s, 1H), 4.96 – 4.92 (m, 1H), 1.83 – 1.71 (m, 2H), 1.59 – 1.50 (m, 3H), 1.49 – 1.35 (m, 3H), 1.34 – 1.21 (m, 2H). ^{13}C NMR (101 MHz, Chloroform-*d*) δ 166.3, 160.7, 139.2, 136.3, 129.4, 128.6, 128.3, 127.9, 127.8, 127.4, 117.6, 73.0, 31.8, 25.5, 23.8. GC-MS(EI-70eV): m/z (%) 386 (51), 304 (33), 287 (16), 259 (29), 210 (100). HRMS (ESI): calcd for $C_{21}H_{23}O_2Se^+$ $[M+H]^+$: 387.0858, found: 387.0861.

***tert*-butyl (Z)-3-phenyl-3-(phenylselanyl)acrylate**

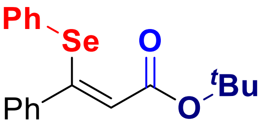

4aag

Purification by flash column chromatography (petroleum ether/EtOAc = 200:1). Yellow oily (22.7 mg, 63%). ^{1}H NMR (500 MHz, Chloroform-*d*) δ 7.22 (d, *J* = 7.1 Hz, 2H), 7.07 (t, *J* = 7.4 Hz, 1H), 7.04 – 6.99 (m, 5H), 6.97 (d, *J* = 7.4 Hz, 2H), 6.25 (s, 1H), 1.57 (s, 9H). ^{13}C NMR (126 MHz, Chloroform-*d*) δ 166.2, 159.3, 139.2, 136.3, 129.4, 128.7, 128.3, 127.8, 127.4, 118.9, 80.9, 28.4. GC-MS(EI-70eV): m/z (%) 360 (30), 287 (22), 259 (35), 157 (22), 102 (29). HRMS (ESI): calcd for $C_{19}H_{21}O_2Se^+$ $[M+Na]^+$: 361.0701, found: 361.0701.

2-ethoxyethyl (Z)-3-phenyl-3-(phenylselanyl)acrylate

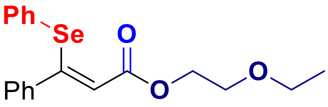

4aah

Purification by flash column chromatography (petroleum ether/EtOAc = 120:1). Yellow oily (21.8 mg, 58%). ^{1}H NMR (500 MHz, Chloroform-*d*) δ 7.22 (d, *J* = 7.0 Hz, 2H), 7.11 – 7.06 (m, 1H), 7.06 – 7.03 (m, 1H), 7.03 – 7.00 (m, 4H), 6.98 (d, *J* = 7.4 Hz, 2H), 6.39 (s, 1H), 4.44 – 4.34 (m, 2H), 3.77 – 3.68 (m, 2H), 3.57 (q, *J* = 7.0 Hz, 2H), 1.24 (t, *J* = 7.0 Hz, 3H). ^{13}C NMR (126 MHz, Chloroform-*d*) δ 166.6, 161.9, 139.1, 136.3, 129.2, 128.6, 128.4, 128.0, 127.9, 127.5, 116.8, 68.5, 66.7, 63.8, 15.1. GC-MS(EI-70eV): m/z (%) 377 (17), 287 (37), 258 (55), 157 (32), 129 (100). HRMS (ESI): calcd for $C_{19}H_{20}NaO_3Se^+$ $[M+Na]^+$: 399.0470, found: 399.0468.

3-fluoropropyl (Z)-3-phenyl-3-(phenylselanyl)acrylate

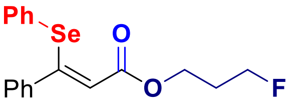

4aai

Purification by flash column chromatography (petroleum ether/EtOAc = 200:1). Yellow oily (22.2 mg, 61%). ^{1}H NMR (500 MHz, Chloroform-*d*) ^{1}H NMR (500 MHz, Chloroform-*d*) δ 7.25 (d, *J* = 7.3 Hz, 2H), 7.15 – 7.09 (m, 1H), 7.09 – 7.06 (m, 1H), 7.06 – 7.02 (m, 4H), 7.01 (d, *J* = 7.5 Hz, 2H), 6.35 (s, 1H), 4.66 (t, *J* = 5.8 Hz, 1H), 4.57 (t, *J* = 5.8 Hz, 1H), 4.40 (t, *J* = 6.2 Hz, 2H), 2.15 (dp, *J* = 24.6, 6.0 Hz, 2H). ^{13}C NMR (126 MHz, Chloroform-*d*) δ 166.5, 162.1, 139.1, 136.3, 129.1, 128.6, 128.4, 128.0, 127.9, 127.5, 116.6, 80.7 (d, *J* = 82.5 Hz), 60.4 (d, *J* = 3.2 Hz), 29.9 (d, *J* = 10.1 Hz). ^{19}F NMR (471 MHz, Chloroform-*d*) δ -222.13. GC-MS(EI-70eV): m/z (%) 364 (45), 286 (16), 259 (27), 157 (21), 147 (100). HRMS (ESI): calcd for $C_{18}H_{17}NaFO_2Se^+$ $[M+Na]^+$: 387.0270, found: 387.0266.

2,2-difluoroethyl (Z)-3-phenyl-3-(phenylselanyl)acrylate

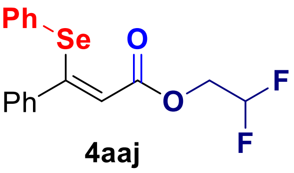

4aaj

Purification by flash column chromatography (petroleum ether/EtOAc = 200:1). Yellow oily (26.5 mg, 72%). ^{1}H NMR (500 MHz, Chloroform-*d*) δ 7.23 (d, *J* = 7.2 Hz, 2H), 7.16 – 7.08 (m, 2H), 7.06 (d, *J* = 7.9 Hz, 1H), 7.05 – 6.97 (m, 5H), 6.38 (s, 1H), 6.04 (tt, *J* = 55.2, 4.1 Hz, 1H), 4.43 (td, *J* = 13.6, 4.1 Hz, 2H). ^{13}C NMR (126 MHz, Chloroform-*d*) δ 165.4, 164.7, 138.9, 136.3, 128.8, 128.5, 128.5, 128.3, 128.1, 127.6, 115.2, 112.9 (t, *J* = 241.9 Hz), 62.5 (t, *J* = 29.0 Hz). ^{19}F NMR (471 MHz, Chloroform-*d*) δ -

125.26, -125.38. GC-MS(EI-70eV): m/z (%) 368 (46), 286 (15), 259 (22), 211 (100), 157 (13). HRMS (ESI): calcd for $C_{17}H_{14}NaF_2O_2Se^+$ $[M+Na]^+$: 391.0019, found: 3910023.

3-bromopropyl (Z)-3-phenyl-3-(phenylselanyl)acrylate

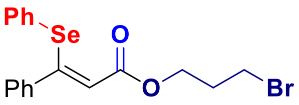

4aak

Purification by flash column chromatography (petroleum ether/EtOAc = 200:1). White solid(30.5 mg, 72%). ^{1}H NMR (400 MHz, Chloroform-*d*) δ 7.25 – 7.20 (m, 2H), 7.09 (t, *J* = 7.4 Hz, 1H), 7.05 (d, *J* = 5.0 Hz, 1H), 7.04 (d, *J* = 3.7 Hz, 2H), 7.02 – 7.01 (m, 2H), 6.99 (t, *J* = 6.5 Hz, 2H), 6.33 (s, 1H), 4.38 (t, *J* = 6.0 Hz, 2H), 3.52 (t, *J* = 6.6 Hz, 2H), 2.27 (p, *J* = 6.3 Hz, 2H). ^{13}C NMR (151 MHz, Chloroform-*d*) δ 166.4, 162.3, 139.0, 136.3, 129.1, 128.6, 128.4, 128.1, 128.0, 127.5, 116.5, 62.3, 31.9, 29.5. GC-MS(EI-70eV): m/z (%) 424 (39), 286 (25), 259 (38), 157 (28), 147 (100). HRMS (ESI): calcd for $C_{18}H_{17}NaBrO_2Se^+$ $[M+Na]^+$: 446.9469, found: 446.9473.

3-chloropropyl (Z)-3-phenyl-3-(phenylselanyl)acrylate

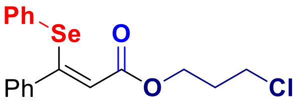

4aal

Purification by flash column chromatography (petroleum ether/EtOAc = 200:1). Yellow oily (27.0 mg, 71%). ^{1}H NMR (500 MHz, Chloroform-*d*) δ 7.23 (d, *J* = 7.1 Hz, 2H), 7.09 (t, *J* = 7.3 Hz, 2H), 7.06 – 7.03 (m, 2H), 7.03 – 7.01 (m, 2H), 6.99 (d, *J* = 8.0 Hz, 2H), 6.32 (s, 1H), 4.40 (t, *J* = 6.0 Hz, 2H), 3.67 (t, *J* = 6.4 Hz, 2H), 2.19 (p, *J* = 6.1 Hz, 2H). ^{13}C NMR (126 MHz, Chloroform-*d*) δ 166.5, 162.2, 139.0, 136.3, 129.1, 128.6, 128.4, 128.1, 128.0, 127.5, 116.5, 61.2, 41.3, 31.8. GC-MS(EI-70eV): m/z (%) 380 (40), 286 (19), 259 (33), 157 (25), 147 (100). HRMS (ESI): calcd for $C_{18}H_{17}NaClO_2Se^+$ $[M+Na]^+$: 402.9975, found: 402.9971.

2,2,2-trichloroethyl (Z)-3-phenyl-3-(phenylselanyl)acrylate

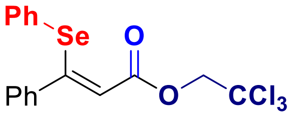

4aam

Purification by flash column chromatography (petroleum ether/EtOAc = 200:1). Yellow oily (22.6 mg, 52%). ^{1}H NMR (500 MHz, Chloroform-*d*) δ 7.24 (d, *J* = 7.1 Hz, 2H), 7.13 – 7.08 (m, 1H), 7.07 (d, *J* = 6.8 Hz, 1H), 7.06 – 7.05 (m, 2H), 7.04 – 7.03 (m, 2H), 7.03 – 6.98 (m, 2H), 6.46 (s, 1H), 4.89 (s, 2H). ^{13}C NMR (126 MHz, Chloroform-*d*) δ 165.6, 164.6, 138.9, 136.3, 128.8, 128.5, 128.5, 128.3, 128.1, 127.6, 115.1, 95.1, 74.2. GC-MS(EI-70eV): m/z (%) 434 (41), 287(28), 259 (40), 157 (28), 147 (100). HRMS (ESI): calcd for $C_{17}H_{13}NaCl_3O_2Se^+$ $[M+Na]^+$: 456.9039, found: 456.9044.

But-3-en-1-yl (Z)-3-phenyl-3-(phenylselanyl)acrylate

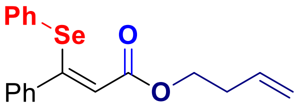

4aan

Purification by flash column chromatography (petroleum ether/EtOAc = 200:1). Yellow oily (23.6 mg, 66%). ^{1}H NMR (500 MHz, Chloroform-*d*) δ 7.23 (d, *J* = 7.8 Hz, 2H), 7.14 – 7.06 (m, 2H), 7.06 – 7.00 (m, 5H), 6.98 (d, *J* = 7.4 Hz, 1H), 6.33 (s, 1H), 5.91 – 5.76 (m, 1H), 5.16 (d, *J* = 17.2 Hz, 1H), 5.10 (d, *J* = 10.2 Hz, 1H), 4.30 (t, *J* = 6.8 Hz, 2H), 2.48 (q, *J* = 6.1, 5.6 Hz, 2H). ^{13}C NMR (126 MHz, Chloroform-*d*) δ 166.6, 161.6, 139.1, 136.3, 134.1, 129.2, 128.6, 128.4, 128.0, 127.9, 127.5, 117.3, 116.9, 63.7, 33.2. GC-MS(EI-70eV): m/z (%) 358 (22), 287 (9), 259 (14), 102 (17), 55 (100). HRMS (ESI): calcd for $C_{19}H_{19}O_2Se^+$ $[M+H]^+$: 359.0545, found: 359.0545.

[1,1’-biphenyl]-4-ylmethyl (Z)-3-phenyl-3-(phenylselanyl)acrylate

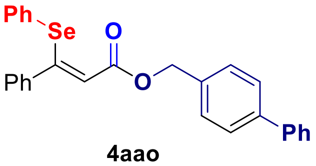

4aao

Purification by flash column chromatography (petroleum ether/EtOAc = 150:1). Yellow oily (23.5 mg, 50%). ^{1}H NMR (500 MHz, Chloroform-*d*) δ 7.63 (t, *J* = 7.4 Hz, 4H), 7.54 (d, *J* = 7.1 Hz, 2H), 7.47 (t, *J* = 7.1 Hz, 2H), 7.38 (t, *J* = 6.9 Hz, 1H), 7.32 – 7.27 (m, 2H), 7.26 (d, *J* = 7.8 Hz, 2H), 7.14 – 7.09 (m, 1H), 7.09 – 6.97 (m, 5H), 6.42 (s, 1H), 5.35 (s, 2H). ^{13}C NMR (126 MHz, Chloroform-*d*) δ 166.4, 158.9, 141.3, 139.2, 139.1, 136.3, 135.1, 128.9, 128.8, 128.6, 128.4, 128.0, 127.9, 127.5,

127.4, 127.4, 127.2, 116.6, 66.1. GC-MS(EI-70eV): m/z (%) 470 (6), 303 (6), 167 (100), 157 (7), 152 (12). HRMS (ESI): calcd for $C_{28}H_{22}NaO_2Se^+$ [M+Na]$^+$: 493.0677, found: 493.0681.

4-methoxybenzyl (Z)-3-phenyl-3-(phenylselanyl)acrylate

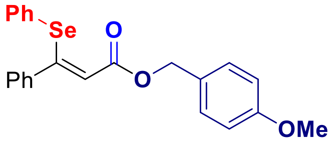

4aap

Purification by flash column chromatography (petroleum ether/EtOAc = 30:1). Yellow oily (31.0 mg, 73%).^{1}H NMR (500 MHz, Chloroform-*d*) δ 7.38 (d, *J* = 8.5 Hz, 2H), 7.22 (d, *J* = 7.1 Hz, 2H), 7.12 – 7.06 (m, 2H), 7.05 – 7.03 (m, 1H), 7.02 – 6.98 (m, 5H), 6.91 (d, *J* = 8.6 Hz, 2H), 6.35 (s, 1H), 5.22 (s, 2H), 3.82 (s, 3H). ^{13}C NMR (126 MHz, Chloroform-*d*) δ 166.5, 161.9, 159.7, 139.1, 136.3, 130.3, 129.2, 128.6, 128.4, 128.2, 128.0, 127.9, 127.5, 116.8, 114.0, 66.2, 55.3. GC-MS(EI-70eV):m/z(%) 424 (4), 303 (2), 267 (4), 157 (3), 121 (100).HRMS (ESI): calcd for $C_{23}H_{20}NaO_3Se^+$ [M+Na]$^+$:447.0470, found: 447.0467.

ethyl (Z)-3-((4-fluorophenyl)selanyl)-3-phenylacrylate

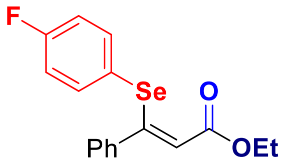

4aba

Purification by flash column chromatography (petroleum ether/EtOAc = 200:1). Yellow oily (29 mg, 83%). ^{1}H NMR (500 MHz, Chloroform-*d*) δ 7.79 – 7.74 (m, 1H), 7.71 (d, *J* = 7.7 Hz, 2H), 7.37 (t, *J* = 7.7 Hz, 1H), 7.24 – 7.18 (m, 1H), 7.15 (t, *J* = 8.6 Hz, 1H), 7.12 (d, *J* = 8.3 Hz, 1H), 7.08 (d, *J* = 7.7 Hz, 1H), 6.99 (d, *J* = 8.0 Hz, 1H), 6.57 (s, 1H), 4.32 – 4.27 (m, 2H), 1.41 (t, *J* = 7.5 Hz, 3H). ^{13}C NMR (126 MHz, Chloroform-*d*) δ 166.0, 166.0, 164.2, 138.4(d, *J* = 12.6 Hz), 130.6, 129.4, 128.9(d, *J* = 8.8 Hz), 128.6, 128.5, 127.9, 127.6, 127.2, 116.5, 116.4 (d, *J* = 22.7 Hz), 60.6, 14.4. ^{19}F NMR (471 MHz, Chloroform-*d*) δ -109.18. GC-MS(EI-70eV): m/z (%) 350 (43), 304 (11), 277 (8), 147 (100), 102 (13). HRMS (ESI): calcd for $C_{17}H_{15}NaFO_2Se^+$ [M+Na]$^+$: 373.0114, found: 373.0111.

ethyl (Z)-3-((4-chlorophenyl)selanyl)-3-phenylacrylate

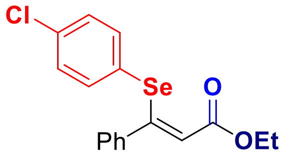

4aca

Purification by flash column chromatography (petroleum ether/EtOAc = 200:1). White oily (29.6 mg, 81%). ^{1}H NMR (500 MHz, Chloroform-d) δ 7.14 (d, J = 8.3 Hz, 2H), 7.09 (d, J = 5.1 Hz, 1H), 7.09 – 7.07 (m, 1H), 7.06 (d, J = 6.4 Hz, 1H), 7.02 – 7.00 (m, 1H), 7.00 – 6.98 (m, 1H), 6.97 (t, J = 2.0 Hz, 1H), 6.95 (t, J = 1.5 Hz, 1H), 6.33 (s, 1H), 4.30 (q, J = 7.1 Hz, 2H), 1.35 (t, J = 7.1 Hz, 3H). ^{13}C NMR (126 MHz, Chloroform-d) δ 166.7, 160.5, 138.8, 137.4, 134.4, 128.6, 128.6, 128.3, 127.7, 117.4, 60.6, 14.4.. GC-MS(EI-70eV): m/z (%) 366(37), 320(10), 293(8), 175(41), 147(100). HRMS (ESI): calcd for $C_{17}H_{15}NaClO_2Se^+$ [M+Na]$^+$: 388.9818, found: 388.9821.

ethyl (Z)-3-(phenylselanyl)-3-(m-tolyl)acrylate

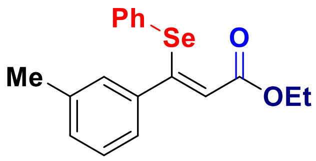

4baa

Purification by flash column chromatography (petroleum ether/EtOAc = 200:1). White oily (28 mg, 81%). ^{1}H NMR (500 MHz, Chloroform-*d*) δ 7.23 (d, *J* = 7.0 Hz, 2H), 7.08 (t, *J* = 7.4 Hz, 1H), 7.00 (t, *J* = 7.5 Hz, 2H), 6.92 (t, *J* = 7.6 Hz, 1H), 6.87 – 6.81 (m, 2H), 6.78 (s, 1H), 6.32 (s, 1H), 4.30 (q, *J* = 7.1 Hz, 2H), 2.10 (s, 3H), 1.35 (t, *J* = 7.1 Hz, 3H). ^{13}C NMR (126 MHz, Chloroform-*d*) δ 166.8, 161.5, 138.9, 137.0, 136.3, 129.6, 129.4, 128.6, 128.2, 127.9, 127.4, 125.6, 116.7, 60.5, 21.0, 14.4. GC-MS(EI-70eV): m/z (%) 346 (61), 273 (26), 189 (48), 157 (20), 115 (100). HRMS (ESI): calcd for $C_{18}H_{18}NaO_2Se^+$ [M+Na]$^+$: 369.0364, found: 369.0358.

ethyl (Z)-3-(4-methoxyphenyl)-3-(phenylselanyl)acrylate

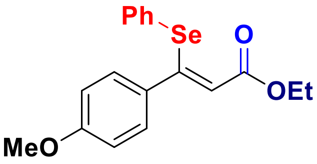

4caa

Purification by flash column chromatography (petroleum ether/EtOAc = 50:1). White solid (22.4 mg, 62%). ^{1}H NMR (500 MHz, Chloroform-*d*) δ 7.23 (d, *J* = 7.0 Hz, 2H), 7.10 (t, *J* = 7.4 Hz, 1H), 7.01 (t, *J* = 7.4 Hz, 2H), 6.97 (d, *J* = 8.7 Hz, 2H), 6.56 (d, *J* = 8.7 Hz, 2H), 6.30 (s, 1H), 4.29 (q, *J* = 7.1 Hz, 2H), 3.69 (s, 3H), 1.35 (t, *J* = 7.1 Hz, 3H). ^{13}C NMR (126 MHz, Chloroform-*d*) δ 166.8, 160.8, 159.5, 136.0, 131.7, 130.1, 129.6, 128.4, 127.8, 116.7, 112.9, 60.4, 55.2, 14.4. GC-MS(EI-70eV): m/z (%) 362 (19), 317 (5), 289 (1), 205 (100), 157 (6). HRMS (ESI): calcd for $C_{18}H_{18}NaO_3Se^+$ [M+Na]$^+$: 385.0313, found: 385.0307.

ethyl (Z)-3-(4-fluorophenyl)-3-(phenylselanyl)acrylate

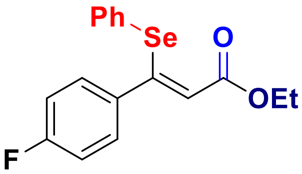

4daa

Purification by flash column chromatography (petroleum ether/EtOAc = 200:1). Yellow oily (26.6 mg, 76%). ^{1}H NMR (500 MHz, Chloroform-*d*) δ 7.22 (d, *J* = 7.1 Hz, 2H), 7.12 (t, *J* = 7.4 Hz, 1H), 7.03 (d, *J* = 7.6 Hz, 2H), 7.00 (d, *J* = 2.0 Hz, 1H), 6.99 (d, *J* = 3.2 Hz, 1H), 6.72 (t, *J* = 8.6 Hz, 2H), 6.30 (s, 1H), 4.30 (q, *J* = 7.1 Hz, 2H), 1.35 (t, *J* = 7.1 Hz, 3H). ^{13}C NMR (126 MHz, Chloroform-*d*) δ 166.6, 162.3 (d, *J* = 124.1 Hz), 160.0, 136.3, 135.2 (d, *J* = 1.9 Hz), 130.4 (d, *J* = 3.8 Hz), 129.0, 128.5, 128.1, 117.4, 114.5 (d, *J* = 10.7 Hz), 60.6, 14.4. ^{19}F NMR (471 MHz, Chloroform-*d*) δ -113.17. GC-MS(EI-70eV): m/z (%) 350 (38), 304 (22), 277 (18), 193 (31), 165 (100). HRMS (ESI): calcd for $C_{17}H_{15}NaFO_2Se^+$ [M+Na]$^+$: 373.0114, found: 373.0109.

ethyl (Z)-3-(4-chlorophenyl)-3-(phenylselanyl)acrylate

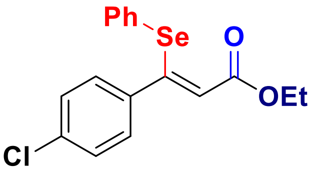

4eaa

Purification by flash column chromatography (petroleum ether/EtOAc = 200:1). White oily (28.2 mg, 77%). ^{1}H NMR (500 MHz, Chloroform-*d*) δ 7.28 – 7.21 (m, 2H), 7.15 (t, *J* = 7.4 Hz, 1H), 7.06 (d, *J* = 7.6 Hz, 2H), 7.03 (d, *J* = 8.1 Hz, 2H), 6.97 (d, *J* = 8.4 Hz, 2H), 6.32 (s, 1H), 4.32 (q, *J* = 7.1 Hz, 2H), 1.37 (t, *J* = 7.1 Hz, 3H). ^{13}C NMR (126 MHz, Chloroform-*d*) δ 166.5, 159.7, 137.7, 136.3, 134.0, 129.9, 128.9, 128.6, 128.2, 127.7, 117.5, 60.6, 14.4. GC-MS(EI-70eV): m/z (%) 366 (58), 320 (28), 293 (25), 209 (38), 181 (100). HRMS (ESI): calcd for $C_{17}H_{15}NaClO_2Se^+$ [M+Na]$^+$: 388.9818, found: 388.9823.

eyhyl (Z)-3-(2-chlorophenyl)-3-(phenylselanyl)acrylate

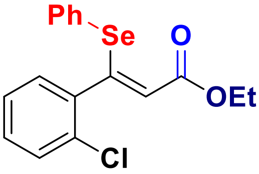

4faa

Purification by flash column chromatography (petroleum ether/EtOAc = 200:1). White oily (22.3 mg, 61%). ^{1}H NMR (500 MHz, Chloroform-*d*) δ 7.36 (d, *J* = 6.9 Hz, 2H), 7.27 (d, *J* = 13.9 Hz, 1H), 7.11 (t, *J* = 7.4 Hz, 1H), 7.05 (d, *J* = 7.4 Hz, 1H), 7.00 (t, *J* = 7.6 Hz, 2H), 6.97 (t, *J* = 3.3 Hz, 1H), 6.96 – 6.94 (m, 1H), 6.27 (s, 1H), 4.33 (q, *J* = 7.2 Hz, 2H), 1.38 (t, *J* = 7.2 Hz, 3H). ^{13}C NMR (126 MHz, Chloroform-*d*) δ 166.8, 159.0, 137.8, 137.0, 131.9, 130.4, 129.0, 129.0, 128.5, 128.1, 127.9, 125.7, 117.2, 60.6, 14.4. GC-MS(EI-70eV): m/z (%) 366 (34), 331 (100), 209 (10), 181 (68), 157 (30). HRMS (ESI): calcd for $C_{17}H_{15}NaClO_2Se^+$ [M+Na]$^+$: 388.9818, found: 388.9822.

ethyl (Z)-3-(phenylselanyl)-3-(4-(trifluoromethyl)phenyl)acrylate

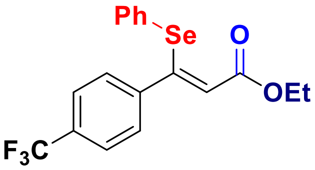

4gaa

Purification by flash column chromatography (petroleum ether/EtOAc = 200:1). White solid (30.0 mg, 75%). ^{1}H NMR (500 MHz, Chloroform-*d*) δ 7.33 – 7.27 (m, 2H), 7.23 (d, *J* = 7.1 Hz, 2H), 7.12 (d, *J* = 8.2 Hz, 3H), 7.01 (t, *J* = 7.6 Hz, 2H), 6.35 (s, 1H), 4.33 (q, *J* = 7.1 Hz, 2H), 1.38 (t, *J* = 7.1 Hz, 3H). ^{13}C NMR (101 MHz, $CDCl_3$) δ 166.4, 159.4, 142.6, 136.4, 130.0(q, *J* = 37.8 Hz), 129.7(q, *J* = 50.4 Hz,), 128.9, 128.7, 128.6, 128.5, 128.3, 128.1, 124.4(q,

J = 3.8 Hz), 117.7, 60.7, 14.4. ^{19}F NMR (471 MHz, Chloroform-*d*) δ -62.87. GC-MS(EI-70eV): m/z (%) 400 (100), 355 (25), 327 (90), 171 (45), 157 (49). HRMS (ESI): calcd for $C_{18}H_{15}NaF_3O_2Se^+$ $[M+Na]^+$: 423.0082, found: 423.0079.

ethyl (Z)-3-(phenylselanyl)-3-(4-(trifluoromethoxy)phenyl)acrylate

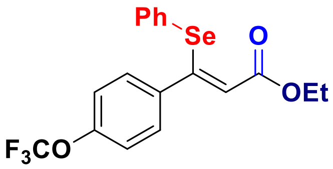

4haa

Purification by flash column chromatography (petroleum ether/EtOAc = 200:1). White solid(33.7 mg, 81%). ^{1}H NMR (500 MHz, Chloroform-*d*) δ 7.20 (d, *J* = 8.0 Hz, 2H), 7.10 (t, *J* = 7.4 Hz, 1H), 7.04 – 6.96 (m, 4H), 6.86 (d, *J* = 8.2 Hz, 2H), 6.32 (s, 1H), 4.30 (q, *J* = 7.1 Hz, 2H), 1.35 (t, *J* = 7.1 Hz, 3H). ^{13}C NMR (126 MHz, Chloroform-*d*) δ 166.5, 159.7, 148.6, 137.8, 136.5, 130.0, 128.7, 128.5, 128.2, 120.3 (q, *J* = 252 Hz), 120.0, 117.4, 60.7, 14.4. ^{19}F NMR (471 MHz, Chloroform-*d*) δ -58.02. GC-MS(EI-70eV): m/z (%) 416 (53), 370 (22), 259 (34), 231 (100), 187 (37). HRMS (ESI): calcd for $C_{18}H_{15}NaF_3O_3Se^+$ $[M+Na]^+$: 439.0031, found: 439.0025.

ethyl (Z)-3-(4-cyanophenyl)-3-(phenylselanyl)acrylate

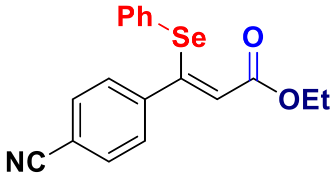

4iaa

Purification by flash column chromatography (petroleum ether/EtOAc = 50:1). White oily (20.7 mg, 58%). ^{1}H NMR (400 MHz, Chloroform-*d*) ^{1}H NMR (500 MHz, Chloroform-*d*) δ 7.65 (d, *J* = 8.3 Hz, 2H), 7.54 (d, *J* = 8.3 Hz, 2H), 7.32 (d, *J* = 8.2 Hz, 1H), 7.22 (d, *J* = 7.1 Hz, 1H), 7.17 – 7.07 (m, 2H), 7.02 (t, *J* = 7.6 Hz, 1H), 6.49 (s, 1H), 4.30 (q, *J* = 7.1 Hz, 2H), 1.34 (t, *J* = 7.1 Hz, 3H). ^{13}C NMR (101 MHz, Chloroform-*d)* δ 166.3, 165.7, 140.2, 136.5, 131.9, 131.3, 129.3, 129.1, 128.8, 128.8, 128.6, 118.7, 118.1, 111.9, 60.4, 14.4. GC-MS(EI-70eV): m/z (%) 357 (78), 312 (20), 284 (92), 157 (44), 128 (100). HRMS (ESI): calcd for $C_{18}H_{15}NNaO_2Se^+$ $[M+Na]^+$: 380.0160, found: 380.0155.

ethyl (Z)-3-(4-acetylphenyl)-3-(phenylselanyl)acrylate

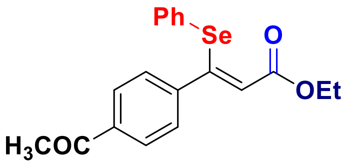

4jaa

Purification by flash column chromatography (petroleum ether/EtOAc = 30:1). White oily (27.7 mg, 74%). ^{1}H NMR (500 MHz, Chloroform-*d*) δ 7.63 (d, *J* = 8.1 Hz, 2H), 7.25 (d, *J* = 7.9 Hz, 2H), 7.12 (d, *J* = 8.2 Hz, 2H), 7.09 (t, *J* = 7.4 Hz, 1H), 7.00 (t, *J* = 7.6 Hz, 2H), 6.33 (s, 1H), 4.32 (q, *J* = 7.1 Hz, 2H), 2.49 (s, 3H), 1.37 (t, *J* = 7.1 Hz, 3H). ^{13}C NMR (126 MHz, Chloroform-*d*) δ 197.3, 166.4, 159.6, 143.8, 136.3, 136.2, 128.9, 128.6, 128.3, 128.1, 127.5, 117.7, 60.7, 26.6, 14.4. GC-MS(EI-70eV): m/z (%) 374 (87), 329 (18), 301 (31), 217 (29), 189 (100). HRMS (ESI): calcd for $C_{19}H_{18}NaO_3Se^+$ $[M+Na]^+$: 397.0313, found: 397.0309.

ethyl (Z)-3-([1,1'-biphenyl]-4-yl)-3-(phenylselanyl)acrylate

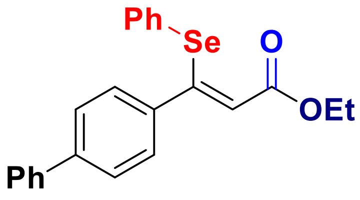

4kaa

Purification by flash column chromatography (petroleum ether/EtOAc = 200:1). White oily (31.8 mg, 78%). ^{1}H NMR (500 MHz, Chloroform-*d*) δ 7.67 – 7.59 (m, 1H), 7.47 (d, *J* = 7.2 Hz, 2H), 7.42 (t, *J* = 7.6 Hz, 2H), 7.35 (t, *J* = 7.2 Hz, 1H), 7.29 (d, *J* = 7.8 Hz, 3H), 7.11 (d, *J* = 8.0 Hz, 2H), 7.09 (d, *J* = 7.6 Hz, 1H), 7.02 (t, *J* = 7.5 Hz, 2H), 6.41 (s, 1H), 4.34 (q, *J* = 7.1 Hz, 2H), 1.39 (t, *J* = 7.1 Hz, 3H). ^{13}C NMR (126 MHz, Chloroform-*d*) δ 166.7, 160.9, 140.8, 140.3, 138.1, 136.3, 129.3, 129.1, 128.8, 128.4, 127.9, 127.5, 127.0, 126.1, 117.0, 60.6, 14.4. GC-MS(EI-70eV): m/z (%) 408 (40), 362 (10), 335 (5), 251 (100), 178 (85). HRMS (ESI): calcd for $C_{23}H_{20}NaO_2Se^+$ $[M+Na]^+$: 431.0521, found: 431.0526.

ethyl (Z)-3-(naphthalen-2-yl)-3-(phenylselanyl)acrylate

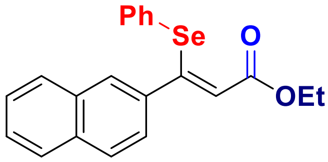

4laa

Purification by flash column chromatography (petroleum ether/EtOAc = 200:1). White oily (28.3 mg, 74%). ^{1}H NMR (500 MHz, Chloroform-*d*) δ 7.73 – 7.65 (m, 2H), 7.60 (s, 1H), 7.47 (d, *J* = 8.5 Hz, 1H), 7.45 – 7.43 (m, 1H), 7.42 (d, *J* = 4.6 Hz, 1H), 7.29 (d, *J* = 6.8 Hz, 2H), 7.12 (d, *J* = 7.0 Hz, 1H), 6.96 (t, *J* = 7.3 Hz, 1H), 6.90 (t, *J* = 7.6 Hz, 2H), 6.46 (s, 1H), 4.35 (q, *J* = 7.1 Hz, 2H), 1.39 (t, *J* = 7.1 Hz, 3H).. ^{13}C NMR (126 MHz, Chloroform-*d*) δ 166.7, 161.1, 136.8, 136.2, 132.6, 132.4, 129.1, 128.4, 128.0, 127.9, 127.8, 127.5, 127.0, 126.5, 126.4, 126.2, 117.5, 60.6, 14.4. GC-MS(EI-70eV): m/z (%) 382(54), 336 (14), 309 (14), 225 (92), 152 (100). HRMS (ESI): calcd for $C_{21}H_{18}NaO_2Se^+$ [M+Na]$^+$: 405.0364, found: 405.0363.

ethyl (Z)-3-(phenylselanyl)-3-(thiophen-3-yl)acrylate

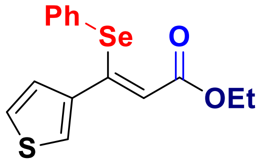

4maa

Purification by flash column chromatography (petroleum ether/EtOAc = 200:1). White oily (21.0 mg, 62%). ^{1}H NMR (500 MHz, Chloroform-*d*) ^{1}H NMR (500 MHz, Chloroform-*d*) δ 7.36 (d, *J* = 7.3 Hz, 2H), 7.19 (t, *J* = 7.3 Hz, 1H), 7.15 (d, *J* = 6.6 Hz, 1H), 7.12-7.11 (m, 2H), 6.74 (d, *J* = 3.0 Hz, 1H), 6.73 – 6.70 (m, 1H), 6.52 (s, 1H), 4.30 (q, *J* = 7.2 Hz, 2H), 1.37 (t, *J* = 7.2 Hz, 3H). ^{13}C NMR (126 MHz, $CDCl_3$) δ 166.3, 150.3, 140.7, 135.3, 130.1, 129.3, 128.7, 127.9, 126.8, 126.7, 118.5, 60.6, 14.4. GC-MS(EI-70eV): m/z (%) 338 (26), 292 (10), 181 (48), 153 (100), 109 (63). HRMS (ESI): calcd for $C_{15}H_{14}NaO_2SSe^+$ [M+Na]$^+$: 360.9772, found: 360.9775.

ethyl (Z)-4-phenyl-3-(phenylselanyl)but-2-enoate

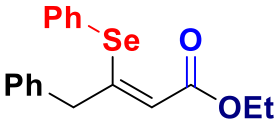

4naa

Purification by flash column chromatography (petroleum ether/EtOAc = 200:1). White oily (22.5 mg, 65%). ^{1}H NMR (500 MHz, Chloroform-*d*) δ 7.56 (d, *J* = 7.1 Hz, 2H), 7.39 (t, *J* = 7.4 Hz, 1H), 7.36 – 7.32 (m, 1H), 7.28 (t, *J* = 7.6 Hz, 2H), 7.21 (t, *J* = 5.2 Hz, 2H), 6.91 (d, *J* = 2.2 Hz, 1H), 6.90 – 6.86 (m, 1H), 6.04 (s, 1H), 4.26 (q, *J* = 7.1 Hz, 2H), 3.51 (s, 2H), 1.33 (t, *J* = 7.1 Hz, 3H). ^{13}C NMR (126 MHz, Chloroform-*d*) δ 167.0, 161.1, 137.8, 137.5, 129.2, 129.0, 128.8, 128.4, 127.3, 126.6, 116.2, 60.3, 44.1, 14.4. GC-MS(EI-70eV): m/z (%) 346 (9), 300 (3), 189 (15), 157 (5), 115 (100). HRMS (ESI): calcd for $C_{18}H_{19}O_2Se^+$ [M+H]$^+$: 347.0545, found: 347.0547.

ethyl (Z)-3-(phenylselanyl)non-2-enoate

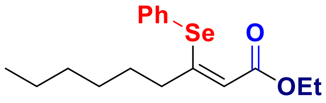

4oaa

Purification by flash column chromatography (petroleum ether/EtOAc = 200:1). White solid (21.1 mg, 62%). ^{1}H NMR (500 MHz, Chloroform-*d*) δ 7.67 (d, *J* = 7.0 Hz, 2H), 7.40 (t, *J* = 7.3 Hz, 1H), 7.34 (t, *J* = 7.3 Hz, 2H), 6.16 (s, 1H), 4.23 (q, *J* = 7.2 Hz, 2H), 2.19 – 2.12 (m, 2H), 1.34 – 1.26 (m, 7H), 1.01 (m, 4H), 0.79 (t, *J* = 7.3 Hz, 3H). ^{13}C NMR (126 MHz, Chloroform-*d*) δ 167.2, 163.7, 137.6, 129.1, 129.0, 127.7, 113.6, 60.2, 37.8, 31.3, 29.7, 28.5, 22.4, 14.4, 14.0. GC-MS(EI-70eV): m/z (%) 340 (30), 311 (2), 295 (9), 183 (77), 109 (100). HRMS (ESI): calcd for $C_{17}H_{25}O_2Se^+$ [M+H]$^+$: 341.1014, found: 341.1011.

4. NMR Spectrum Copies

^{1}H NMR (600 MHz) Spectrum of **4aaa** in $CDCl_3$

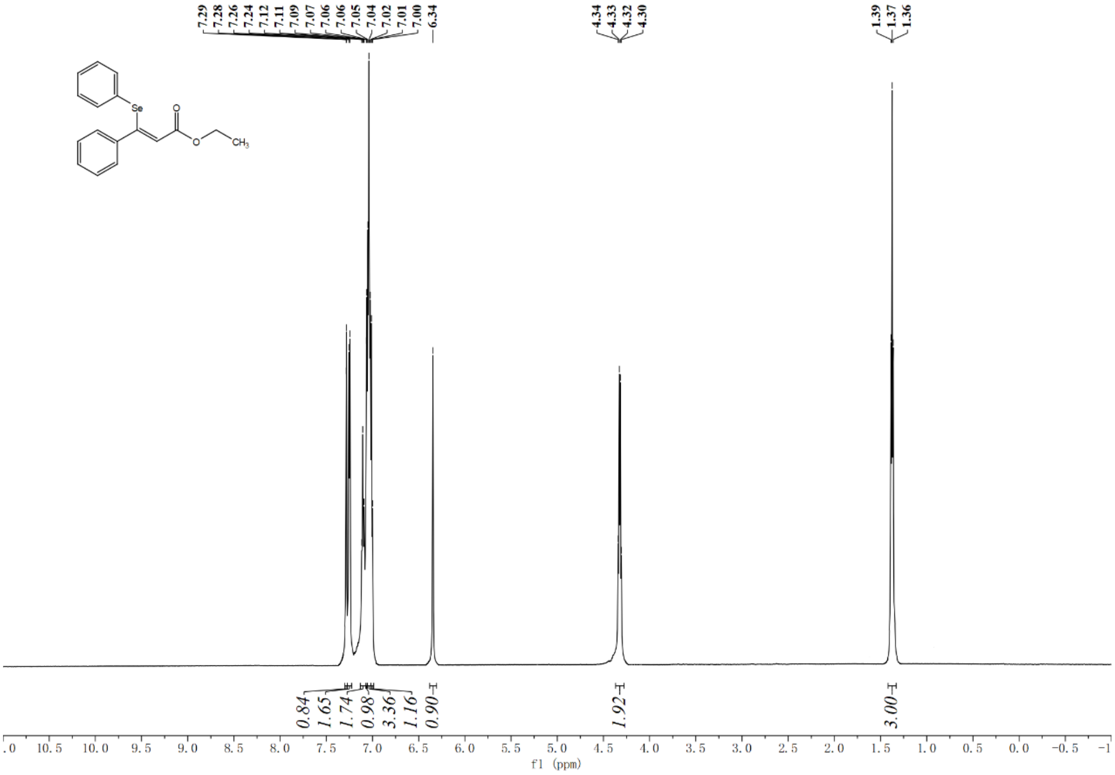

^{13}C{^{1}H} NMR (151 MHz) Spectrum of **4aaa** in $CDCl_3$

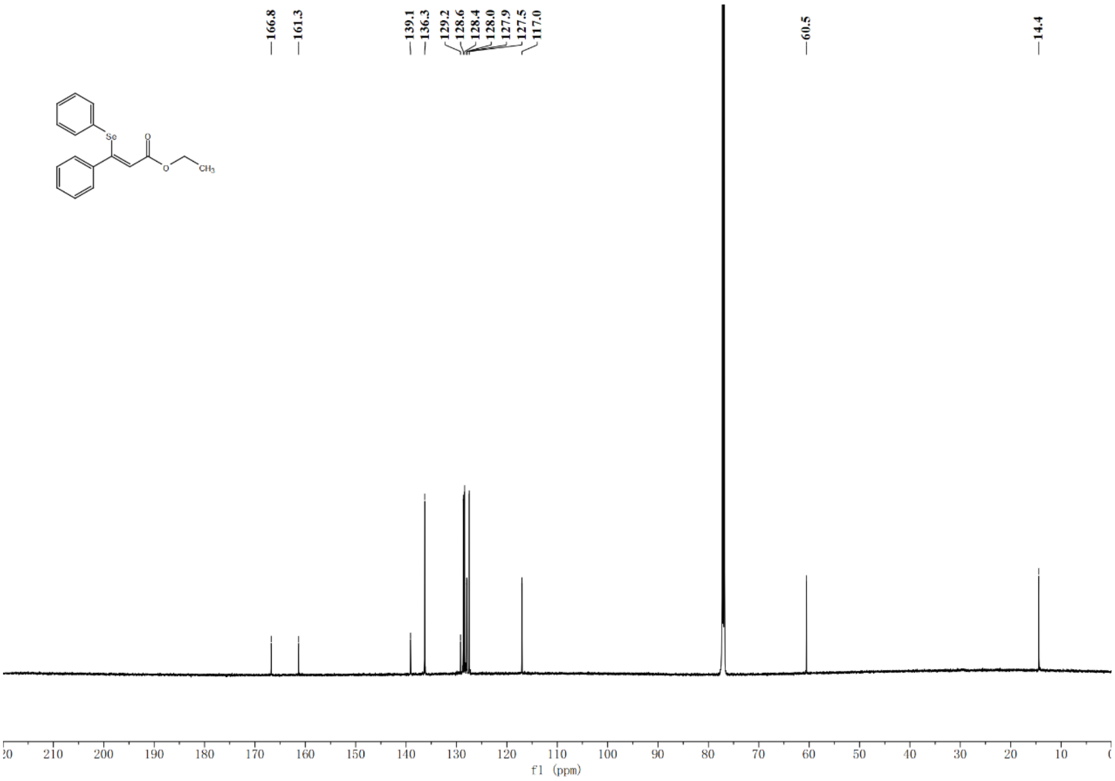

^{1}H NMR (500 MHz) Spectrum of **4aab** in $CDCl_3$

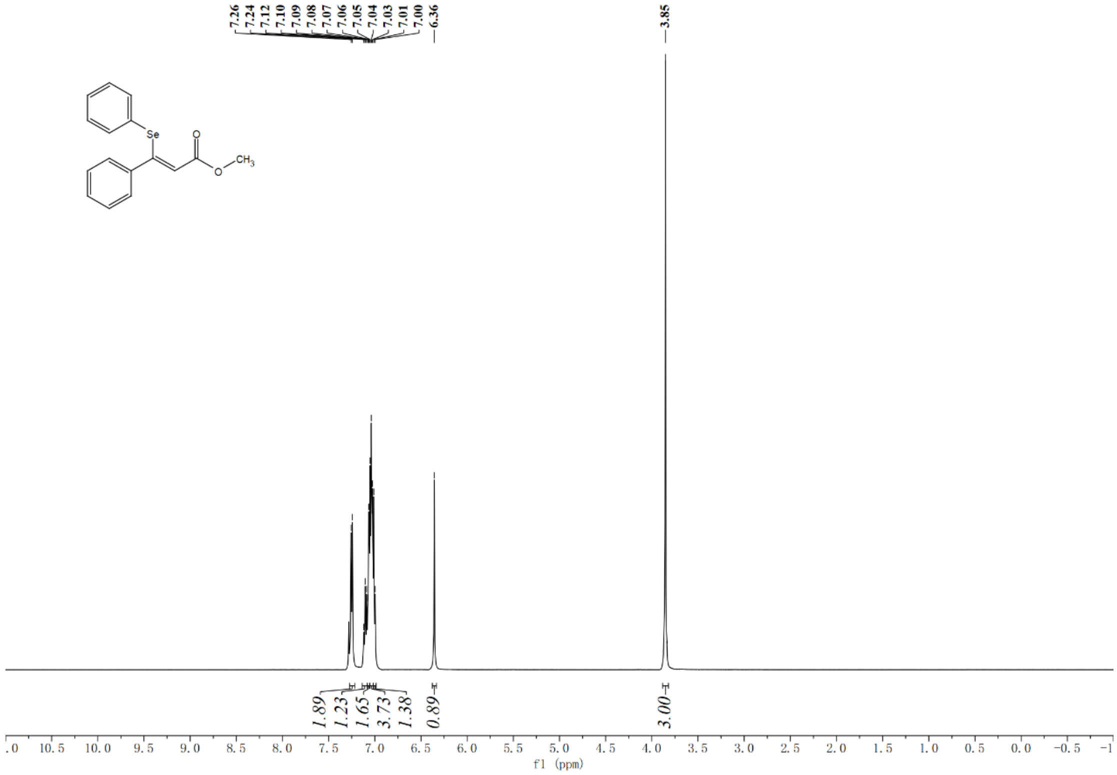

^{13}C{^{1}H} NMR (126 MHz) Spectrum of **4aab** in $CDCl_3$

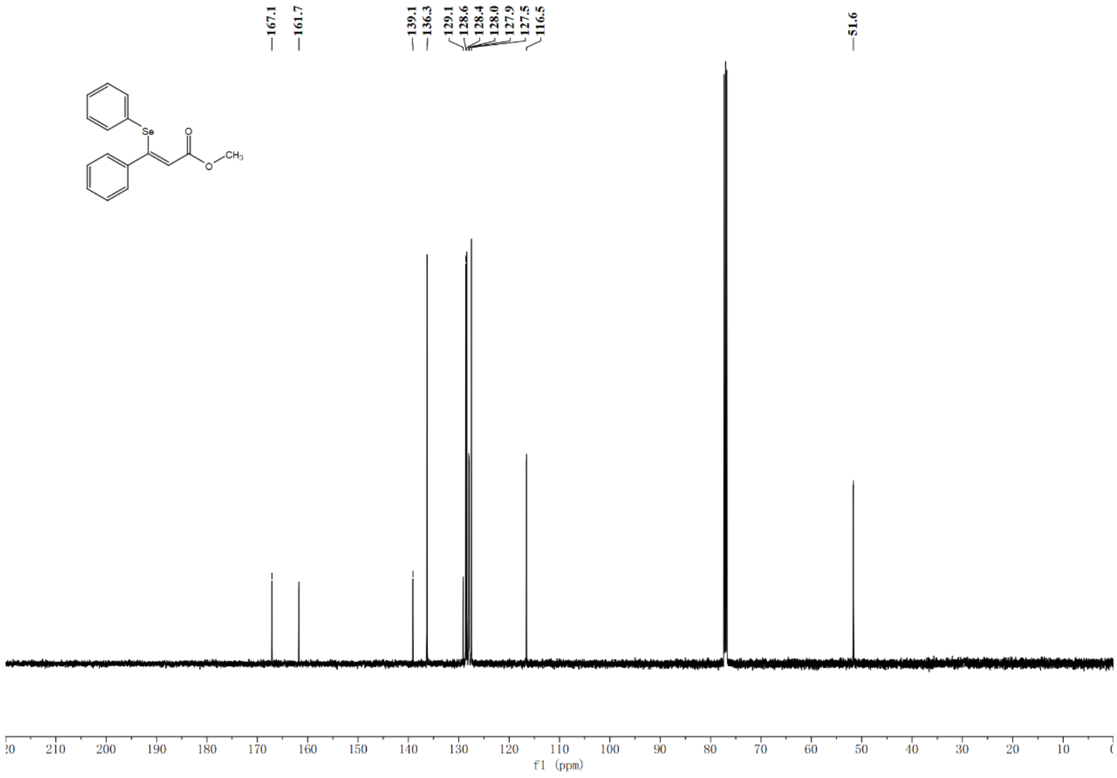

^{1}H NMR (500 MHz) Spectrum of **4aac** in $CDCl_3$

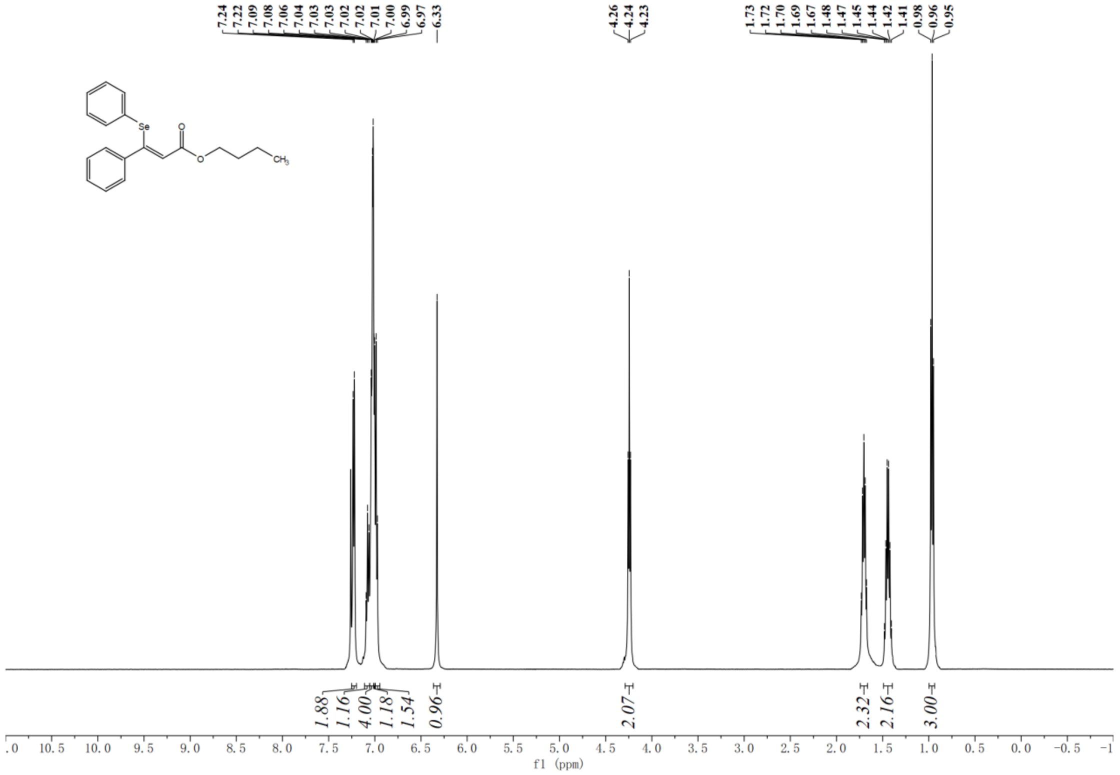

13C{1H} NMR (126 MHz) Spectrum of **4aac** in $CDCl_3$

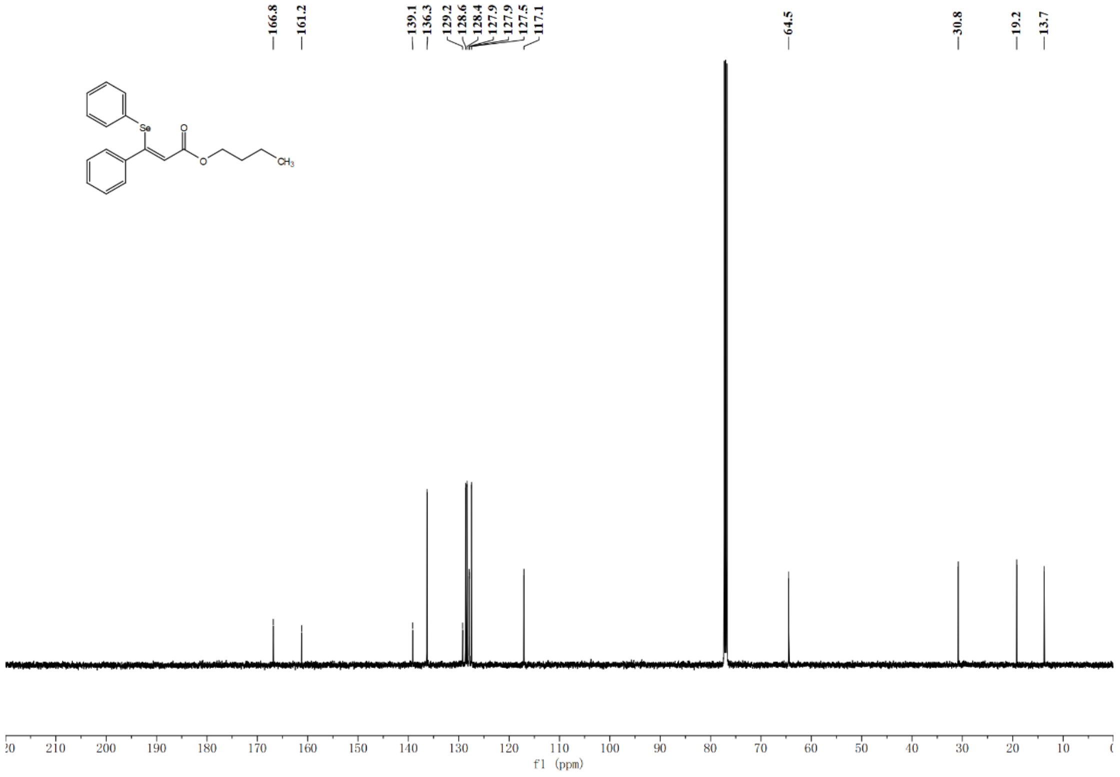

^{1}H NMR (500 MHz) Spectrum of **4aad** in $CDCl_3$

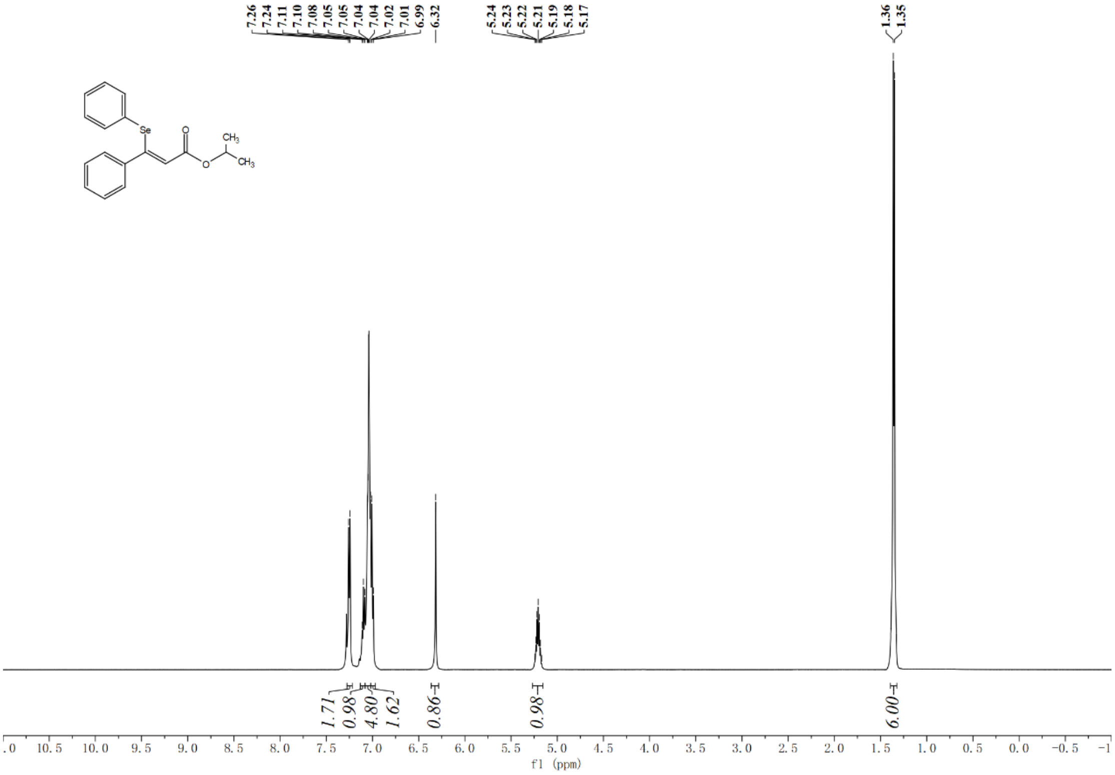

$^{13}C\{^{1}H\}$ NMR (126 MHz) Spectrum of **4aad** in $CDCl_3$

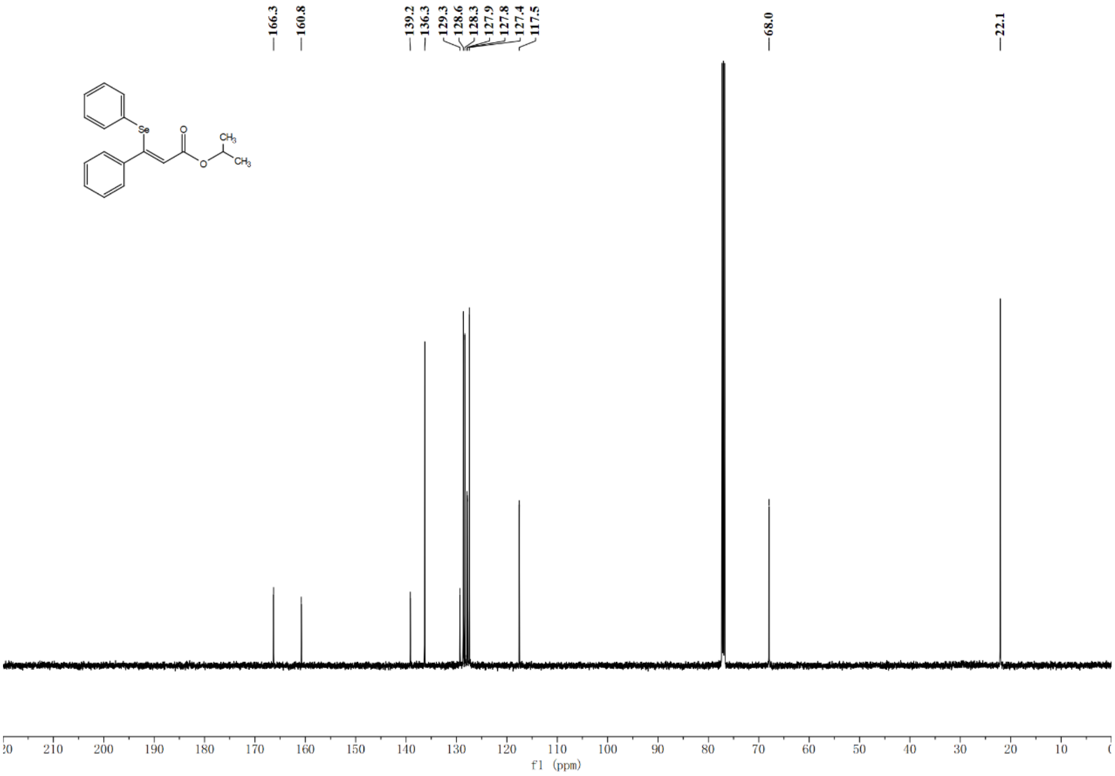

^{1}H NMR (400 MHz) Spectrum of **4aae** in $CDCl_3$

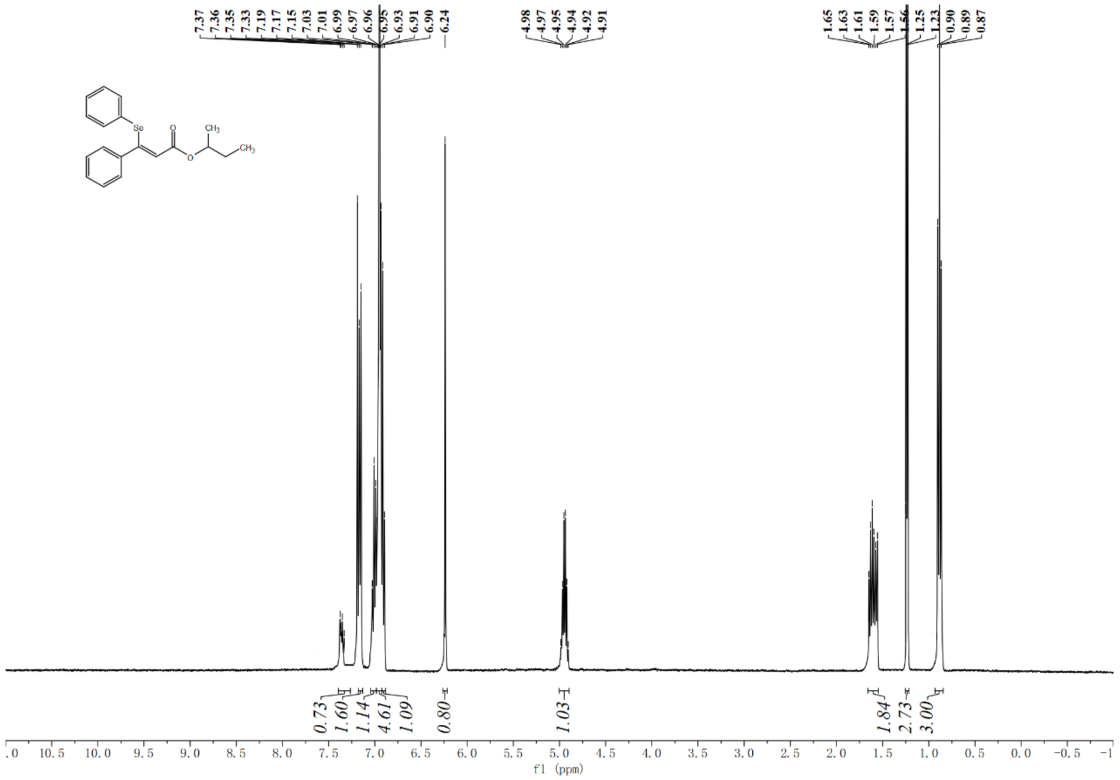

$^{13}C\{^1H\}$ NMR (101 MHz) Spectrum of **4aae** in $CDCl_3$

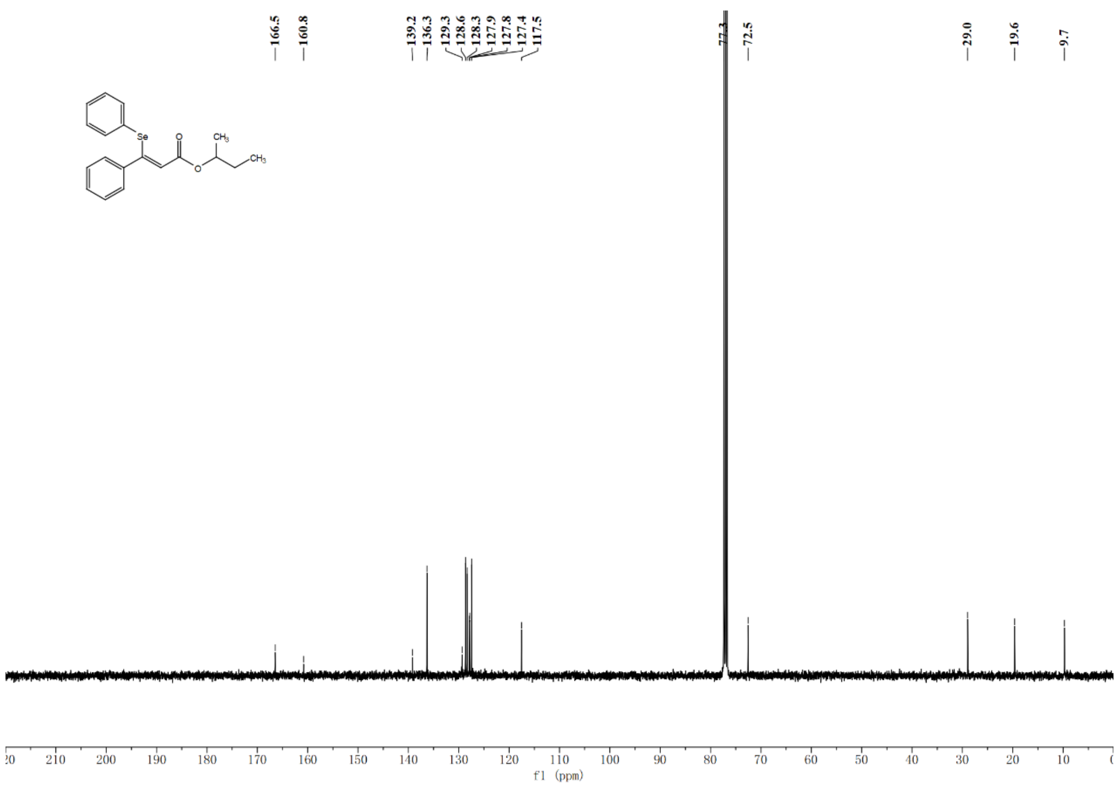

^{1}H NMR (400 MHz) Spectrum of **4aaf** in $CDCl_3$

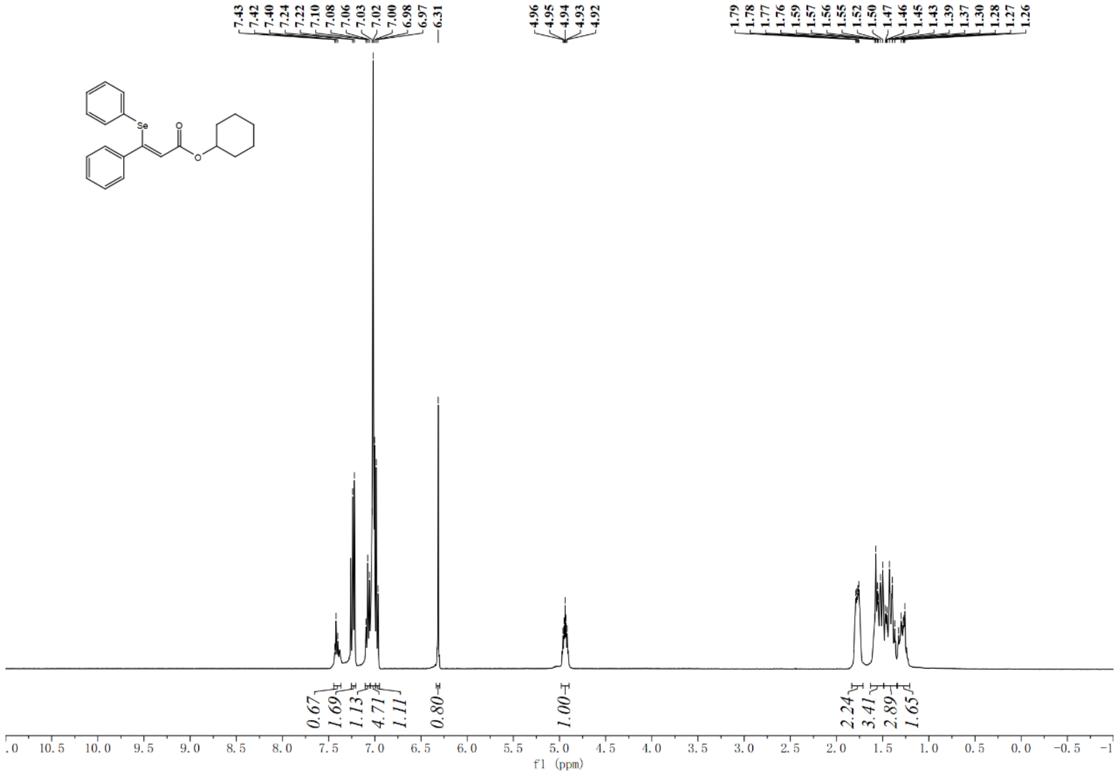

^{13}C{^{1}H} NMR (101 MHz) Spectrum of **4aaf** in $CDCl_3$

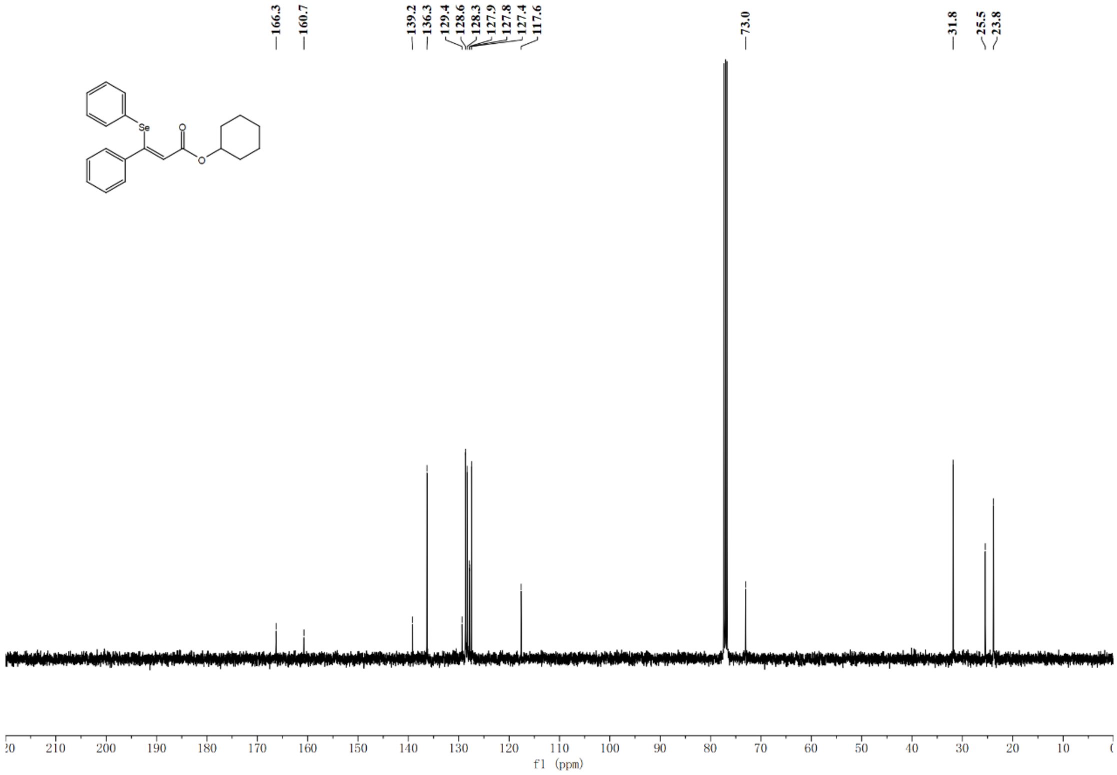

^{1}H NMR (500 MHz) Spectrum of **4aag** in $CDCl_3$

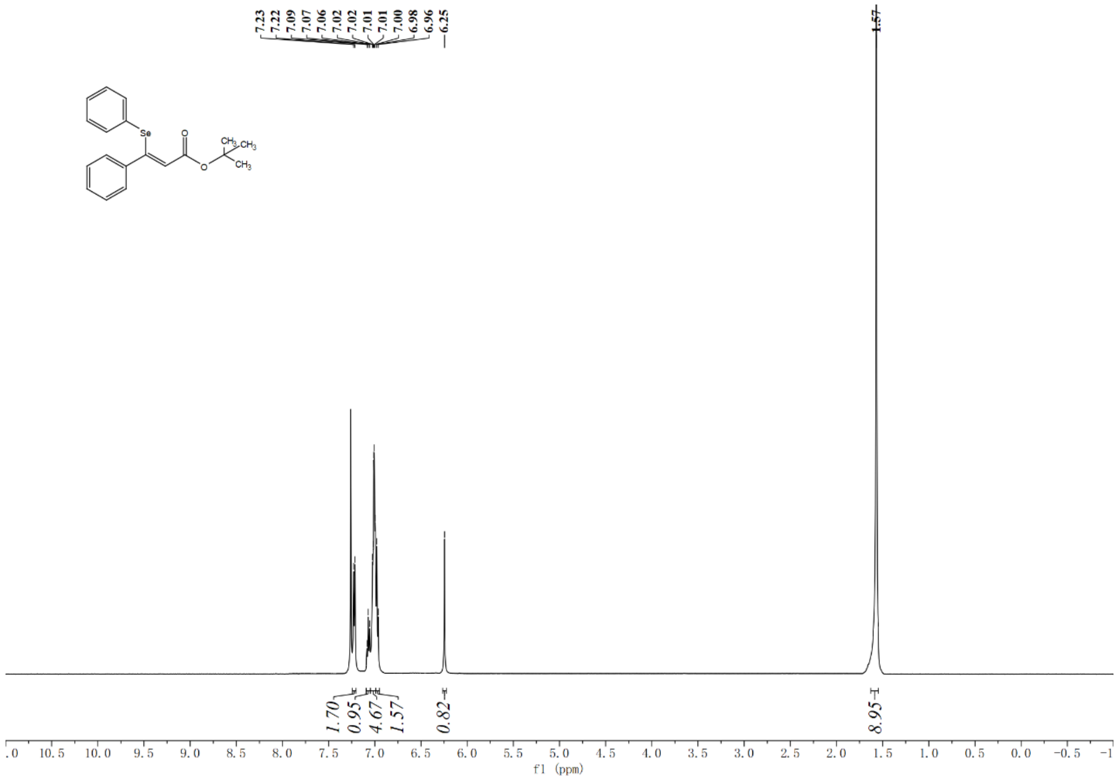

$^{13}C\{^{1}H\}$ NMR (126 MHz) Spectrum of **4aag** in $CDCl_3$

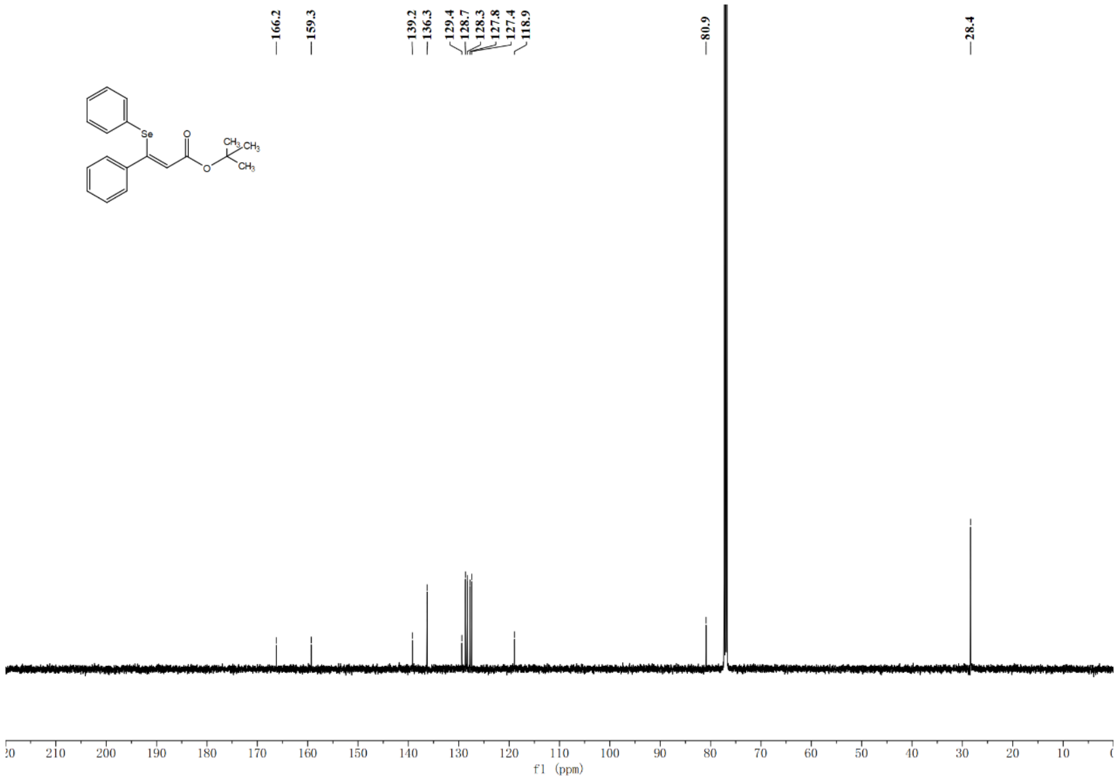

^{1}H NMR (500 MHz) Spectrum of **4aah** in $CDCl_3$

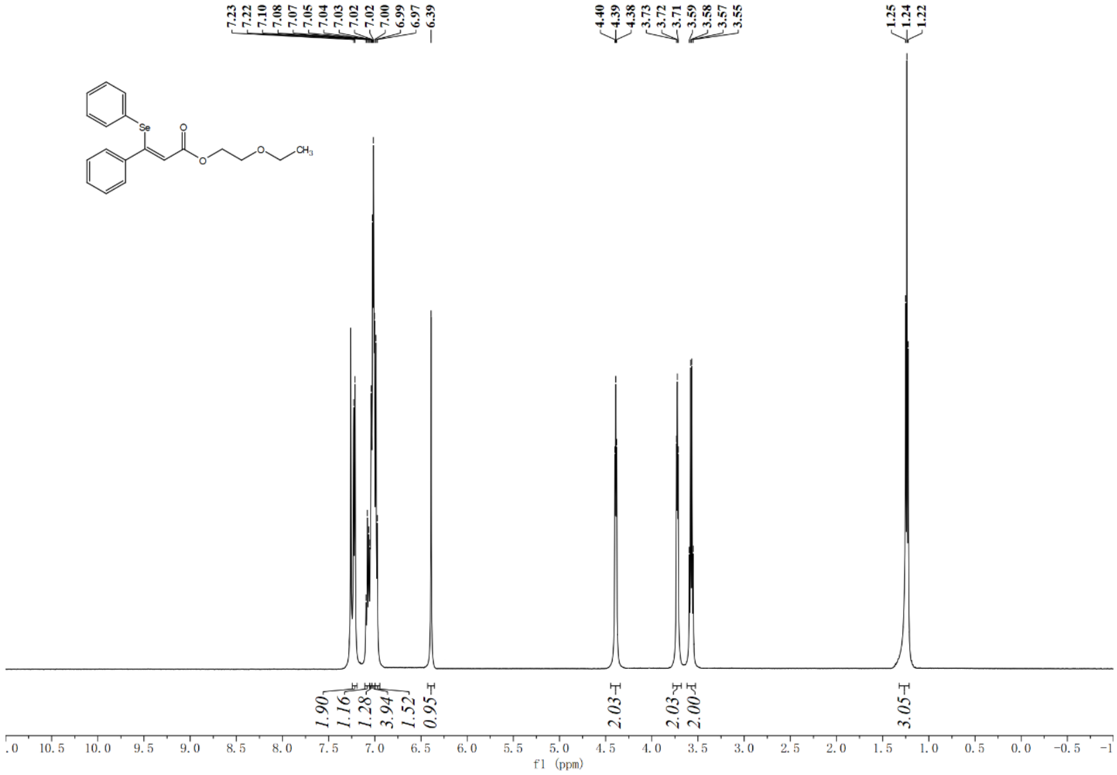

$^{13}C\{^{1}H\}$ NMR (126 MHz) Spectrum of **4aah** in $CDCl_3$

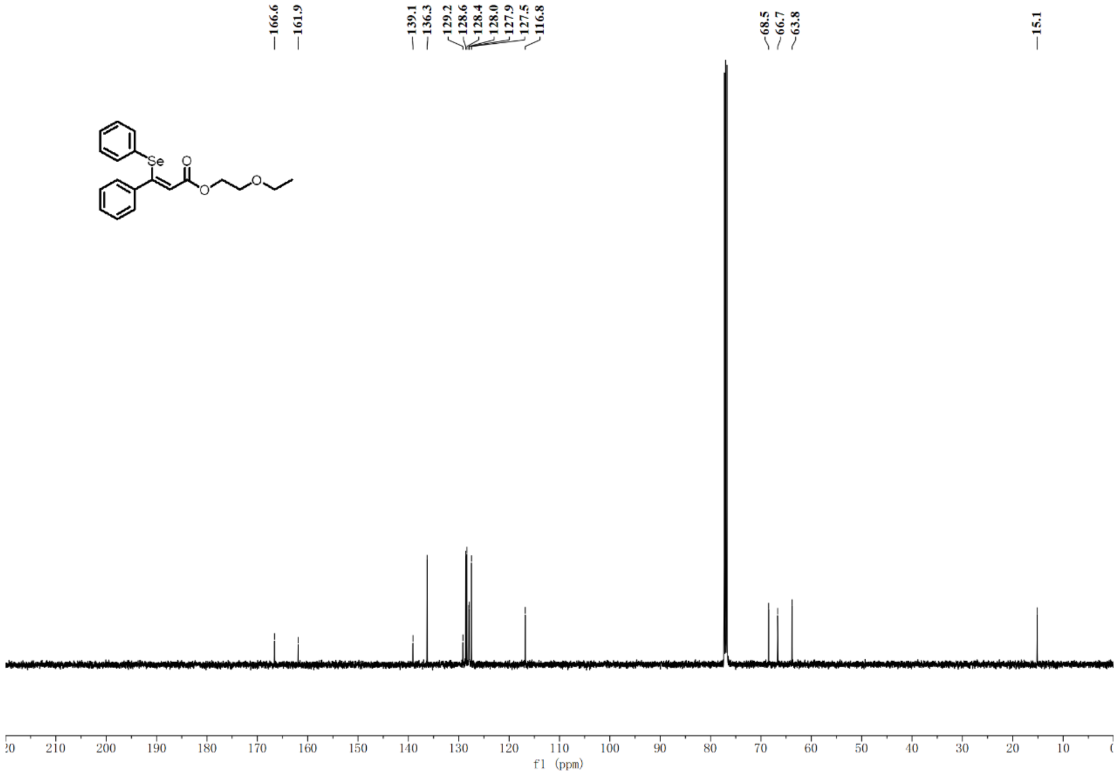

^{1}H NMR (500 MHz) Spectrum of **4aai** in $CDCl_3$

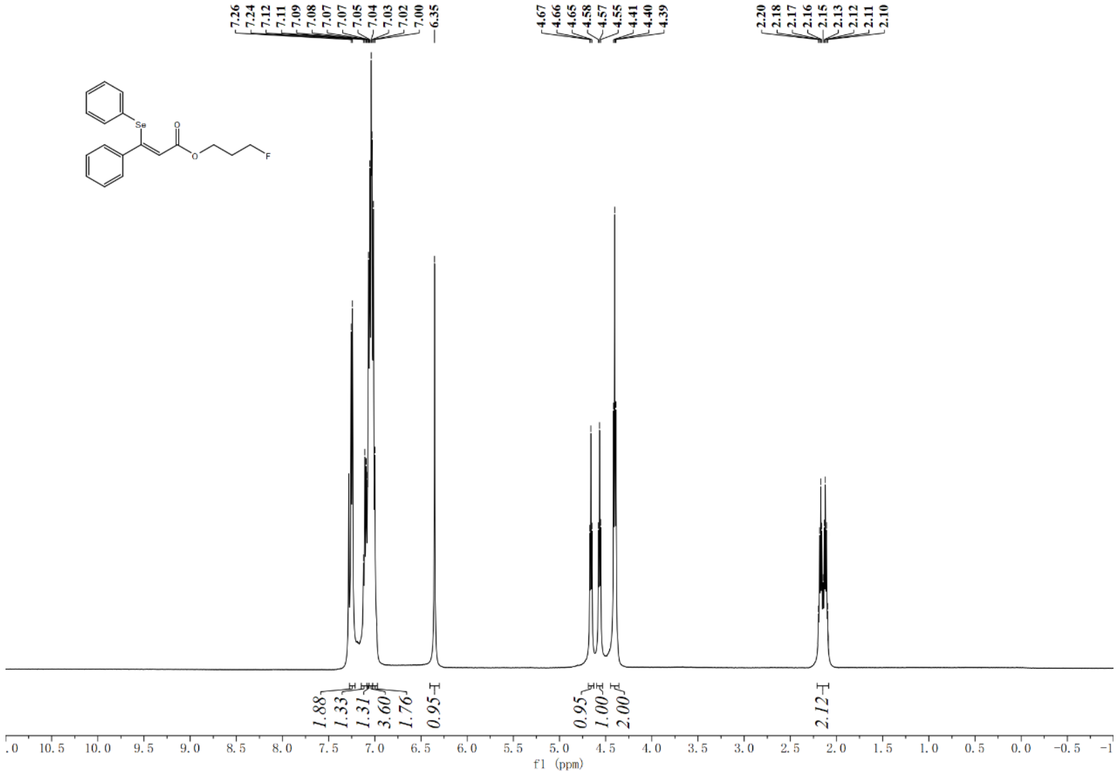

$^{13}C\{^{1}H\}$ NMR (126 MHz) Spectrum of **4aai** in $CDCl_3$

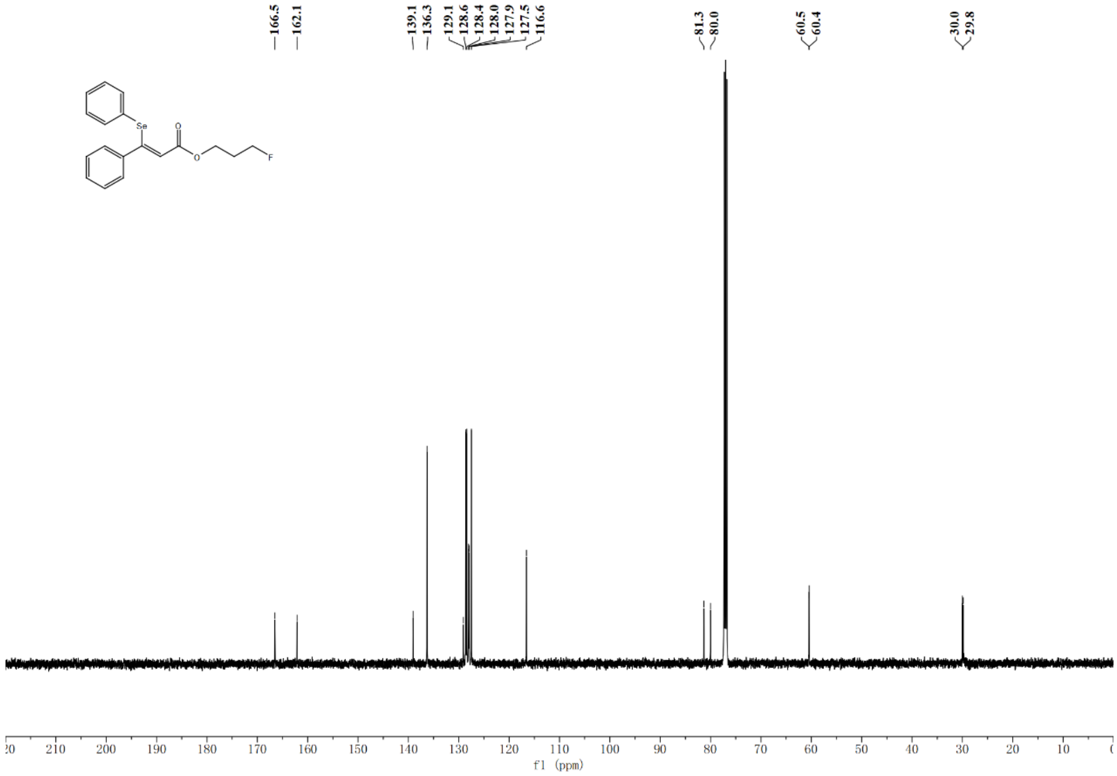

^{19}F NMR (471 MHz) Spectrum of **4aai** in $CDCl_3$

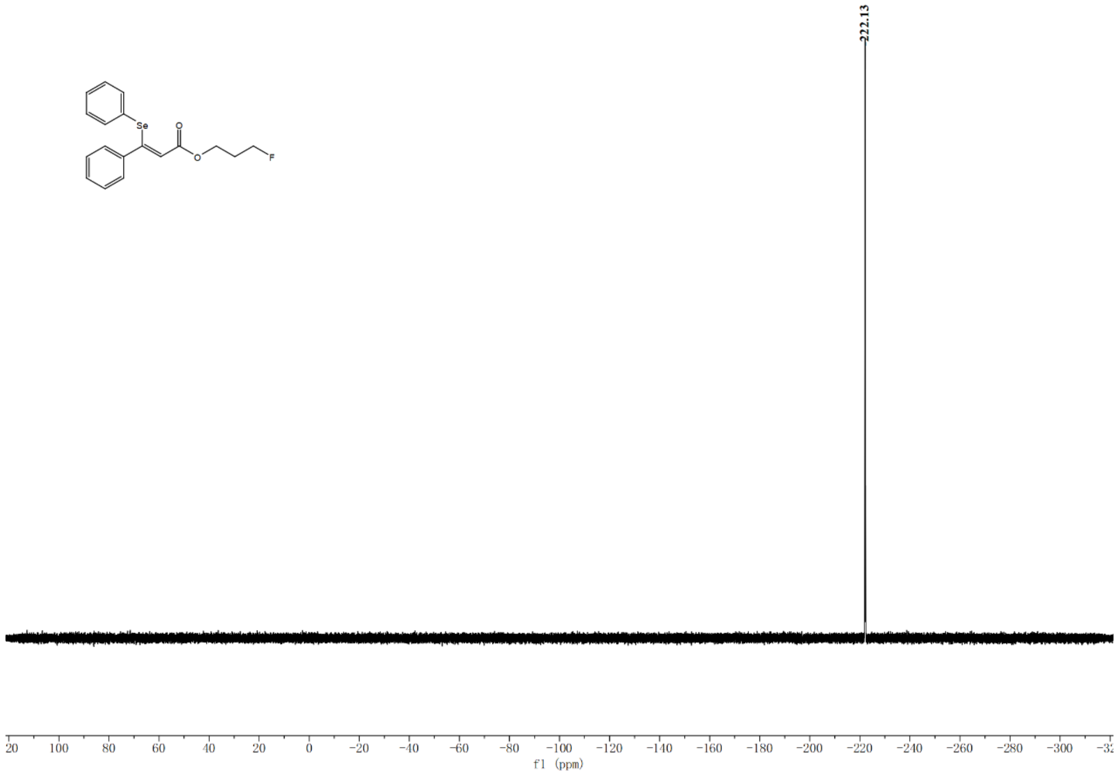

^{1}H NMR (500 MHz) Spectrum of **4aaj** in $CDCl_3$

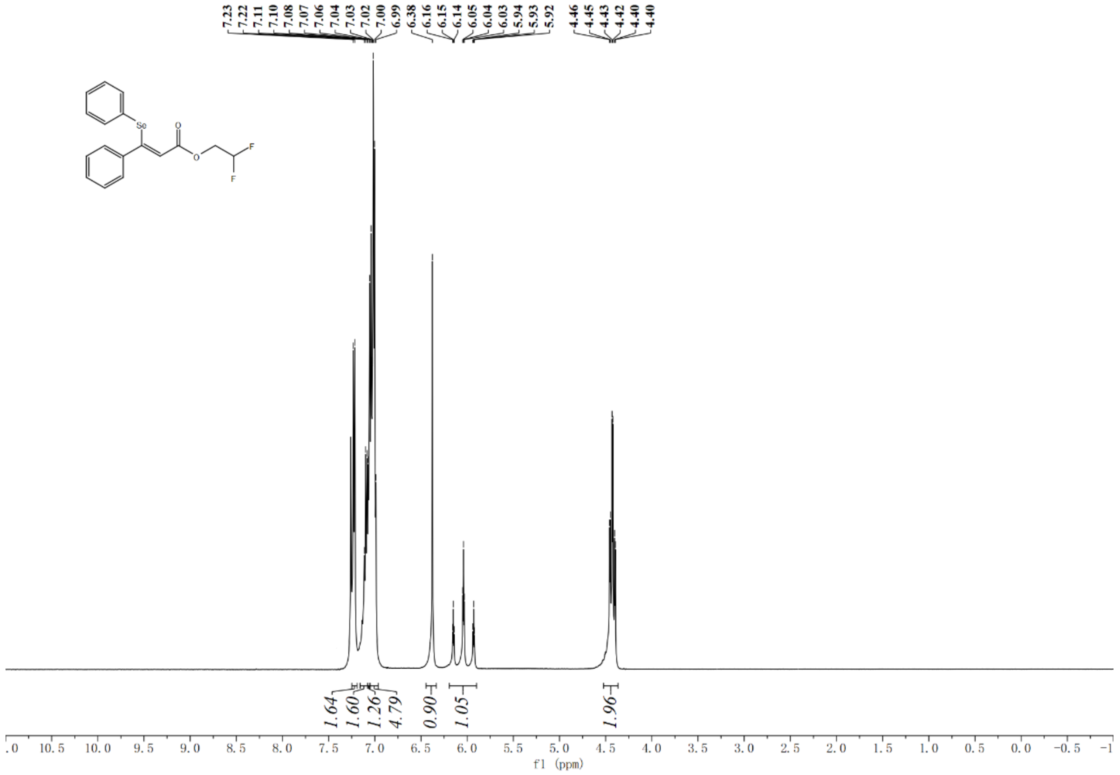

$^{13}C\{^{1}H\}$ NMR (126 MHz) Spectrum of **4aaj** in $CDCl_3$

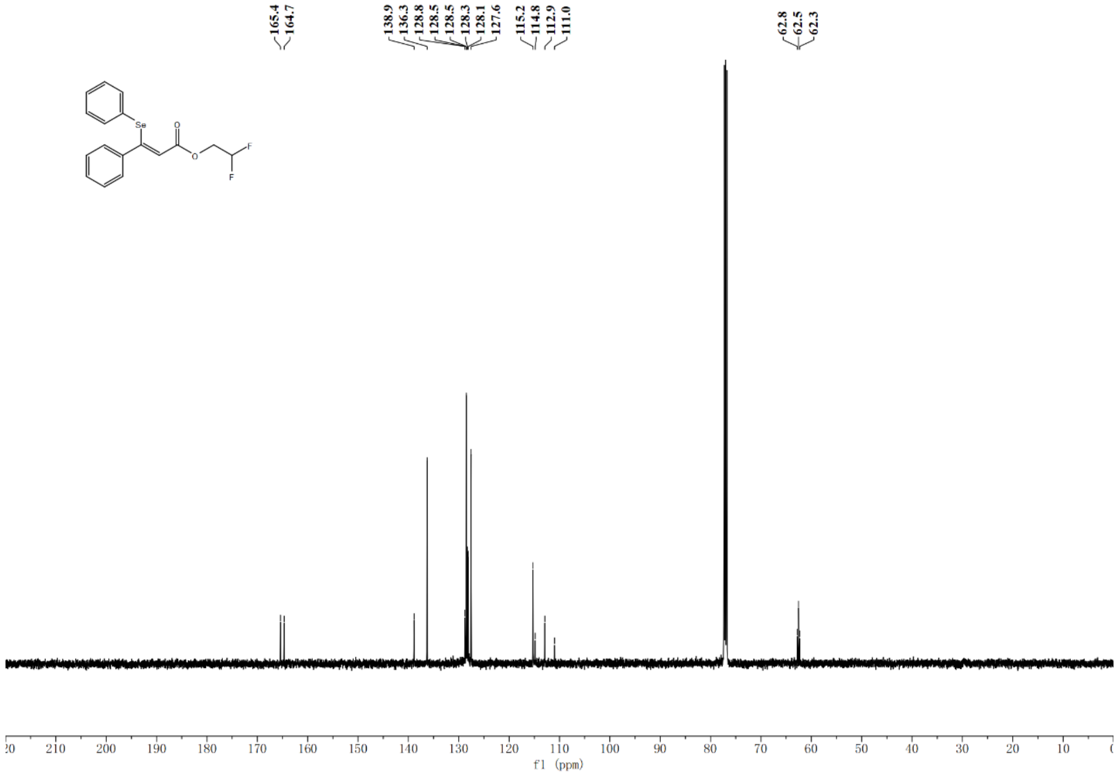

^{19}F NMR (471 MHz) Spectrum of **4aaj** in $CDCl_3$

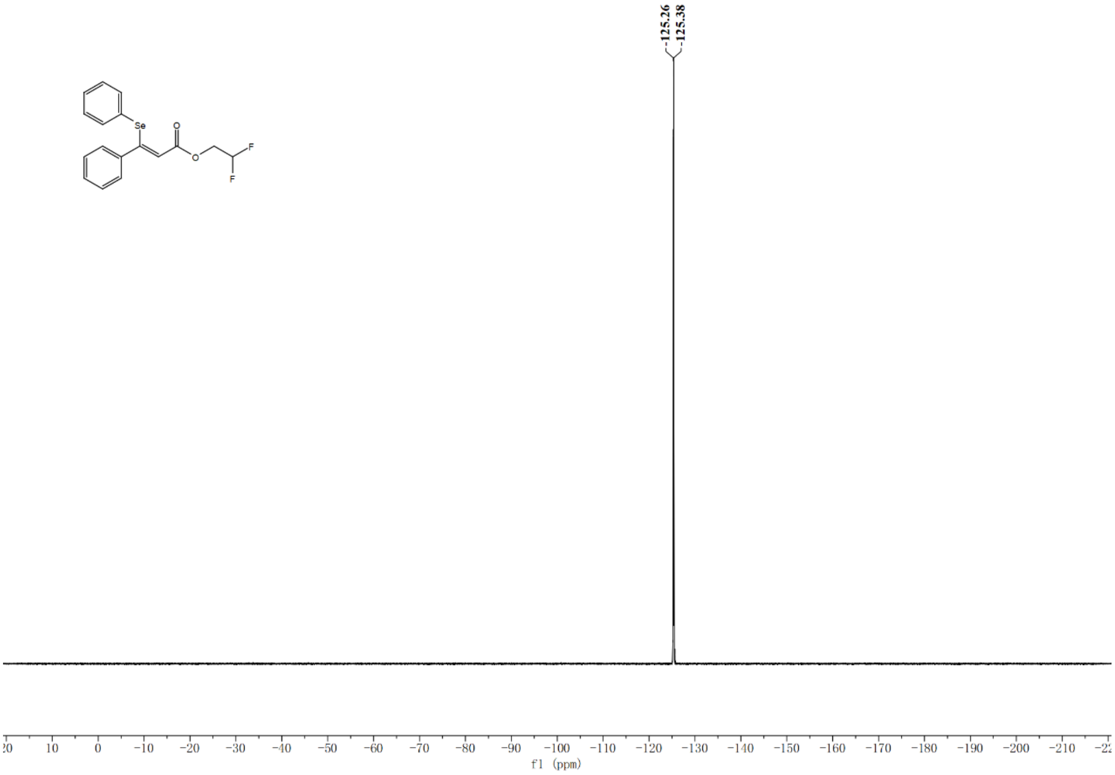

^{1}H NMR (400 MHz) Spectrum of **4aak** in $CDCl_3$

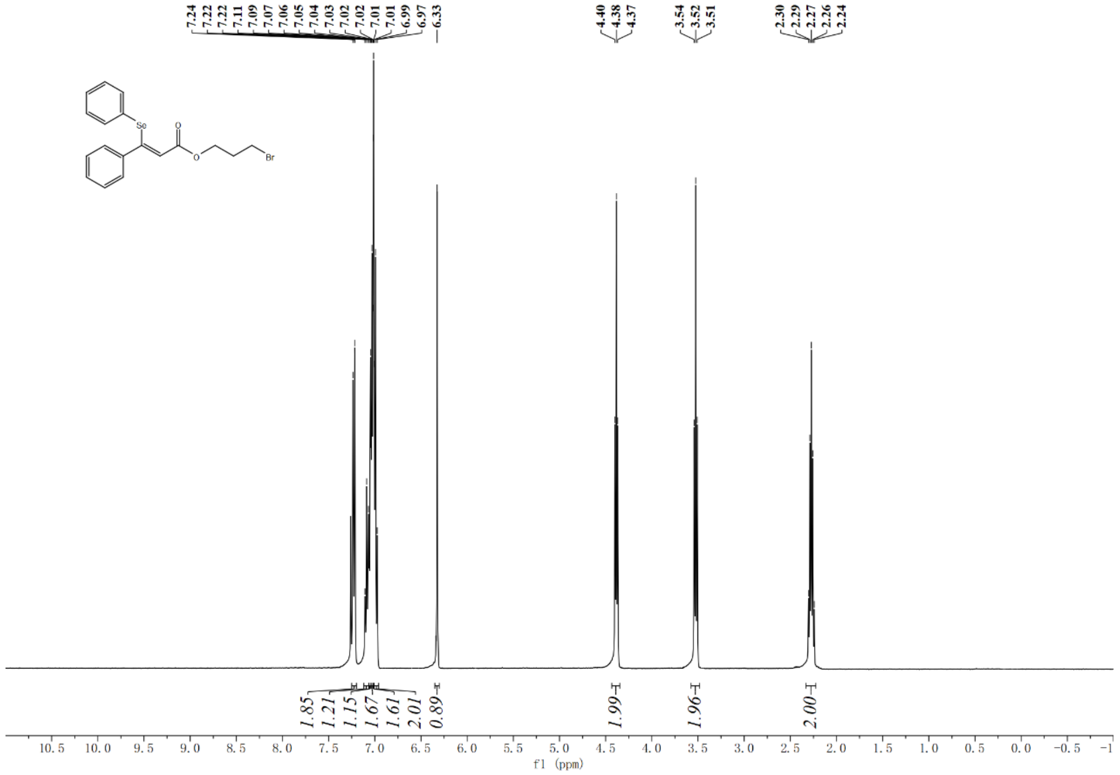

$^{13}C\{^{1}H\}$ NMR (151 MHz) Spectrum of **4aak** in $CDCl_3$

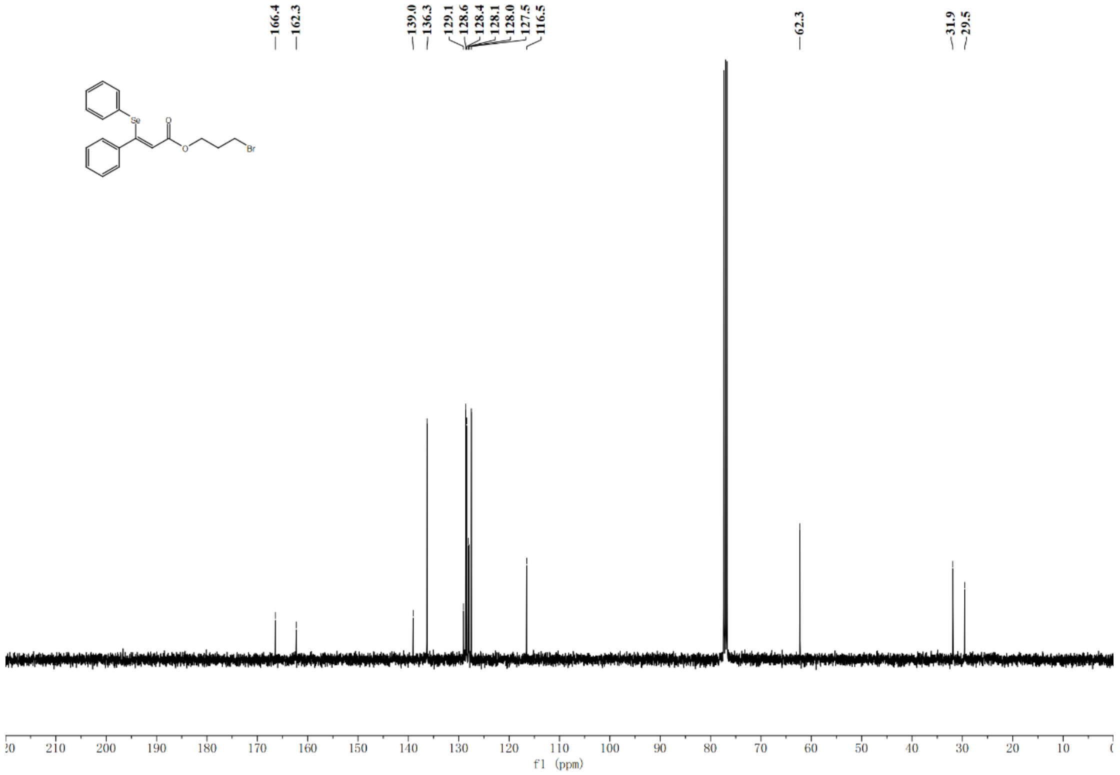

^{1}H NMR (500 MHz) Spectrum of **4aal** in $CDCl_3$

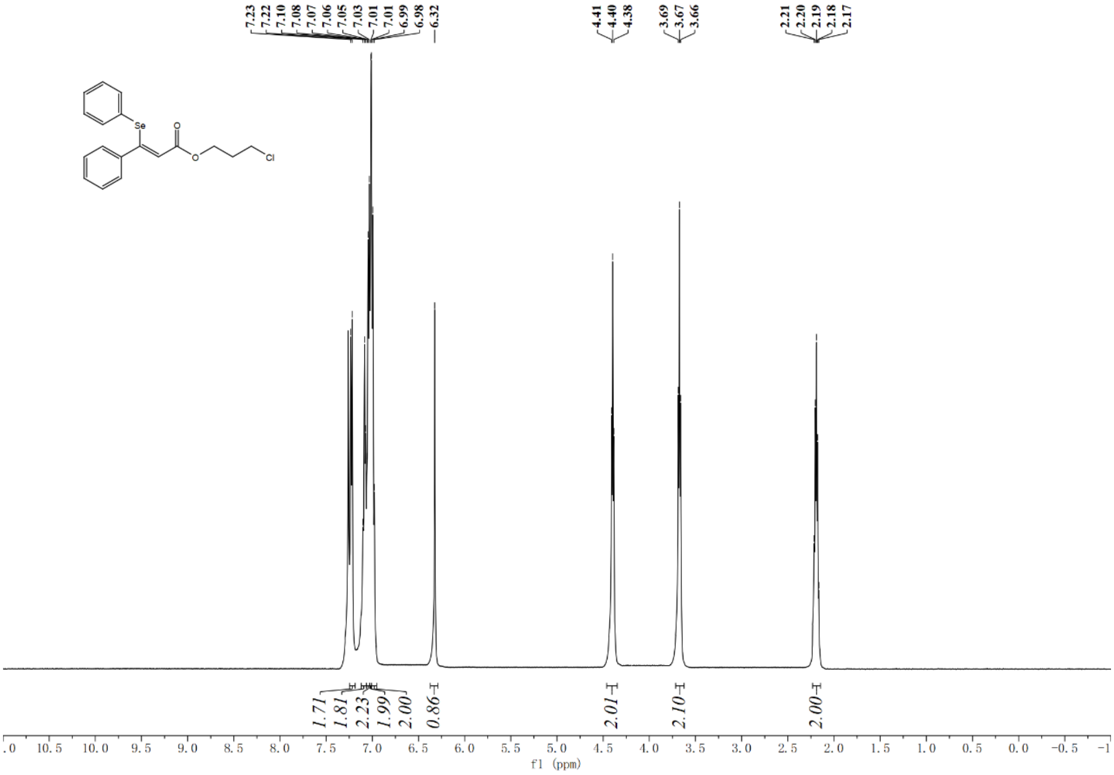

^{13}C{^{1}H} NMR (126 MHz) Spectrum of **4aal** in $CDCl_3$

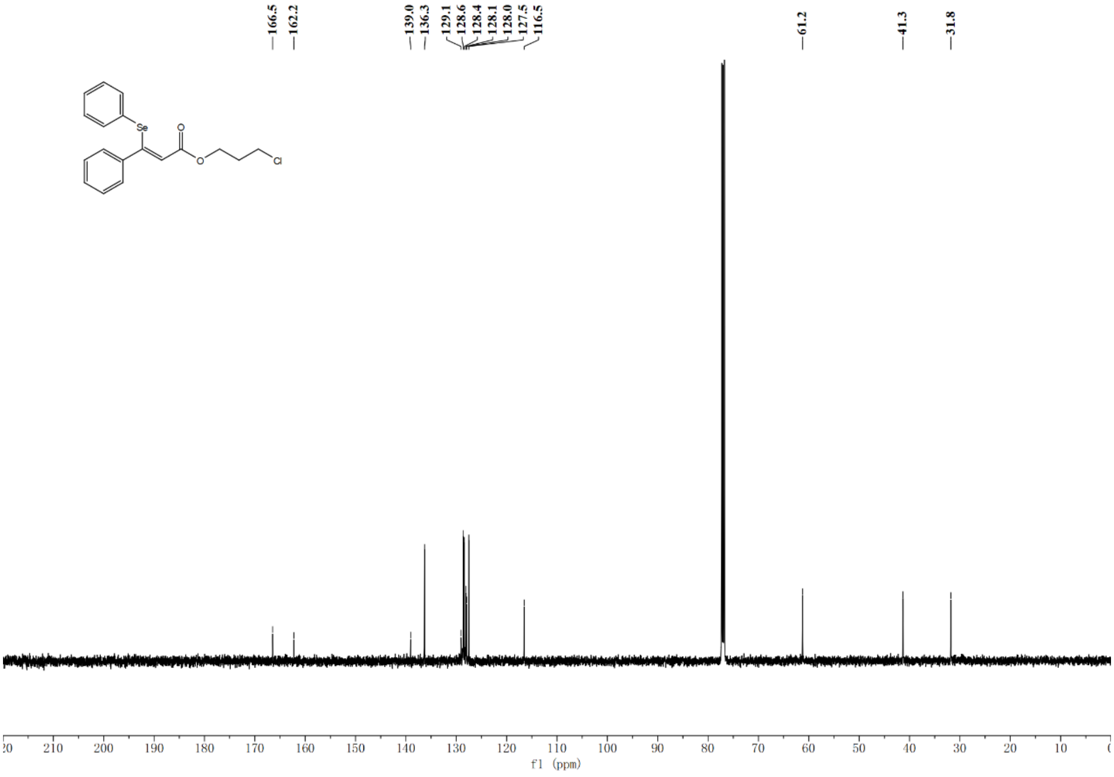

^{1}H NMR (500 MHz) Spectrum of **4aam** in $CDCl_3$

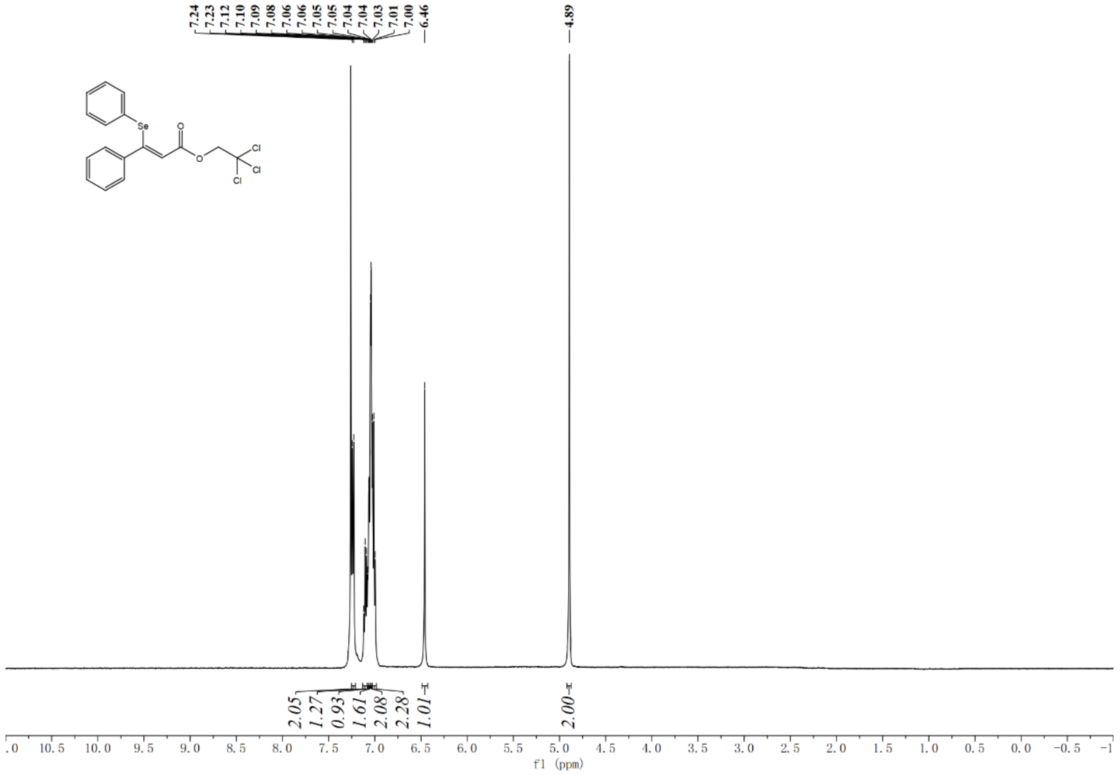

^{13}C{^{1}H} NMR (126 MHz) Spectrum of **4aam** in $CDCl_3$

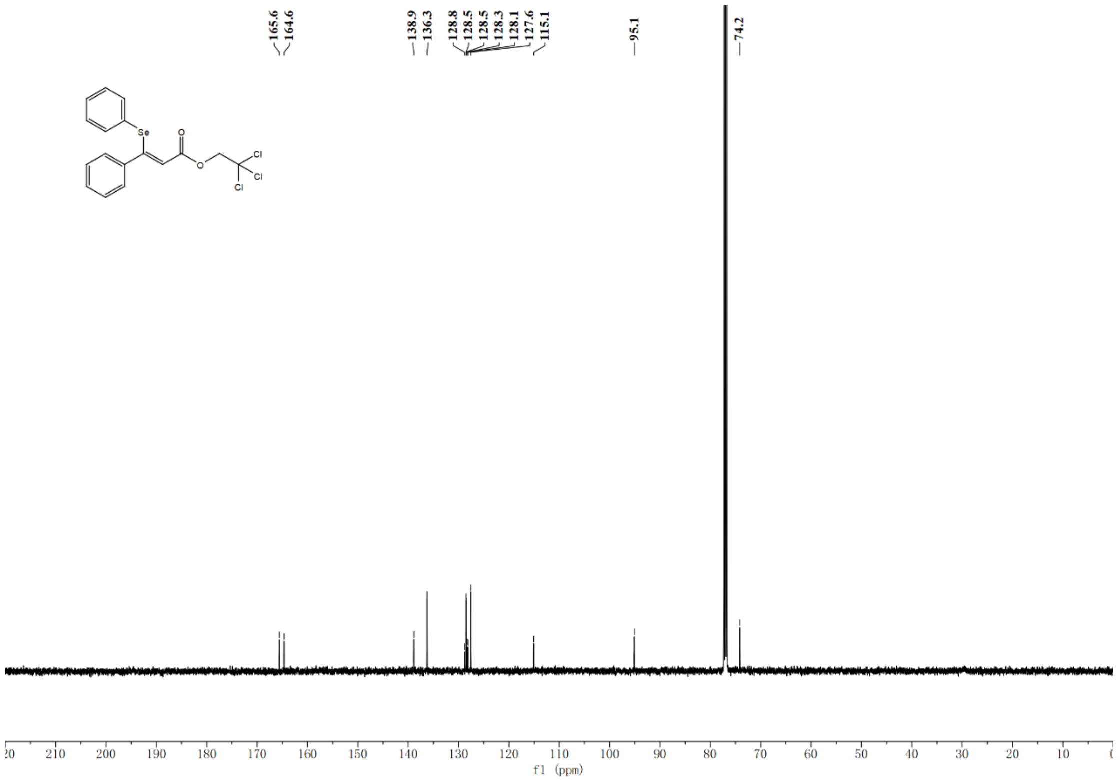

^{1}H NMR (500 MHz) Spectrum of **4aan** in $CDCl_3$

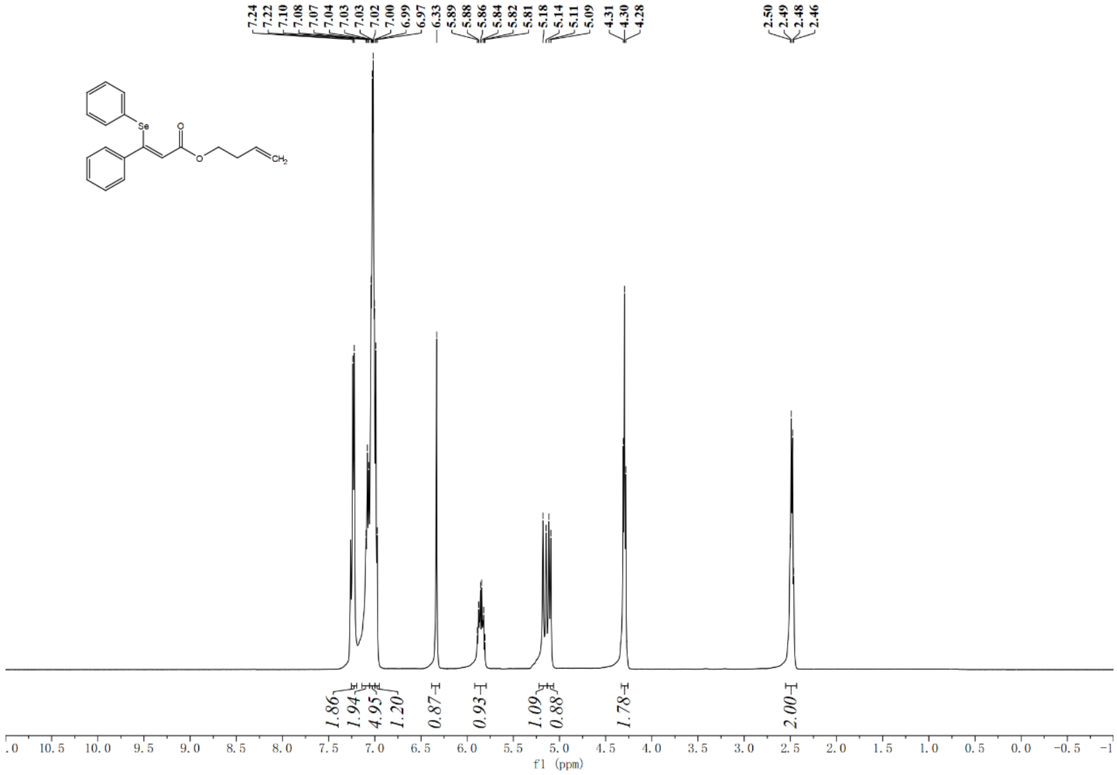

$^{13}C\{^{1}H\}$ NMR (126 MHz) Spectrum of **4aan** in $CDCl_3$

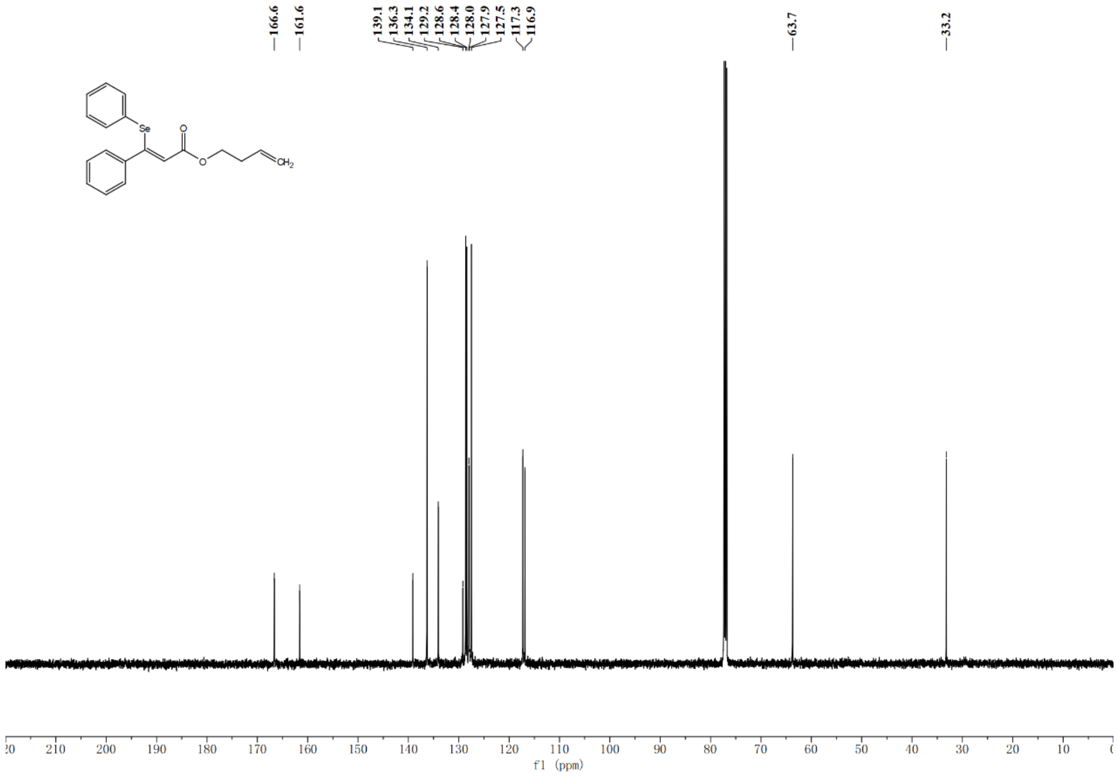

^{1}H NMR (500 MHz) Spectrum of **4aao** in $CDCl_3$

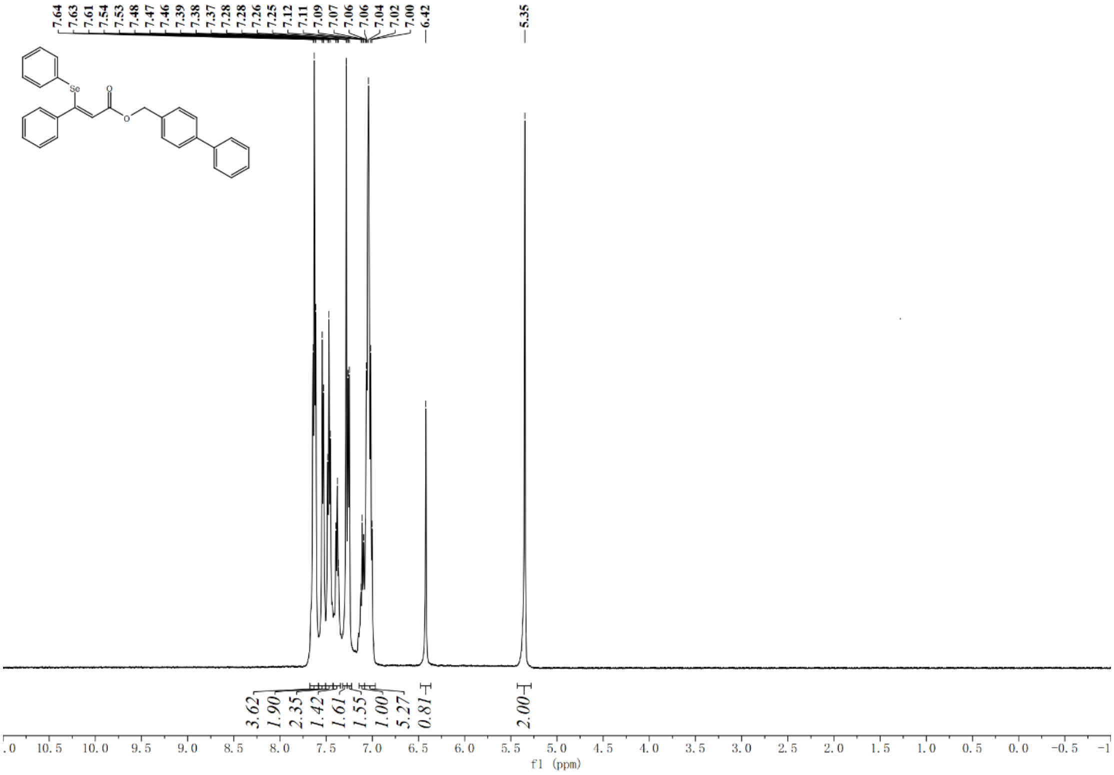

^{13}C{^{1}H} NMR (126 MHz) Spectrum of **4aao** in $CDCl_3$

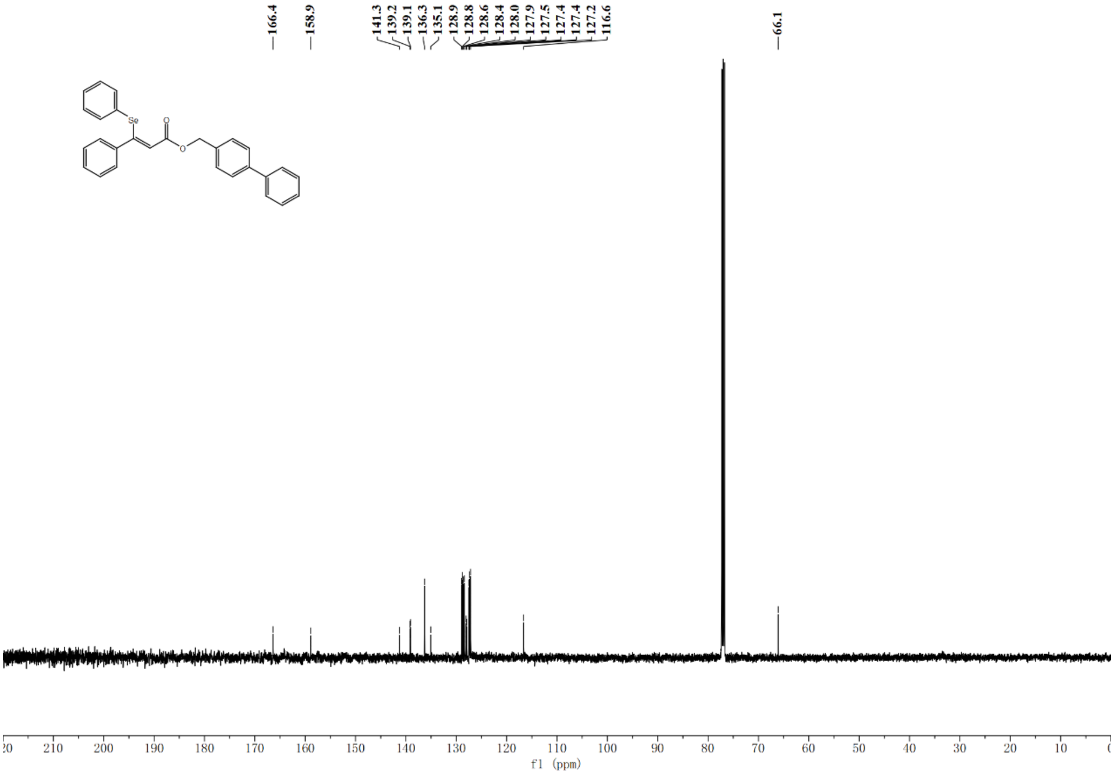

^{1}H NMR (500 MHz) Spectrum of **4aap** in $CDCl_3$

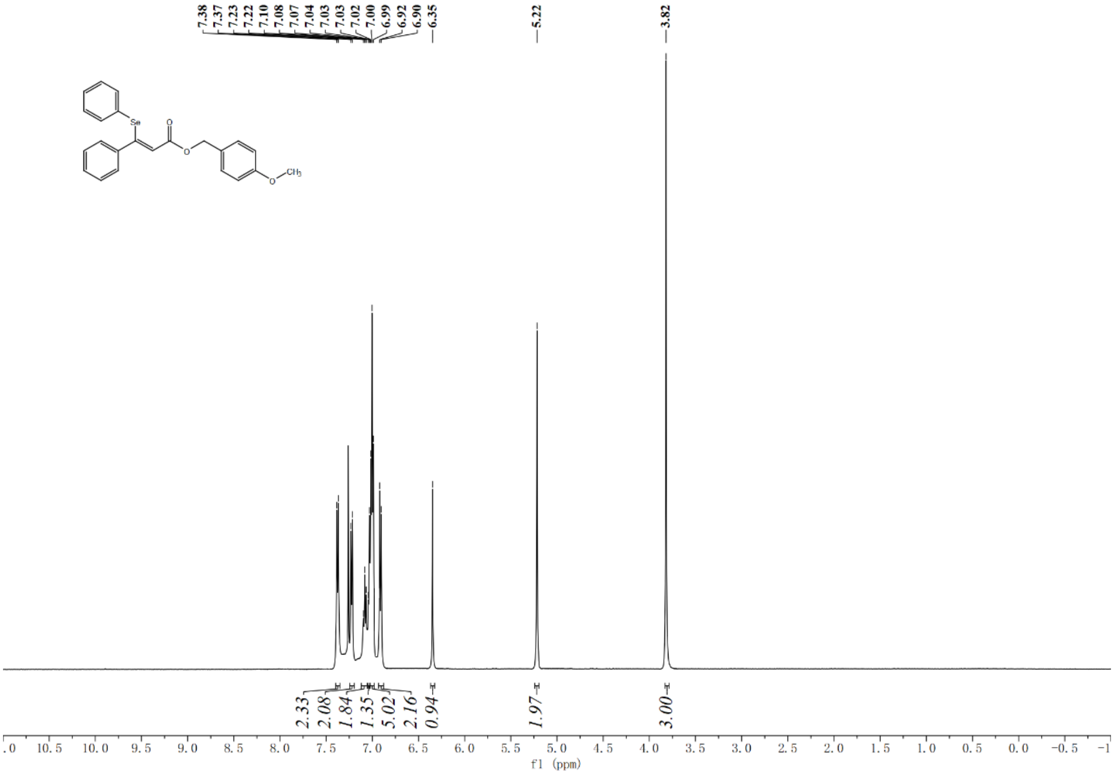

$^{13}C\{^1H\}$ NMR (126 MHz) Spectrum of **4aap** in $CDCl_3$

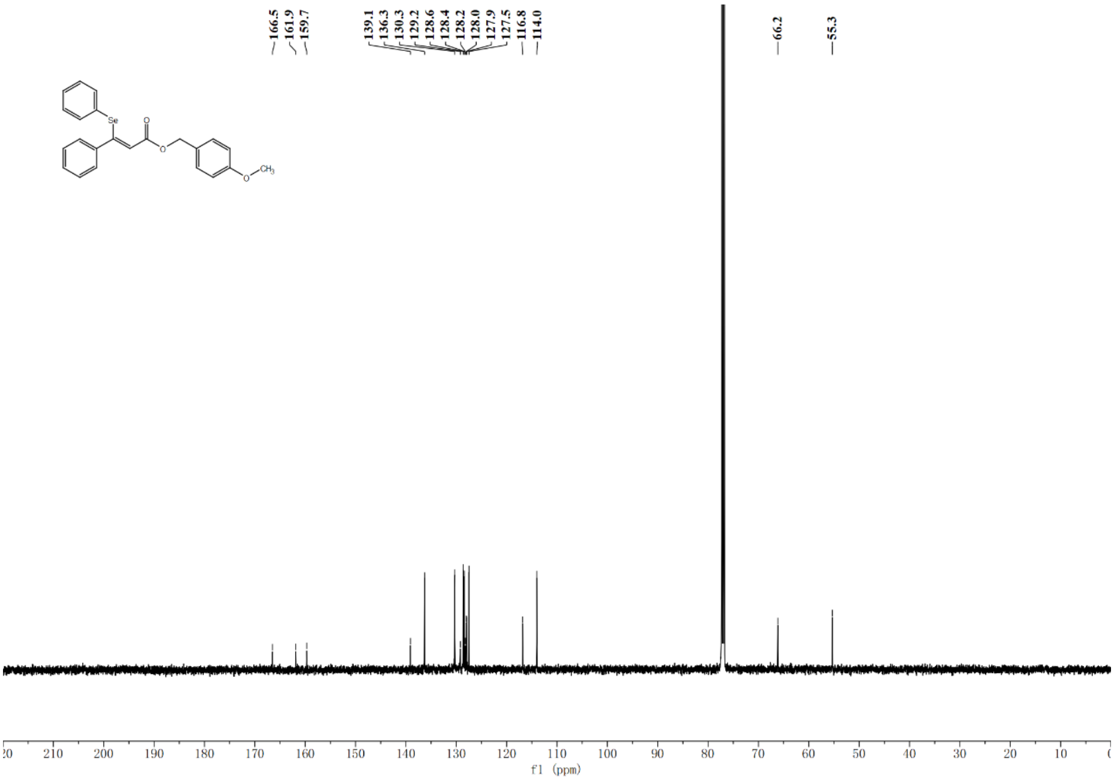

^{1}H NMR (500 MHz) Spectrum of **4aba** in $CDCl_3$

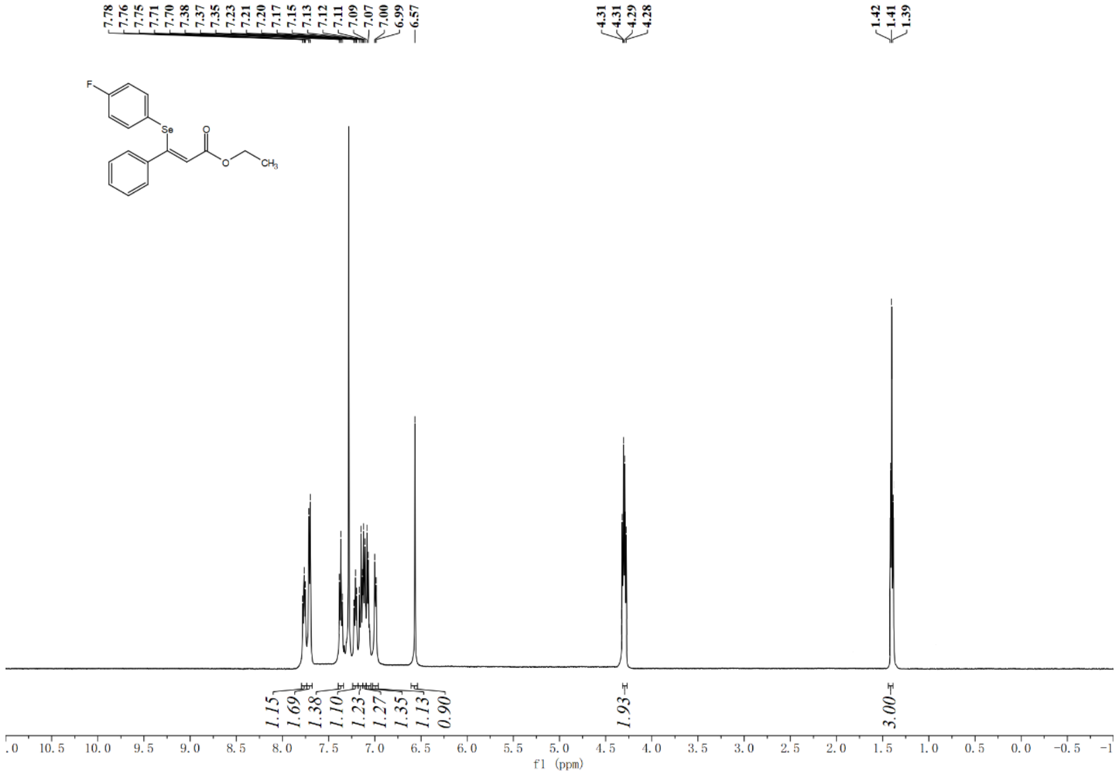

^{13}C{^{1}H} NMR (126 MHz) Spectrum of **4aba** in $CDCl_3$

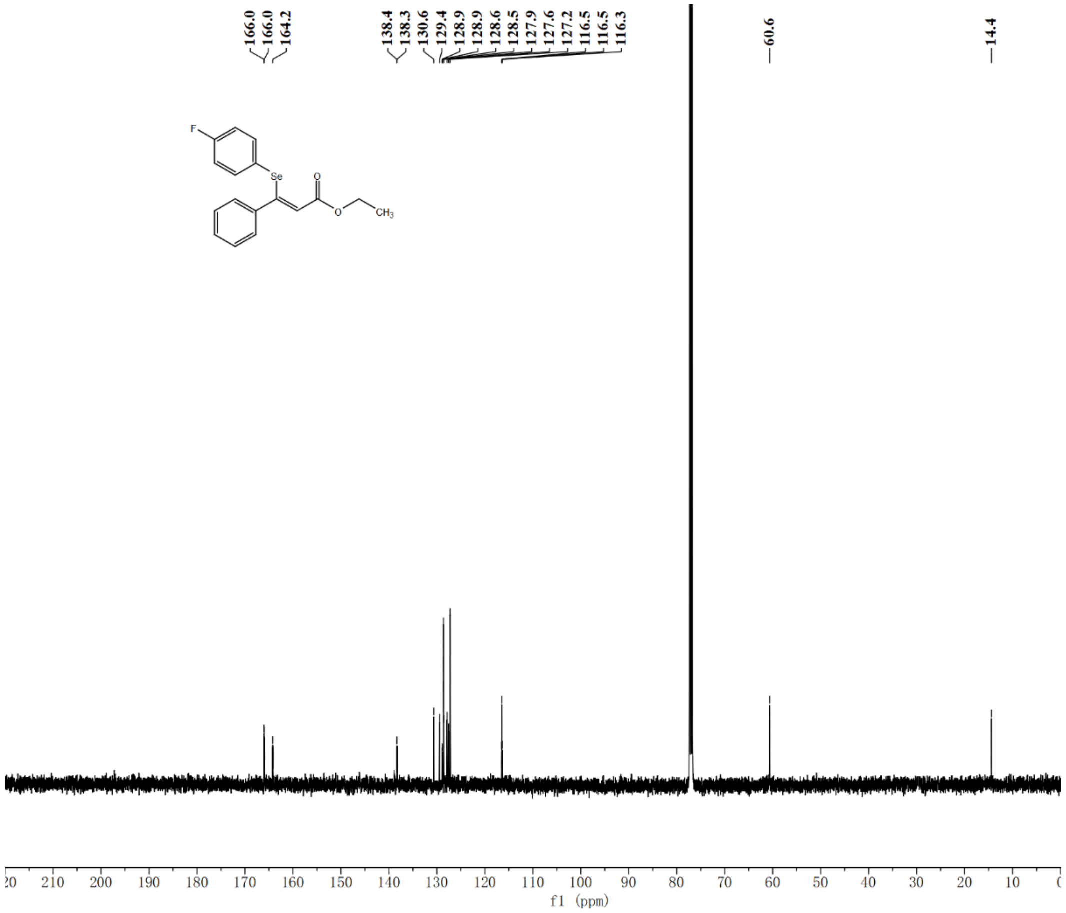

^{19}F NMR (471 MHz) Spectrum of **4aba** in $CDCl_3$

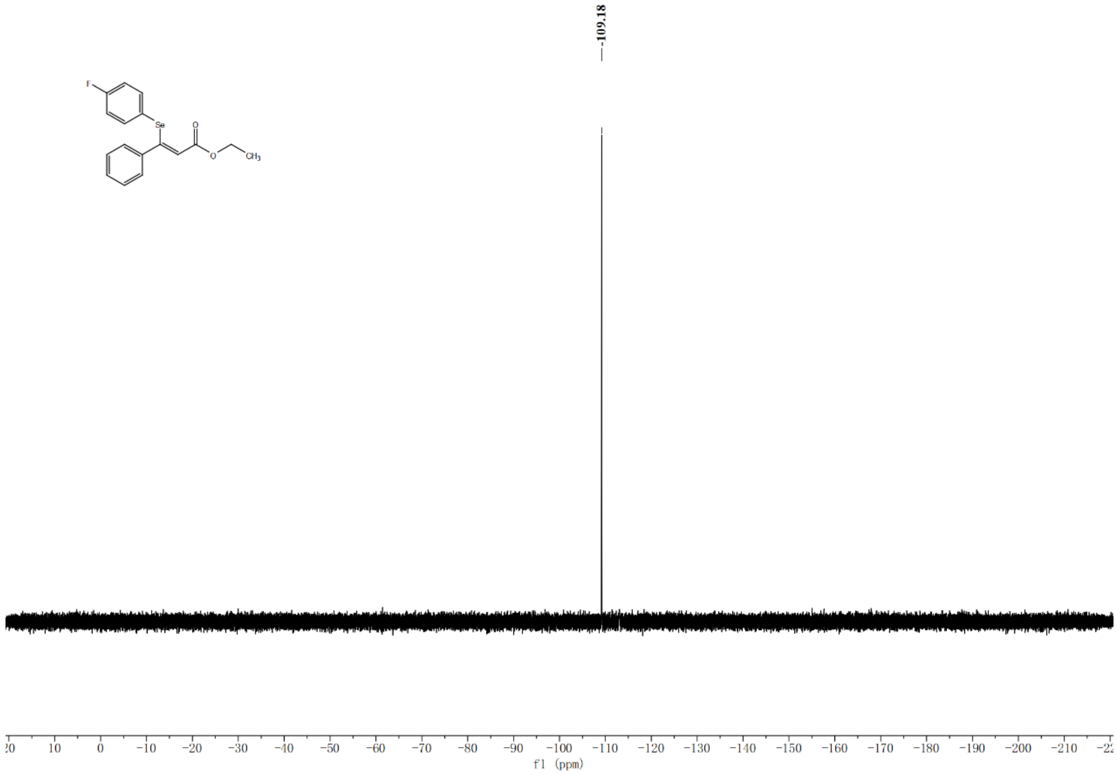

^{1}H NMR (500 MHz) Spectrum of **4aca** in $CDCl_3$

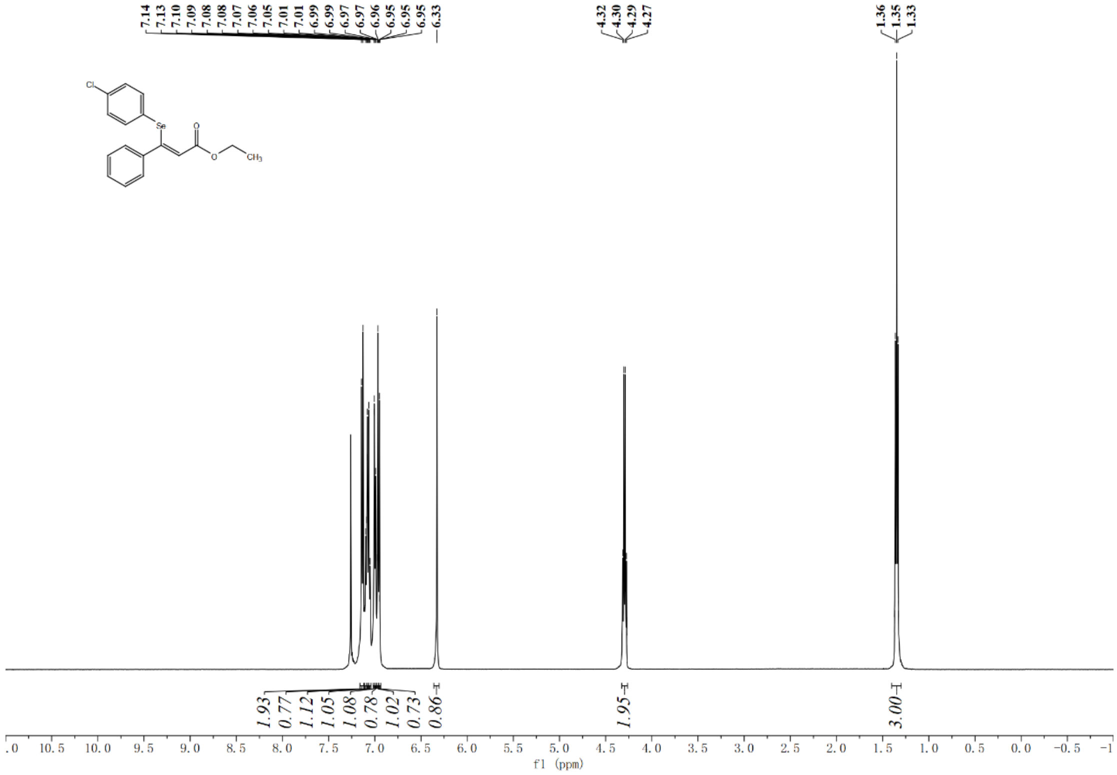

$^{13}C\{^{1}H\}$ NMR (126 MHz) Spectrum of **4aca** in $CDCl_3$

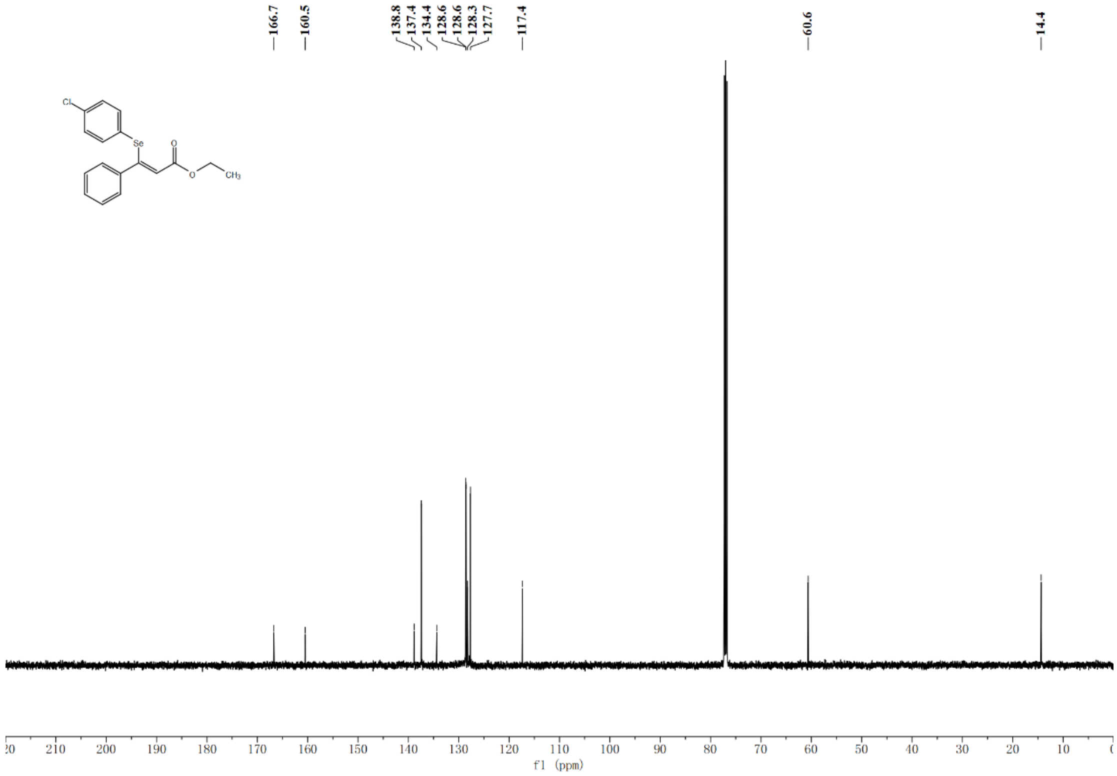

^{1}H NMR (500 MHz) Spectrum of **4baa** in $CDCl_3$

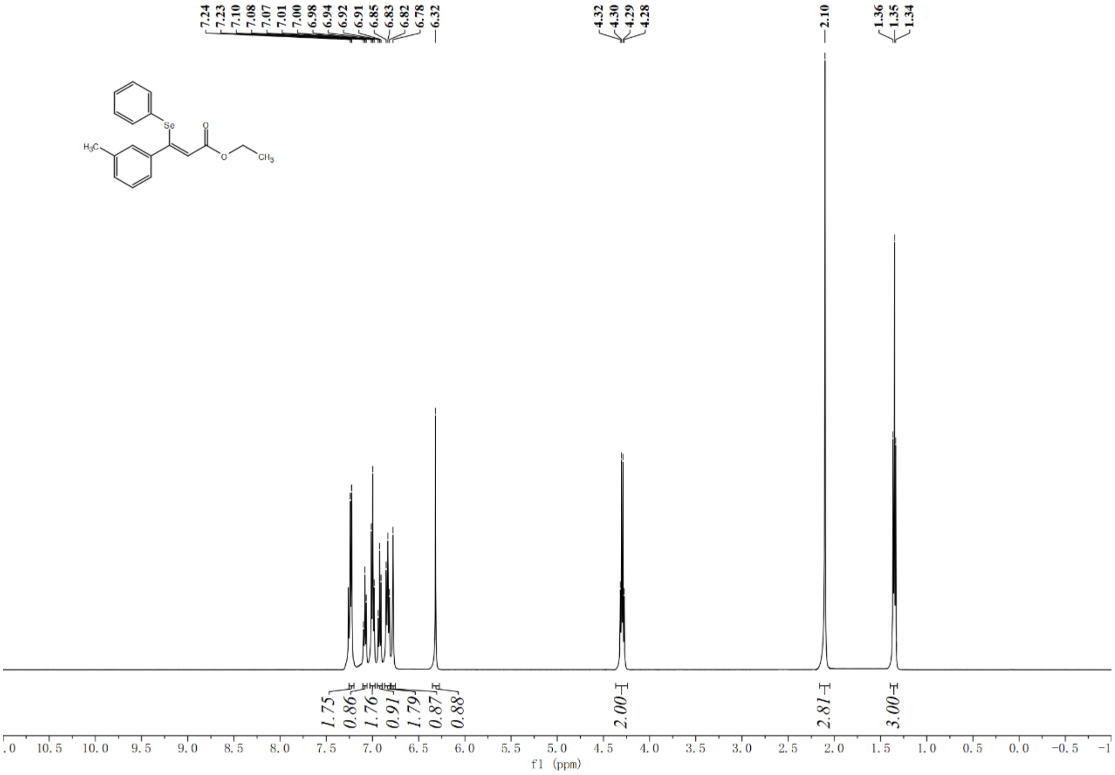

$^{13}C\{^{1}H\}$ NMR (126 MHz) Spectrum of **4baa** in $CDCl_3$

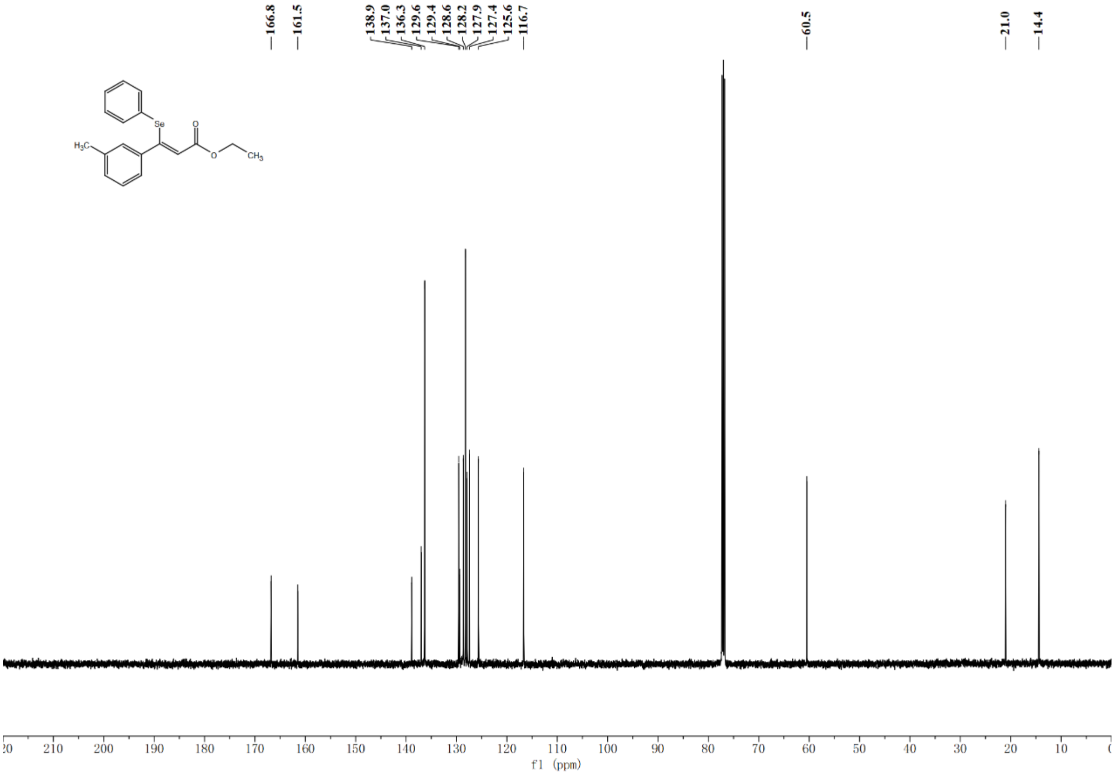

^{1}H NMR (500 MHz) Spectrum of **4caa** in $CDCl_3$

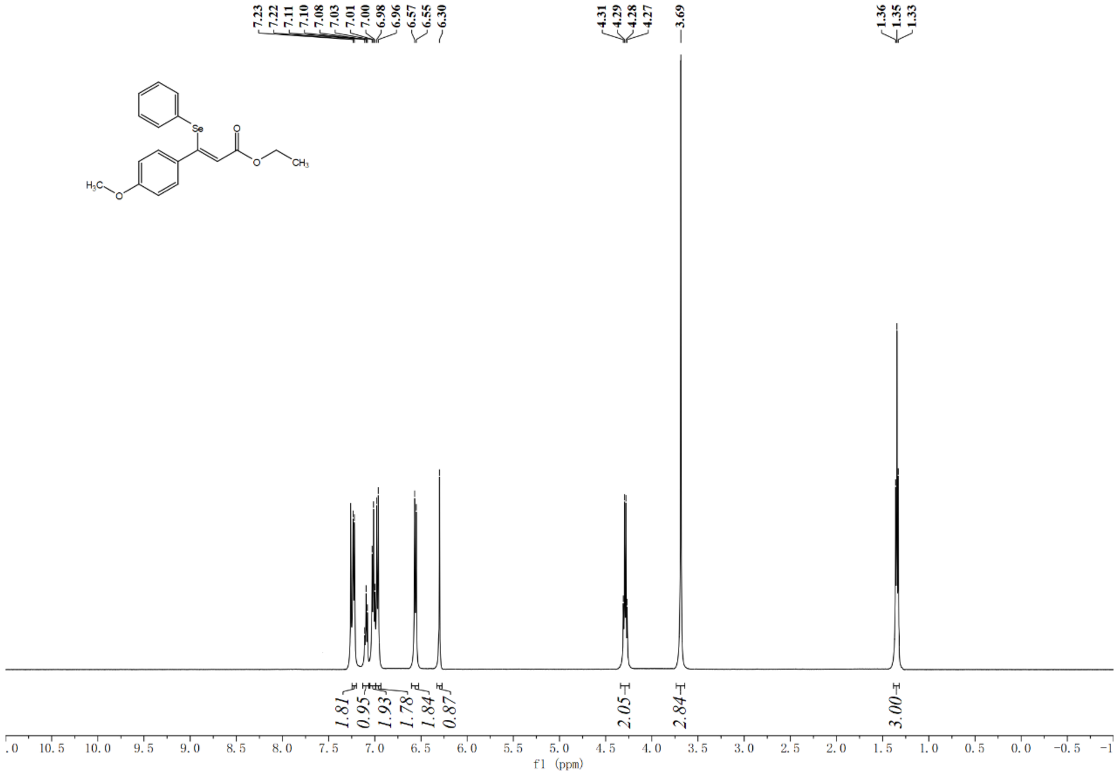

$^{13}C\{^{1}H\}$ NMR (126 MHz) Spectrum of **4caa** in $CDCl_3$

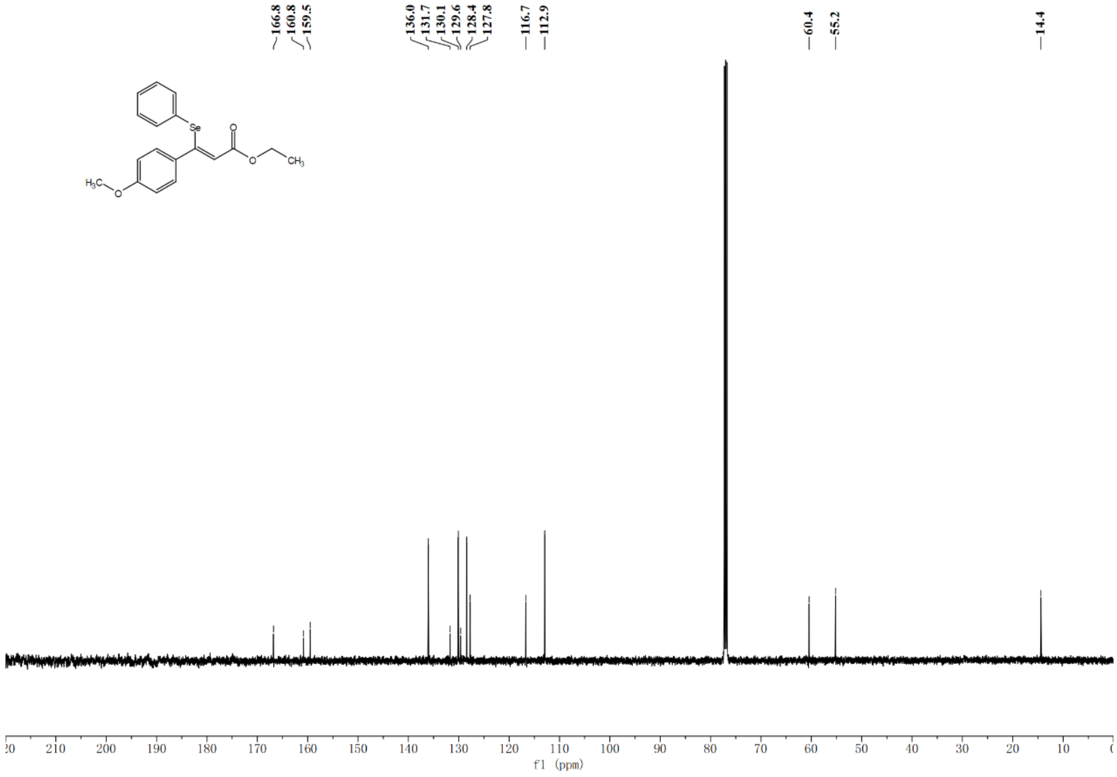

^{1}H NMR (500 MHz) Spectrum of **4daa** in $CDCl_3$

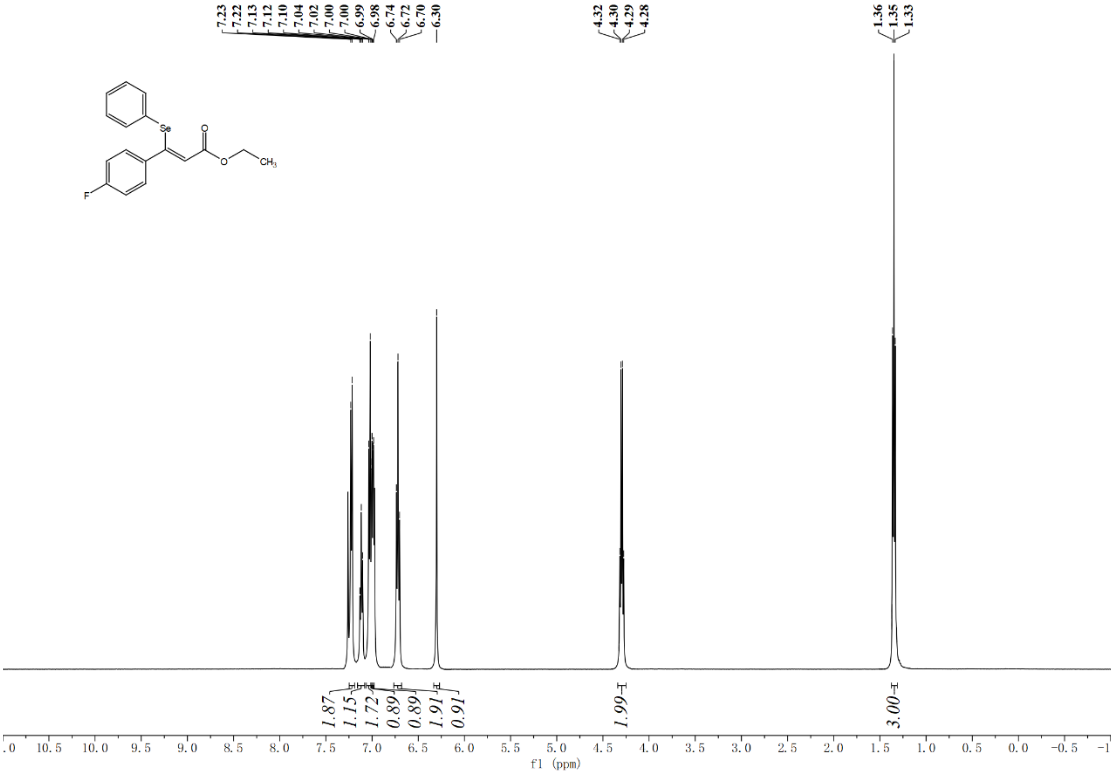

^{13}C{^{1}H} NMR (126 MHz) Spectrum of **4daa** in $CDCl_3$

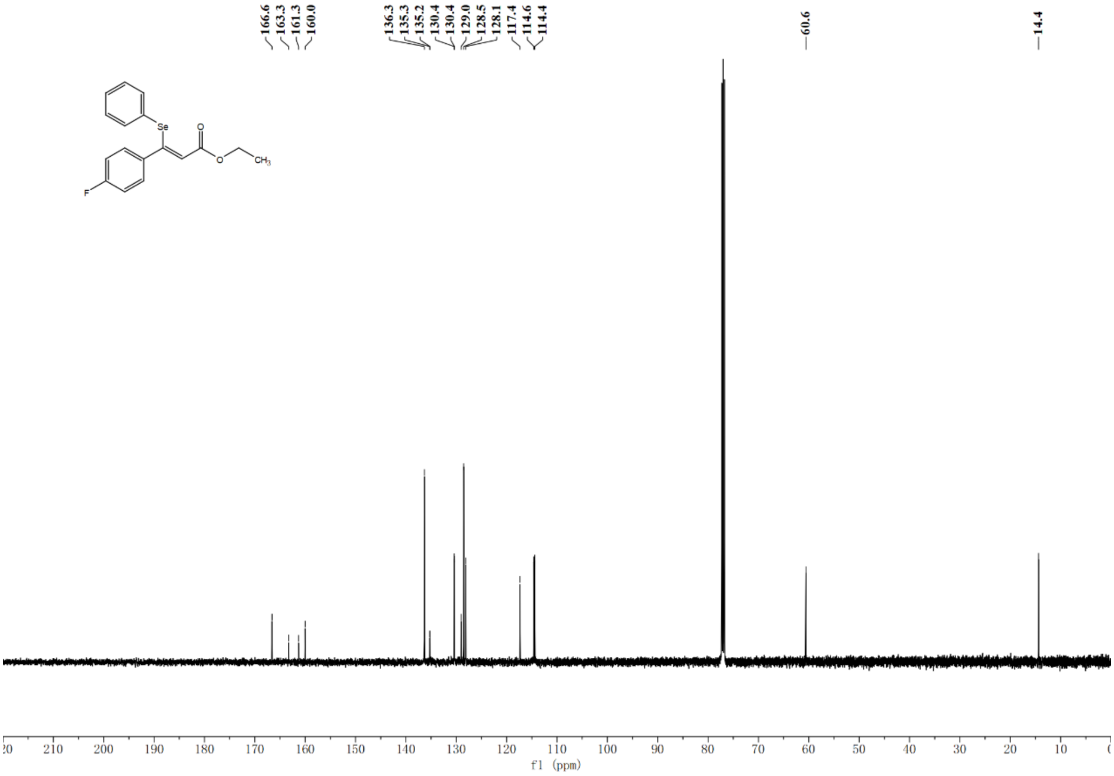

^{19}F NMR (471 MHz) Spectrum of **4daa** in $CDCl_3$

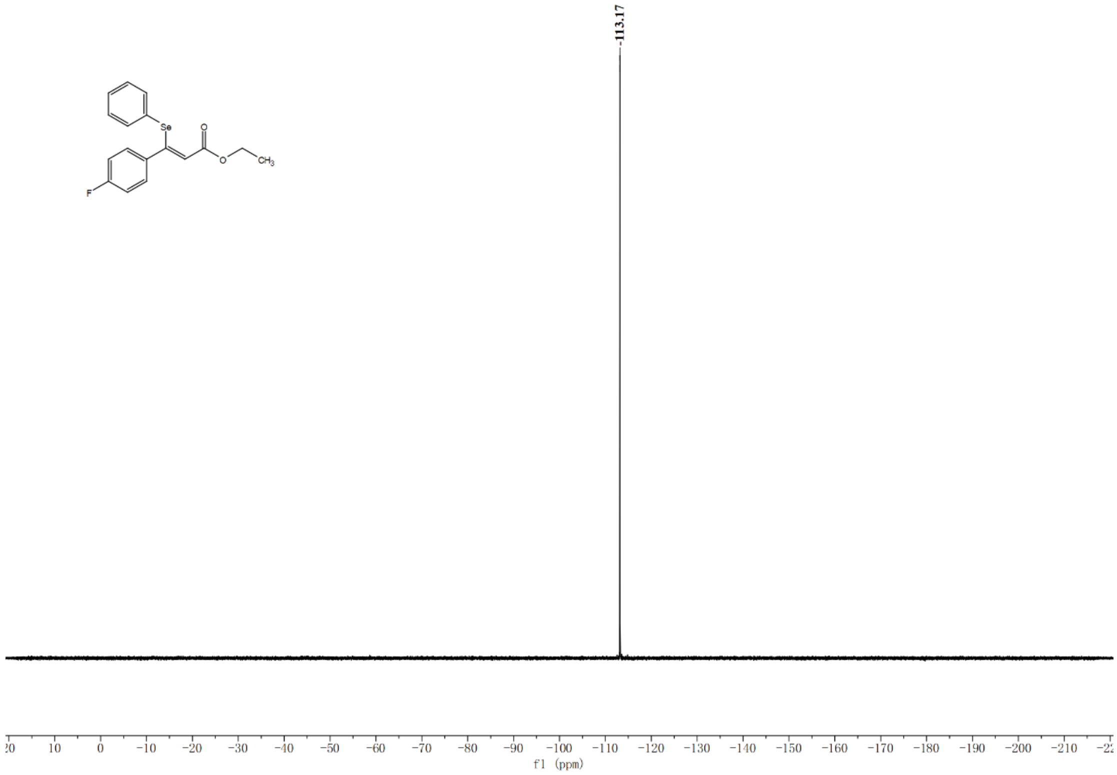

^{1}H NMR (500 MHz) Spectrum of **4eaa** in $CDCl_3$

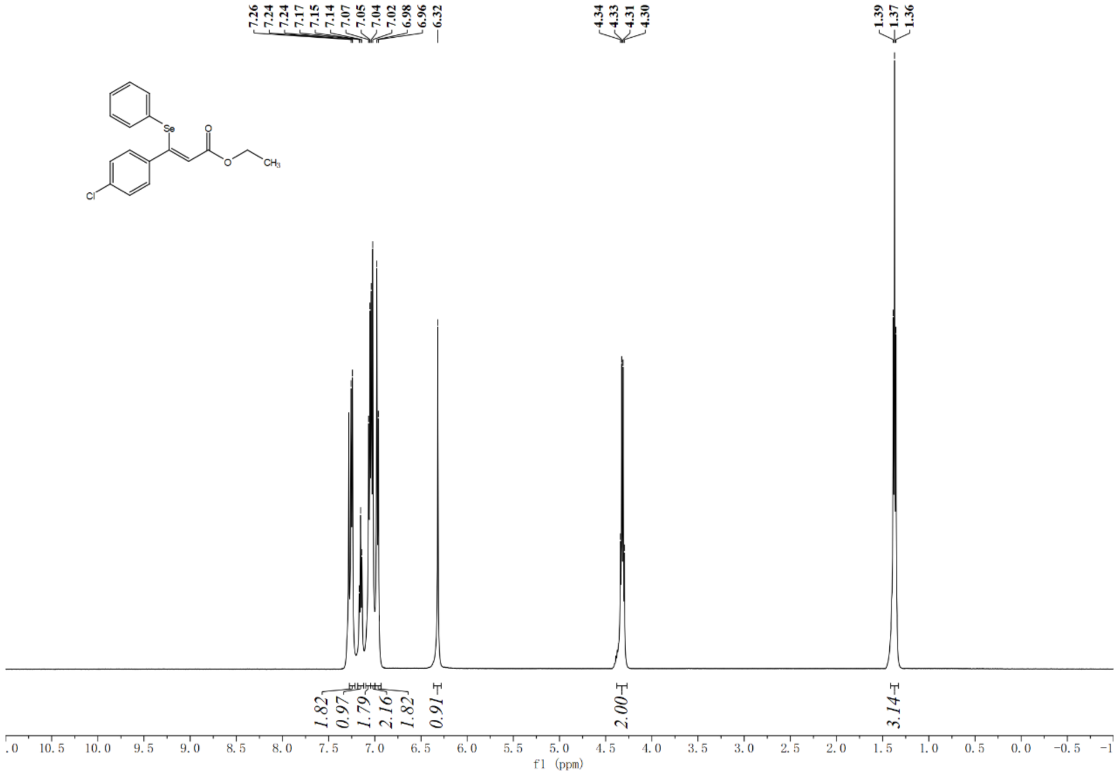

^{13}C{^{1}H} NMR (126 MHz) Spectrum of **4eaa** in $CDCl_3$

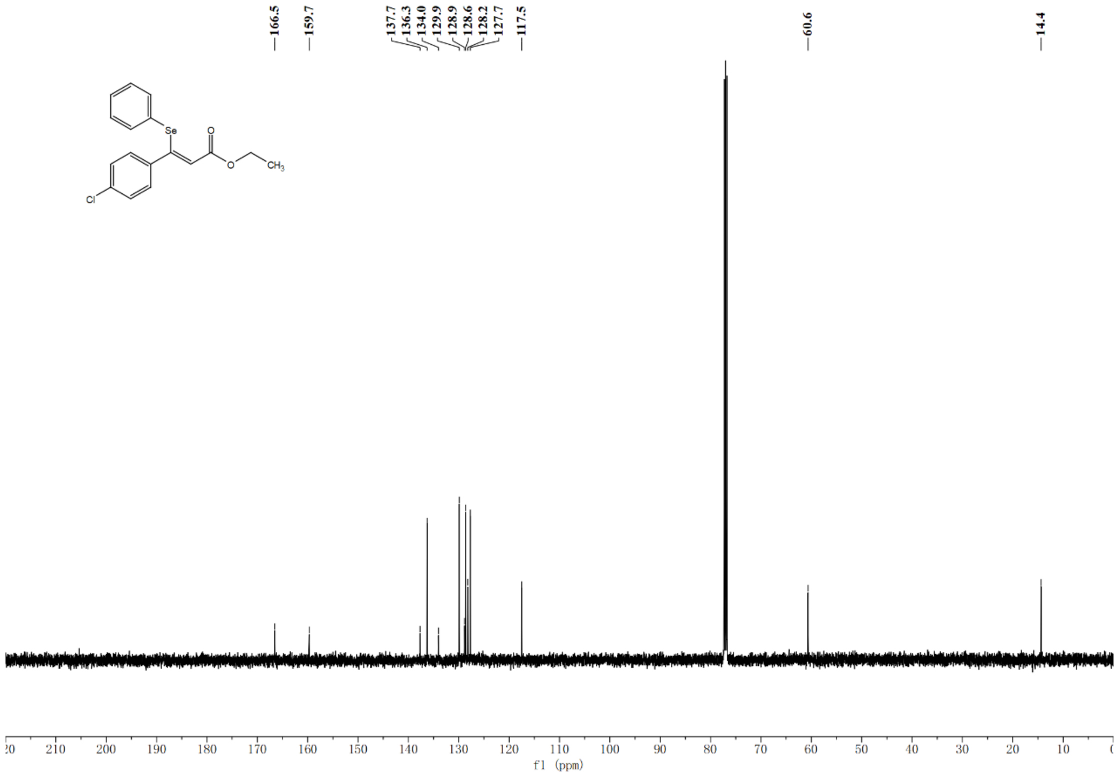

^{1}H NMR (500 MHz) Spectrum of **4faa** in $CDCl_3$

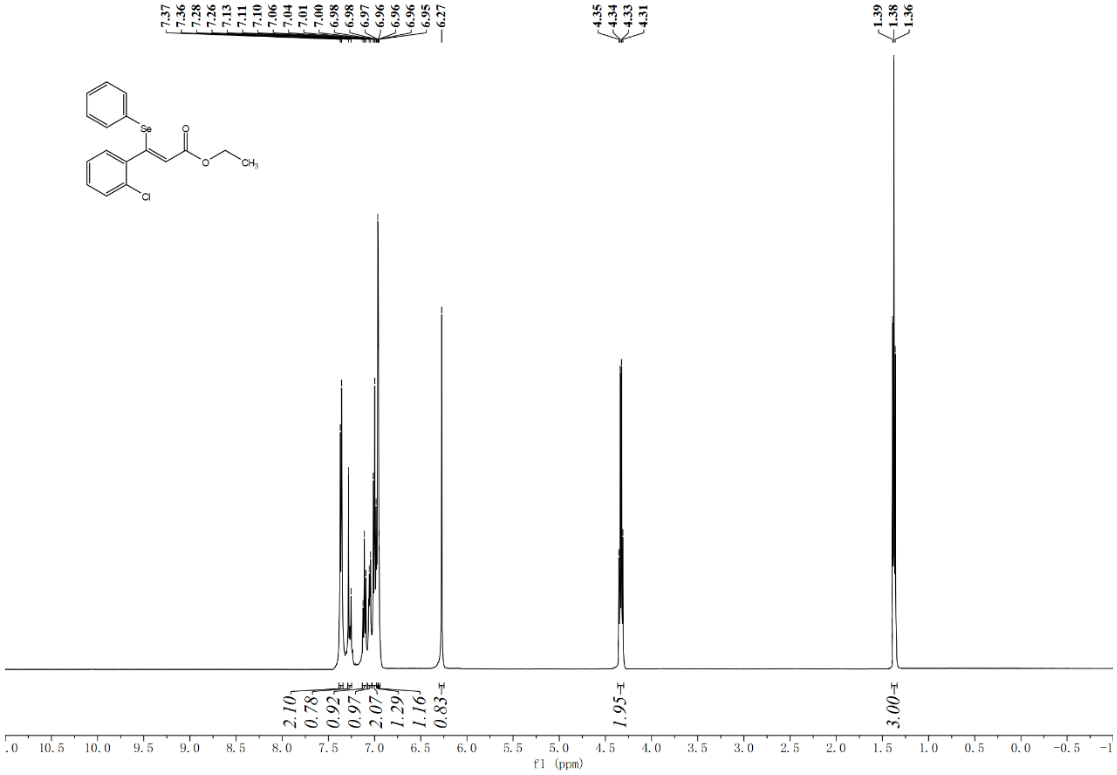

$^{13}C\{^{1}H\}$ NMR (126 MHz) Spectrum of **4faa** in $CDCl_3$

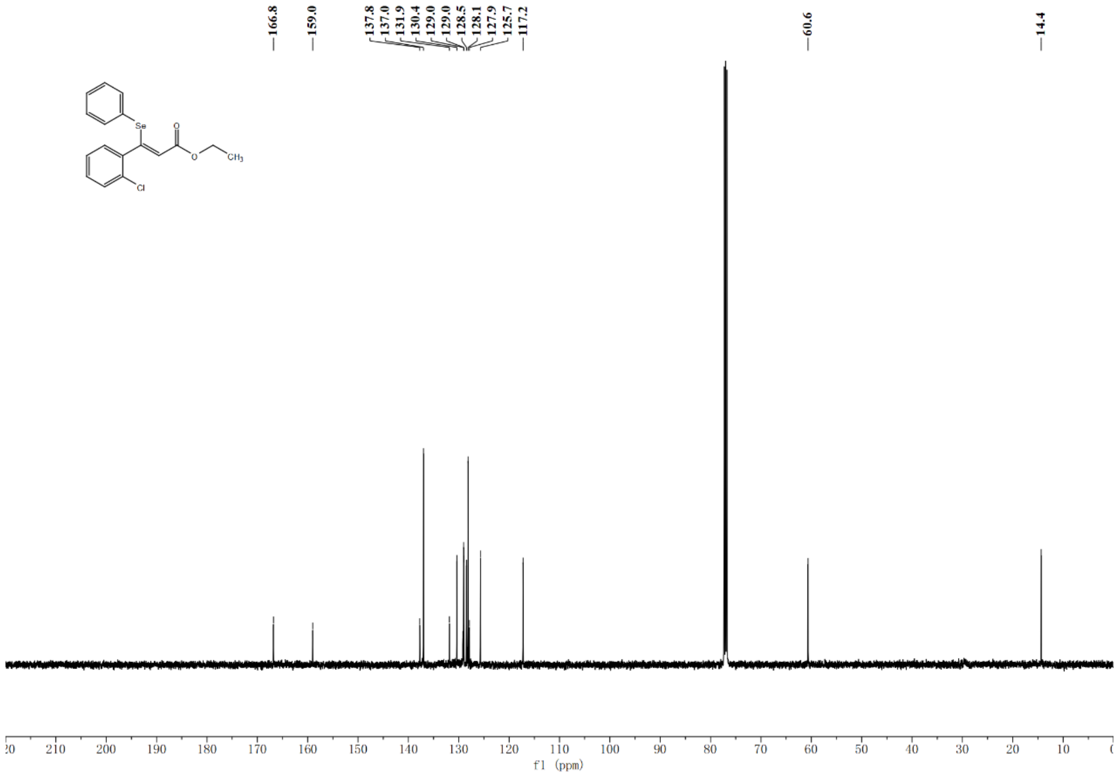

^{1}H NMR (500 MHz) Spectrum of **4gaa** in $CDCl_3$

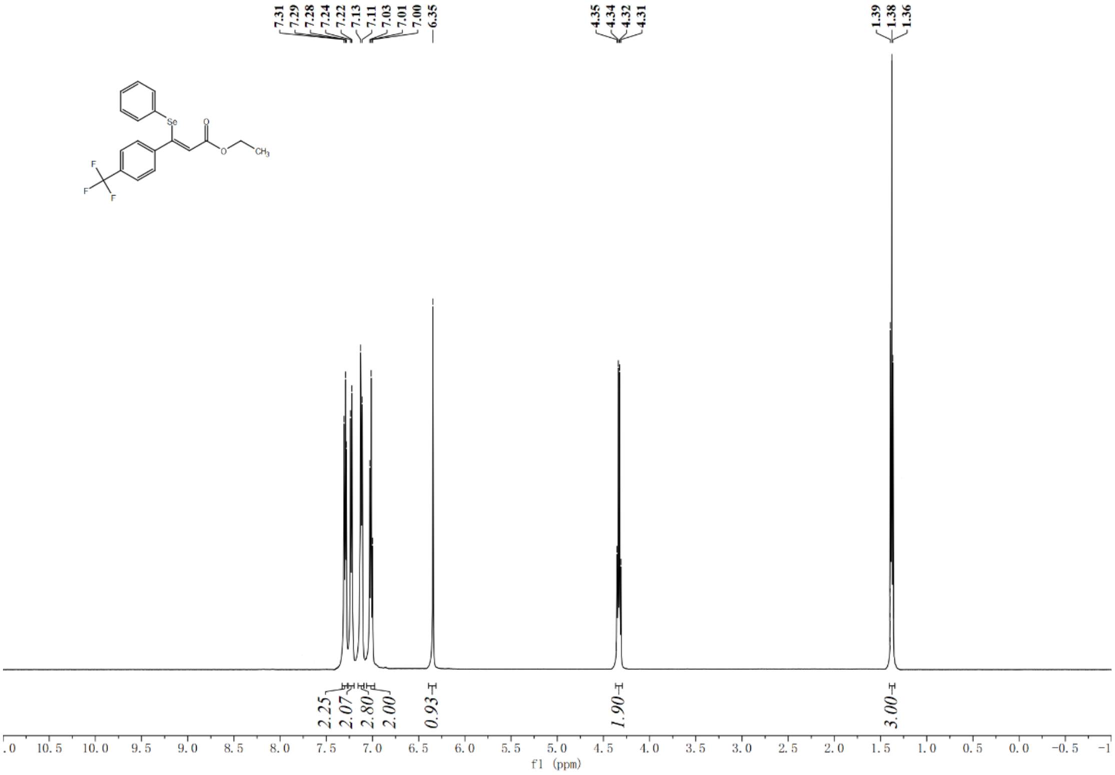

$^{13}C\{^1H\}$ NMR (101 MHz) Spectrum of **4gaa** in $CDCl_3$

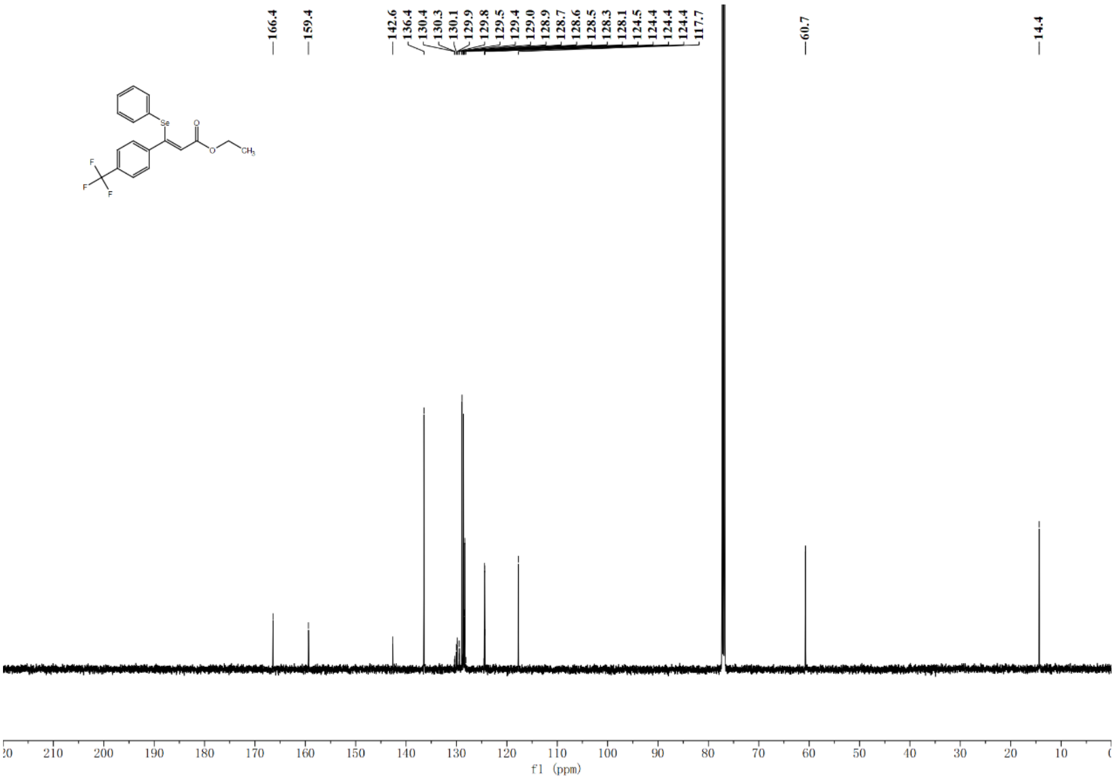

^{19}F NMR (471 MHz) Spectrum of **4gaa** in $CDCl_3$

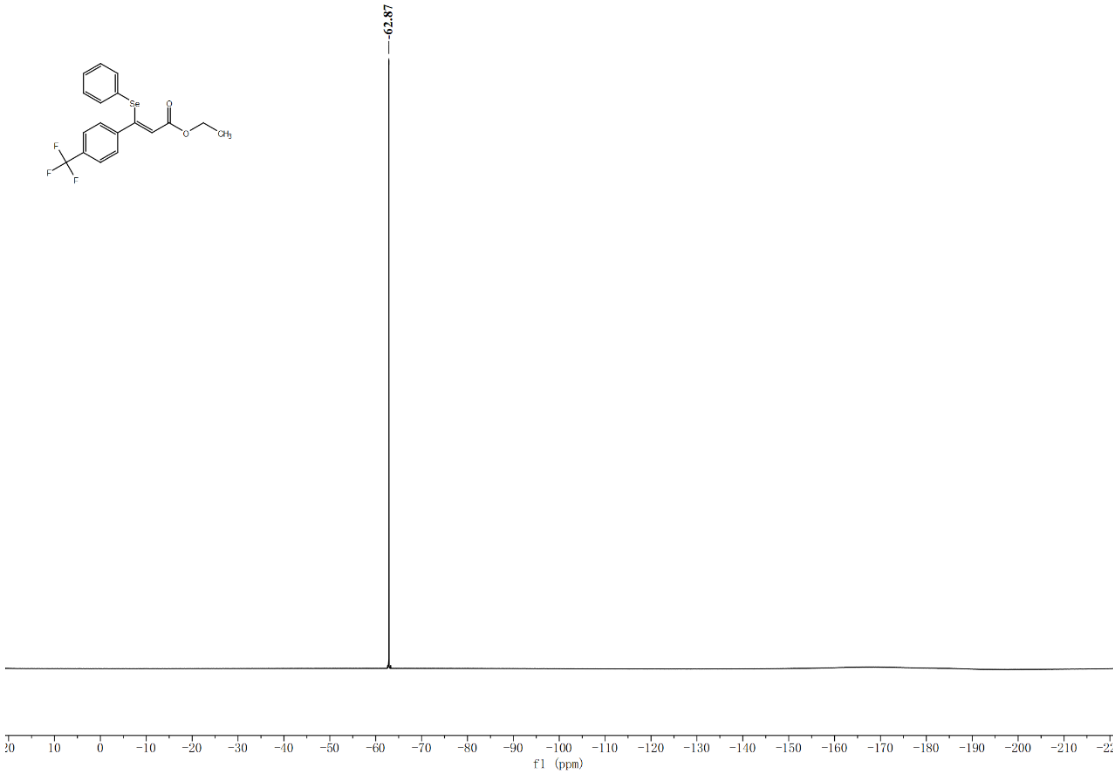

^{1}H NMR (500 MHz) Spectrum of **4haa** in $CDCl_3$

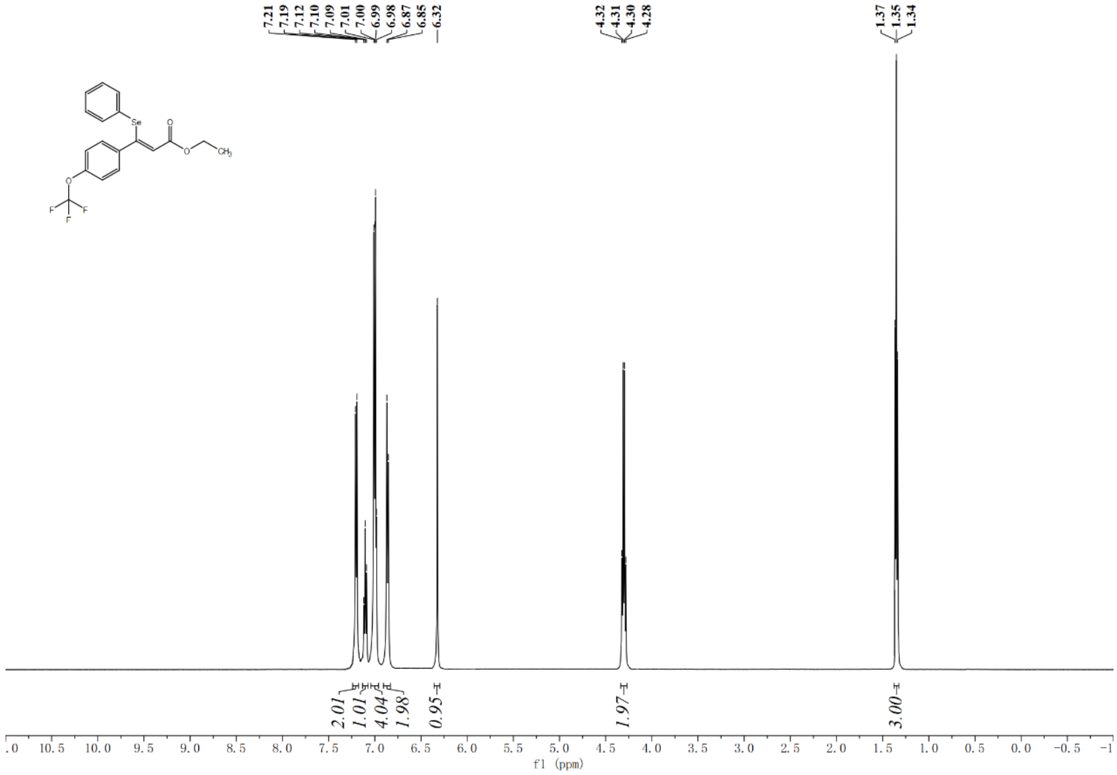

$^{13}C\{^1H\}$ NMR (126 MHz) Spectrum of **4haa** in $CDCl_3$

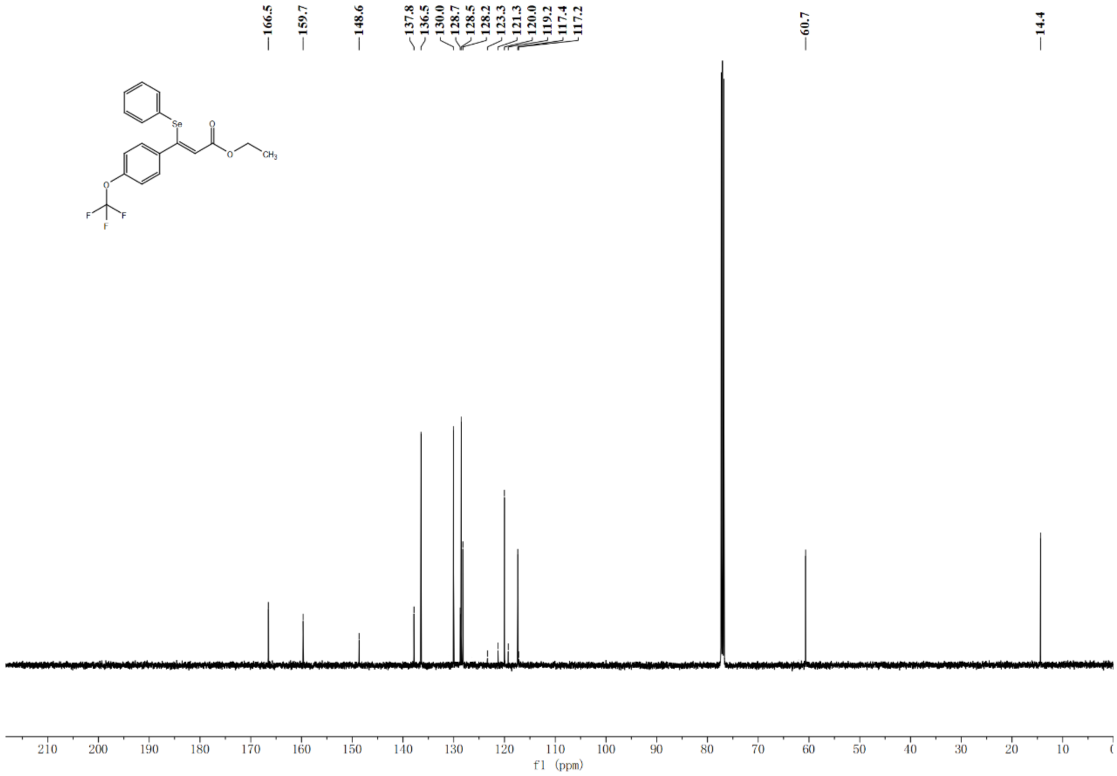

^{19}F NMR (471 MHz) Spectrum of **4haa** in $CDCl_3$

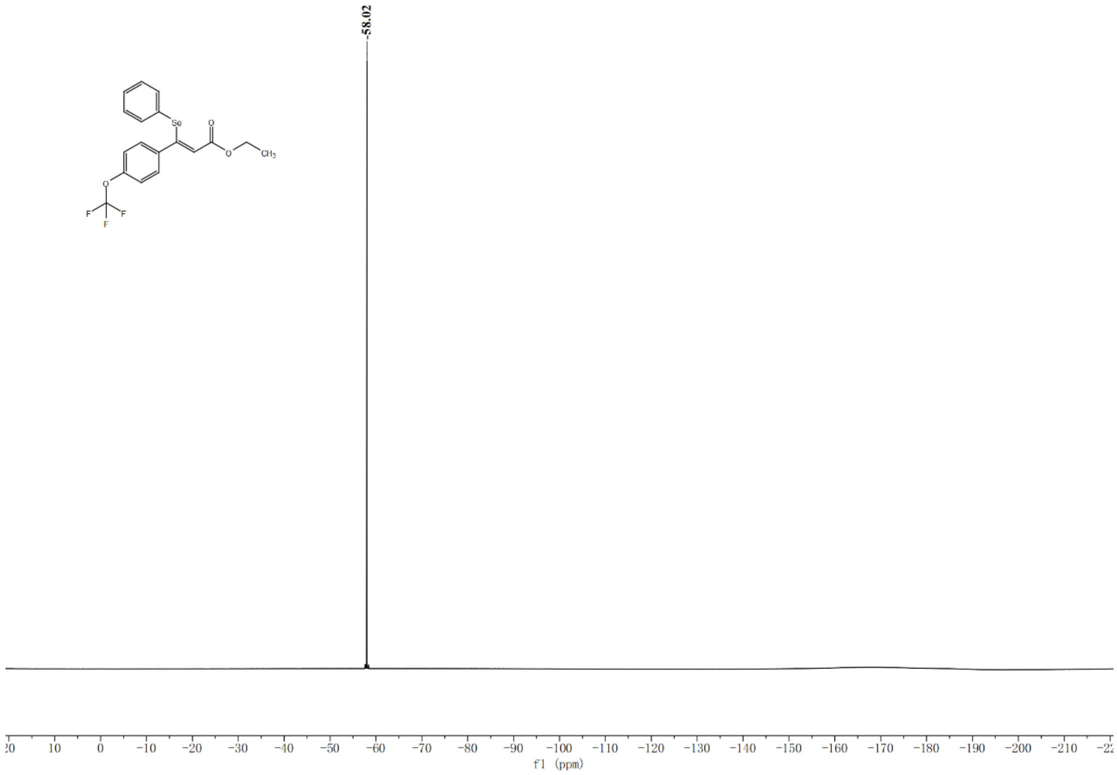

^{1}H NMR (500 MHz) Spectrum of **4iaa** in $CDCl_3$

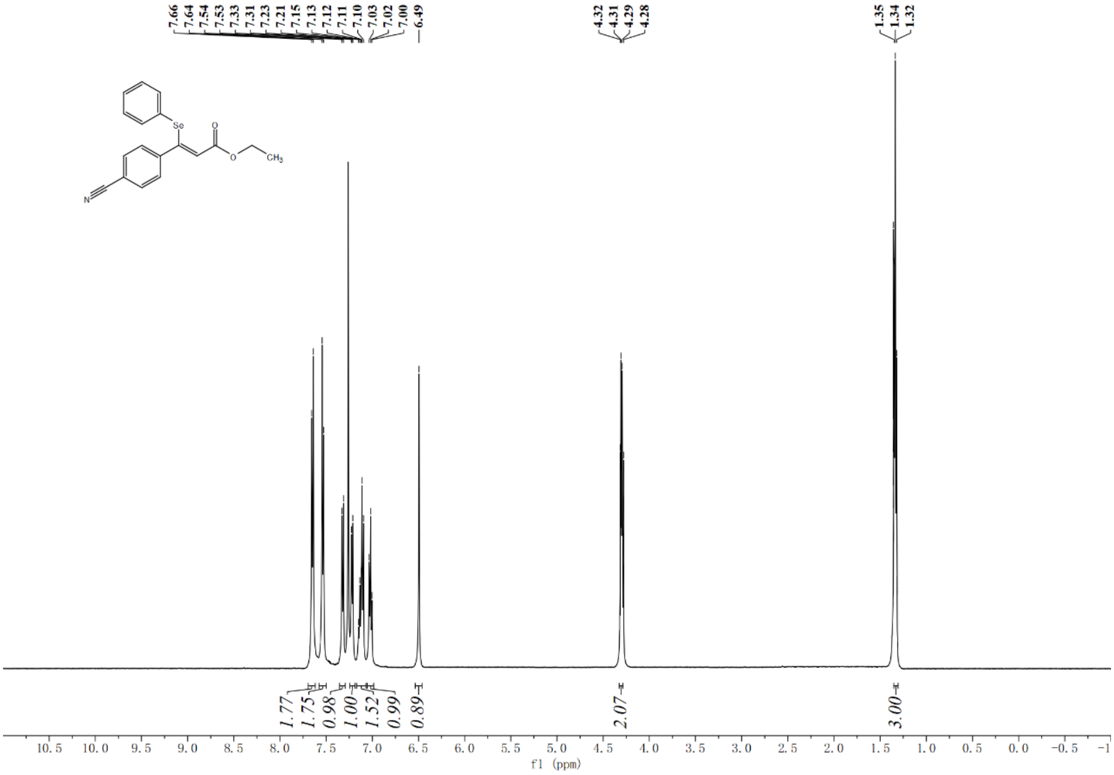

$^{13}C\{^{1}H\}$ NMR (126 MHz) Spectrum of **4iaa** in $CDCl_3$

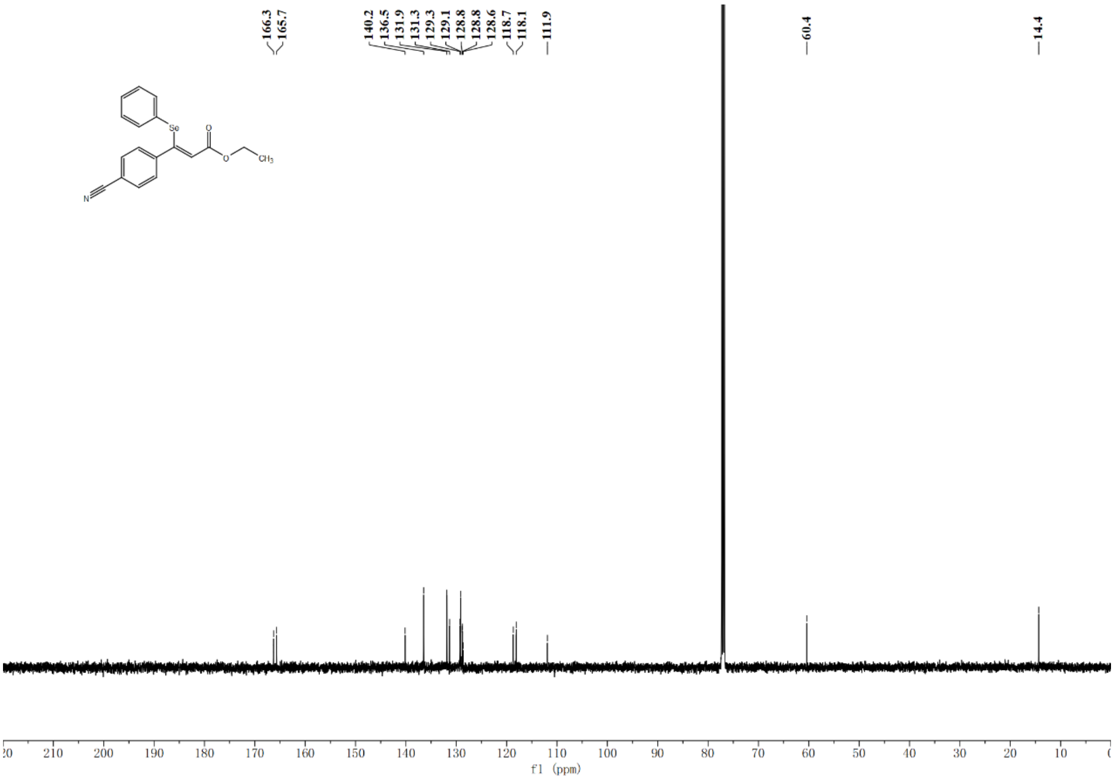

^{1}H NMR (500 MHz) Spectrum of **4jaa** in $CDCl_3$

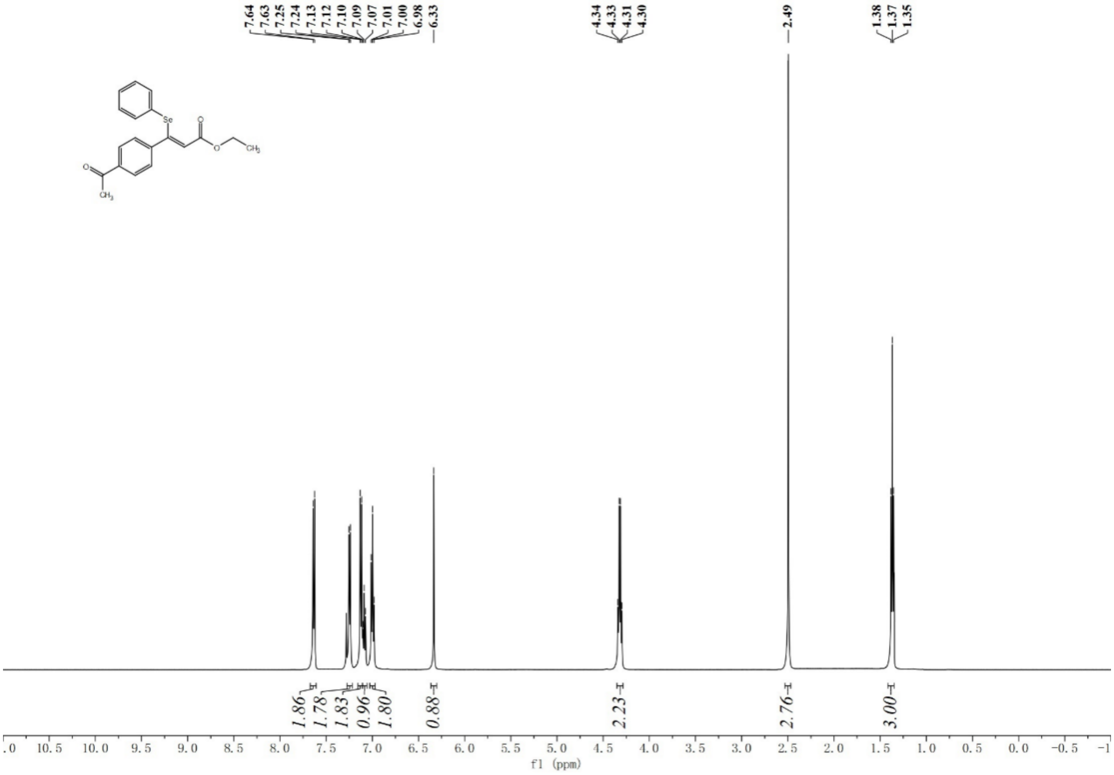

$^{13}C\{^{1}H\}$ NMR (126 MHz) Spectrum of **4jaa** in $CDCl_{3S}$

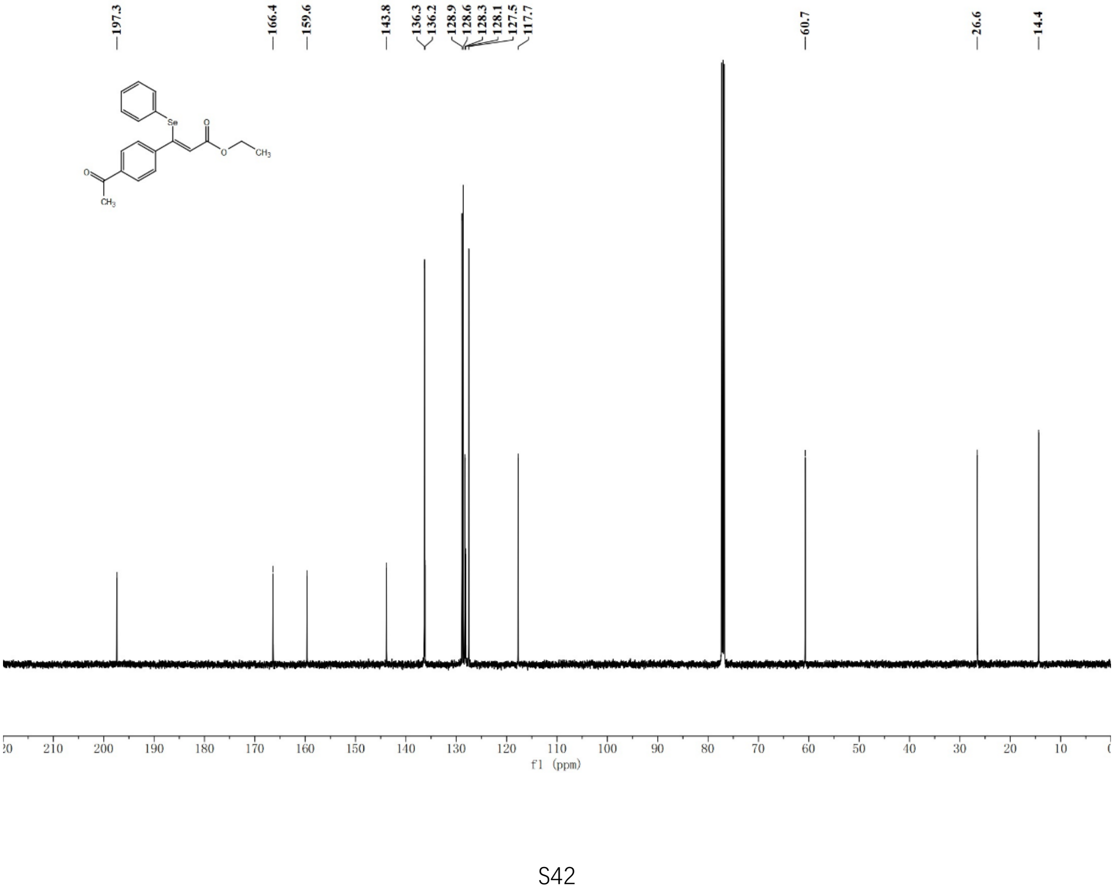

^{1}H NMR (500 MHz) Spectrum of **4kaa** in $CDCl_3$

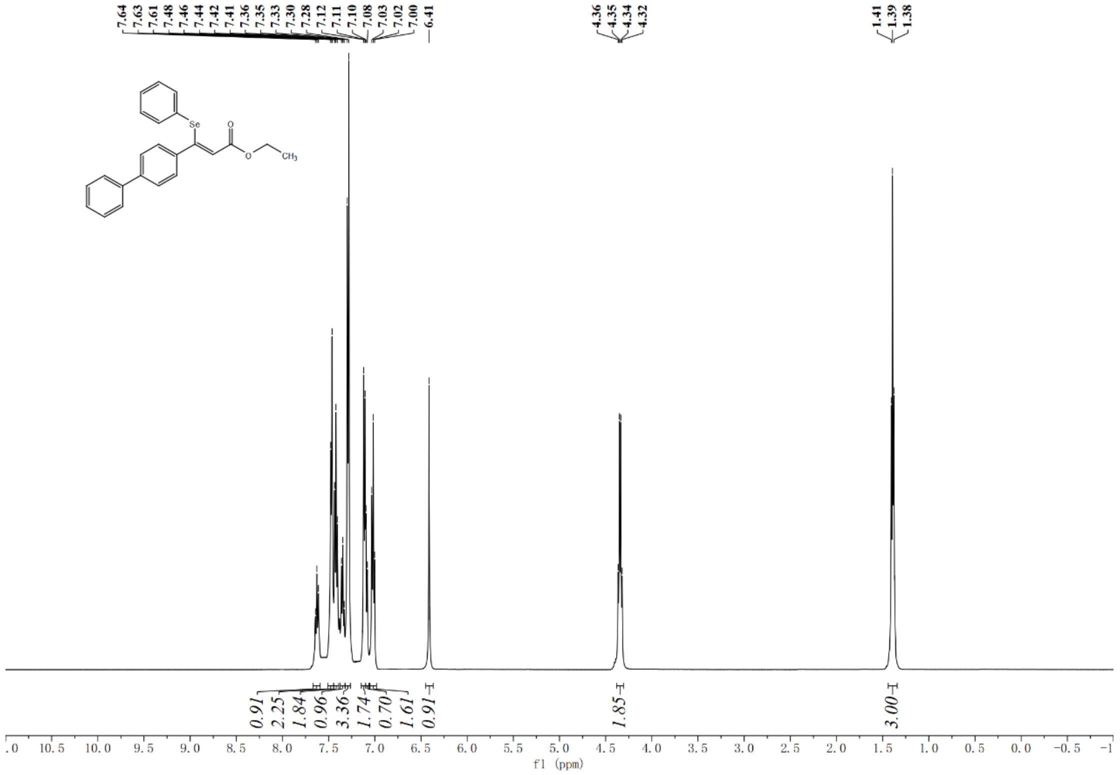

$^{13}C\{^{1}H\}$ NMR (126 MHz) Spectrum of **4kaa** in $CDCl_3$

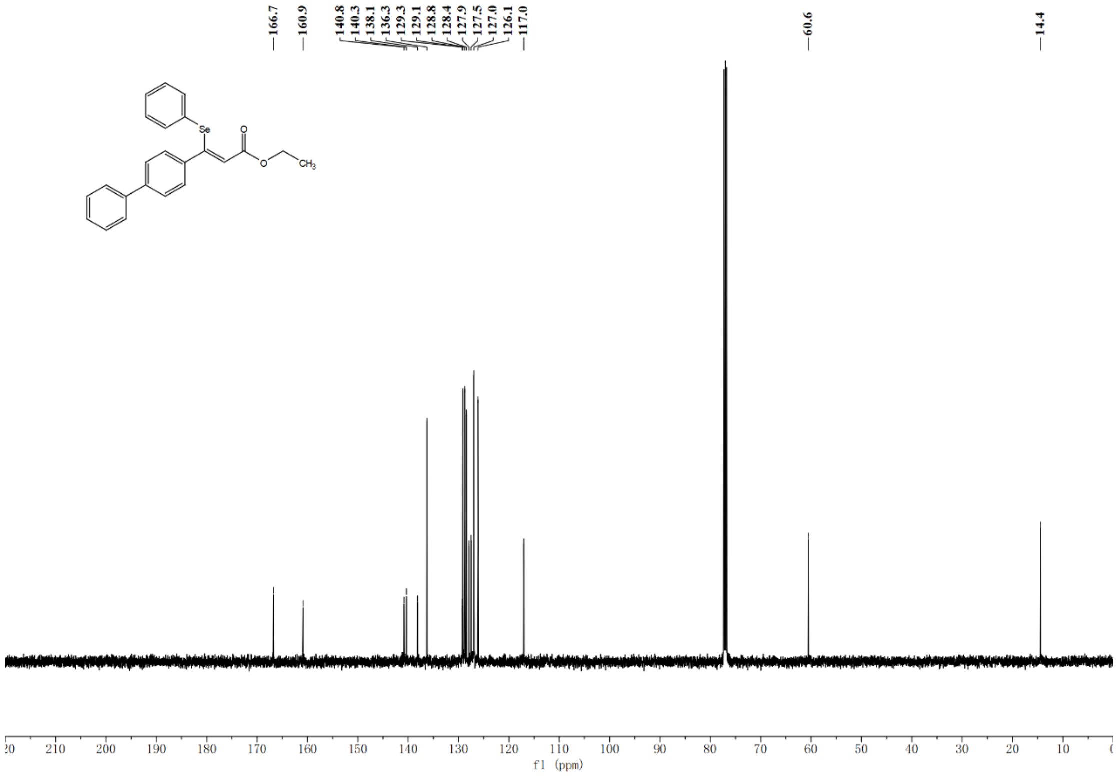

^{1}H NMR (500 MHz) Spectrum of **4laa** in $CDCl_3$

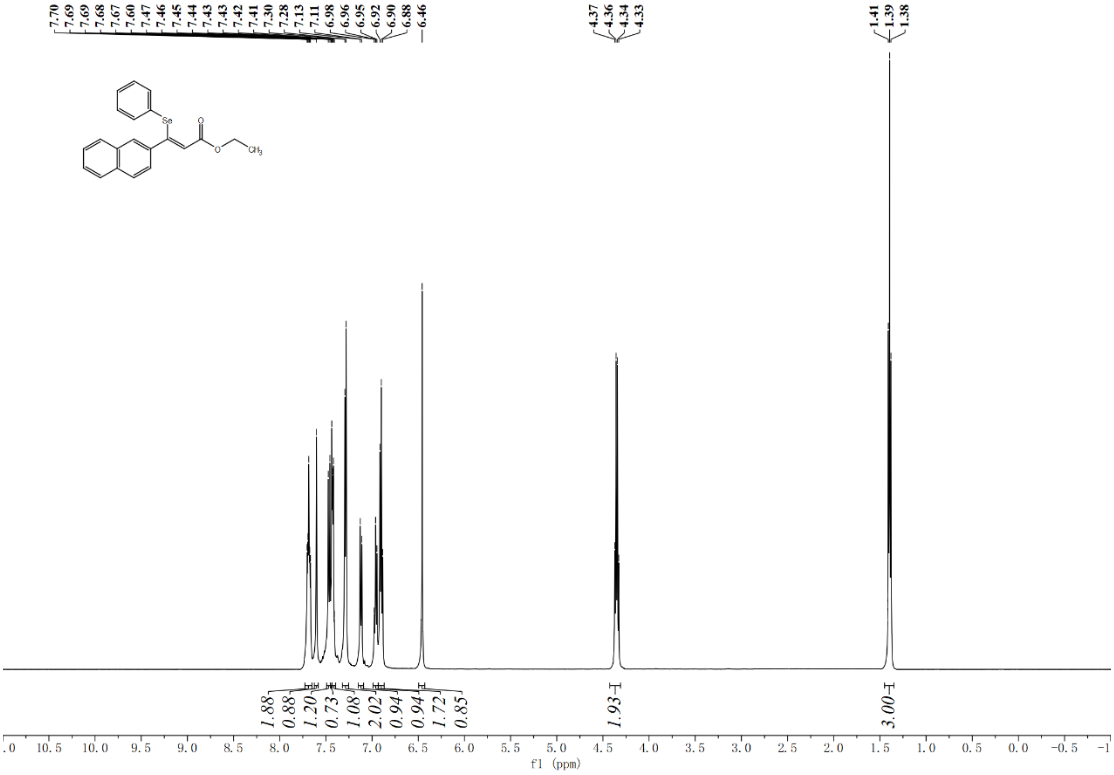

^{13}C{^{1}H} NMR (126 MHz) Spectrum of **4laa** in $CDCl_3$

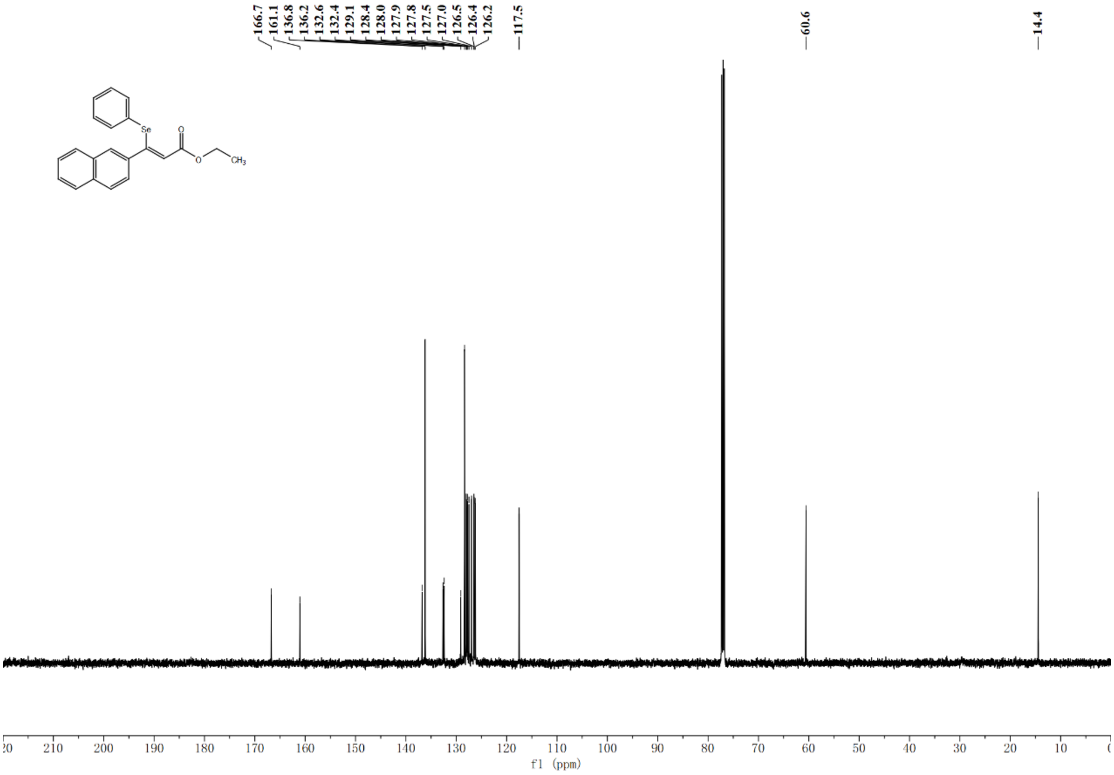

^{1}H NMR (500 MHz) Spectrum of **4maa** in $CDCl_3$

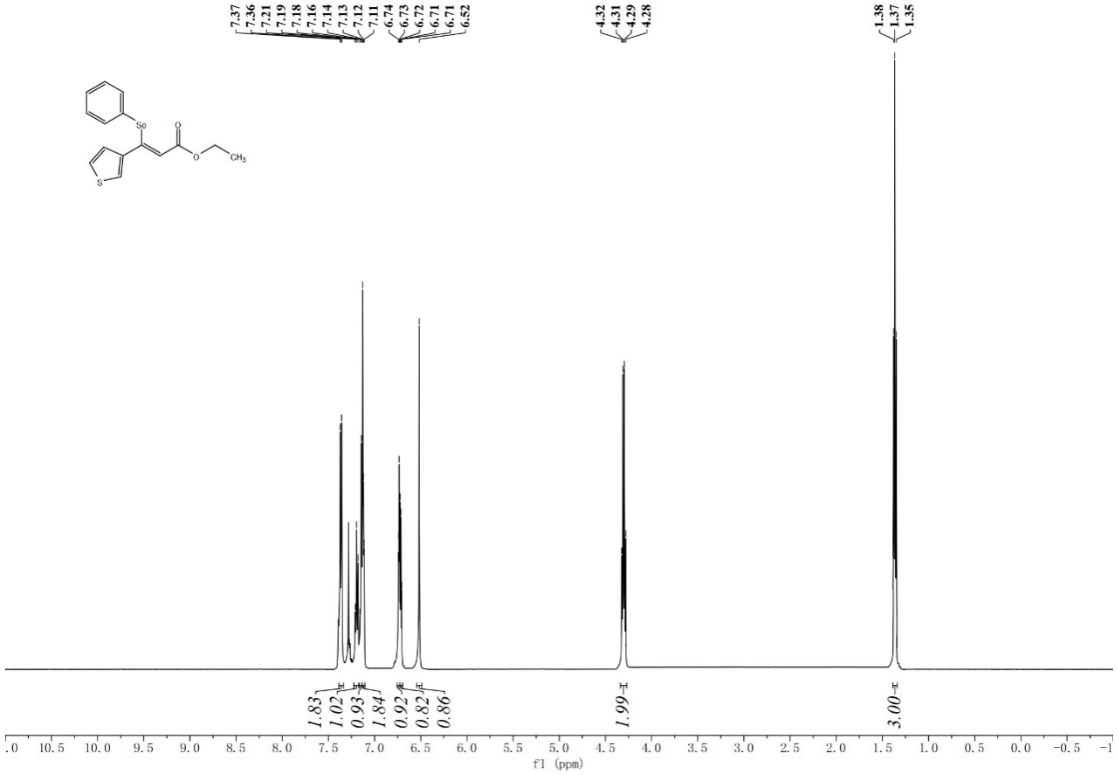

$^{13}C\{^{1}H\}$ NMR (126 MHz) Spectrum of **4maa** in $CDCl_3$

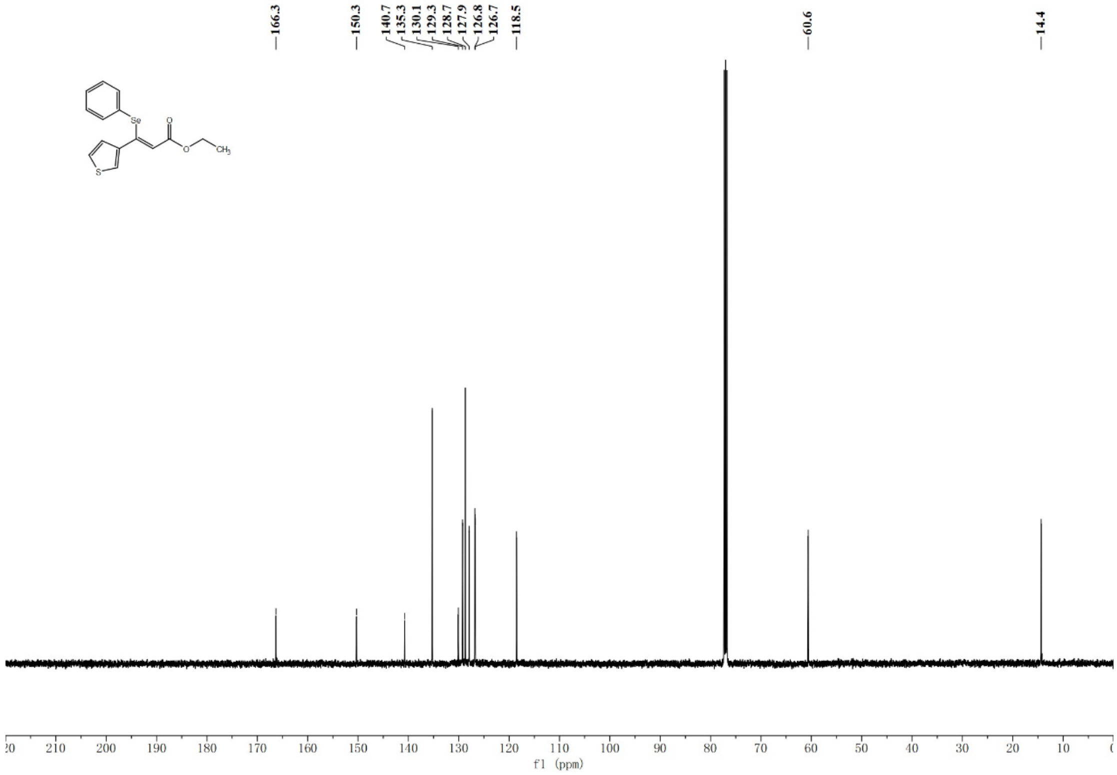

^{1}H NMR (500 MHz) Spectrum of **4naa** in $CDCl_3$

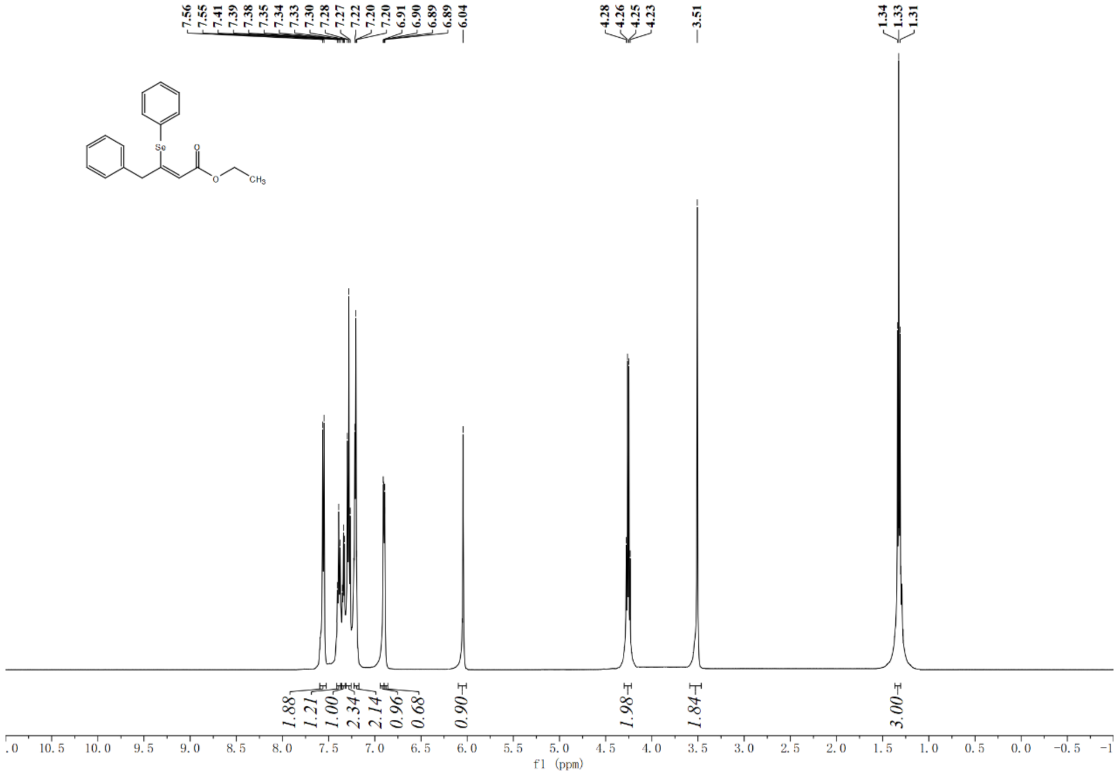

$^{13}C\{^{1}H\}$ NMR (126 MHz) Spectrum of **4naa** in $CDCl_3$

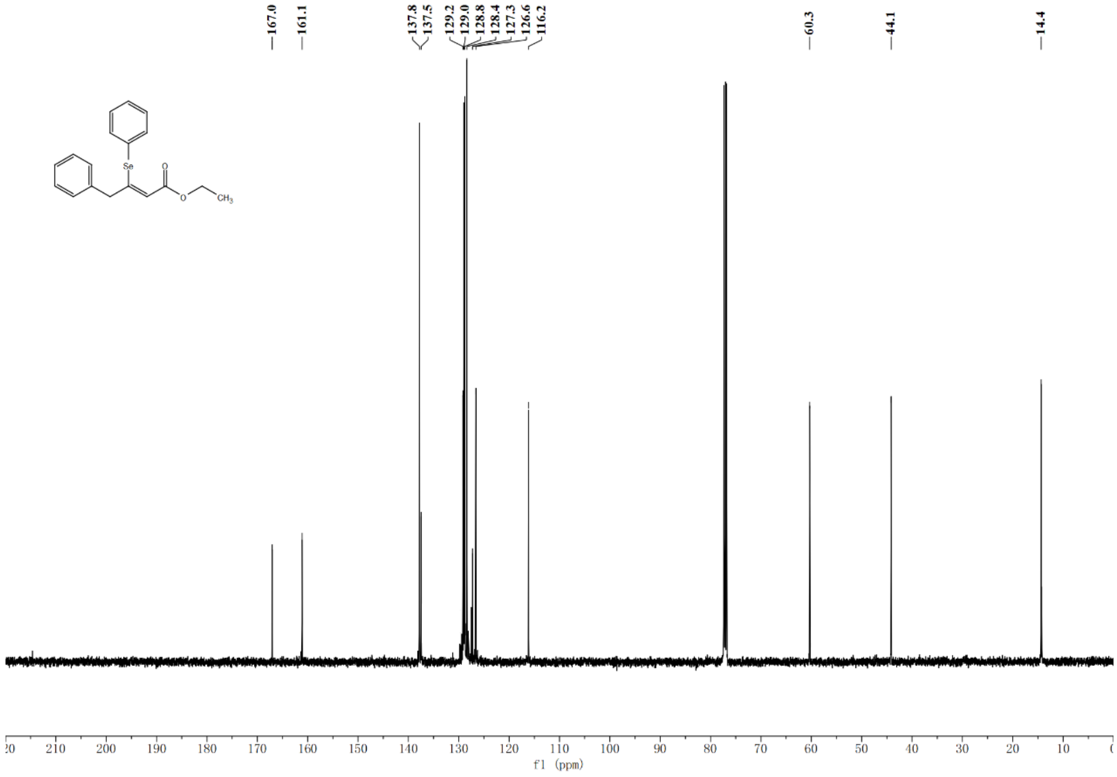

^{1}H NMR (500 MHz) Spectrum of **4oaa** in $CDCl_3$

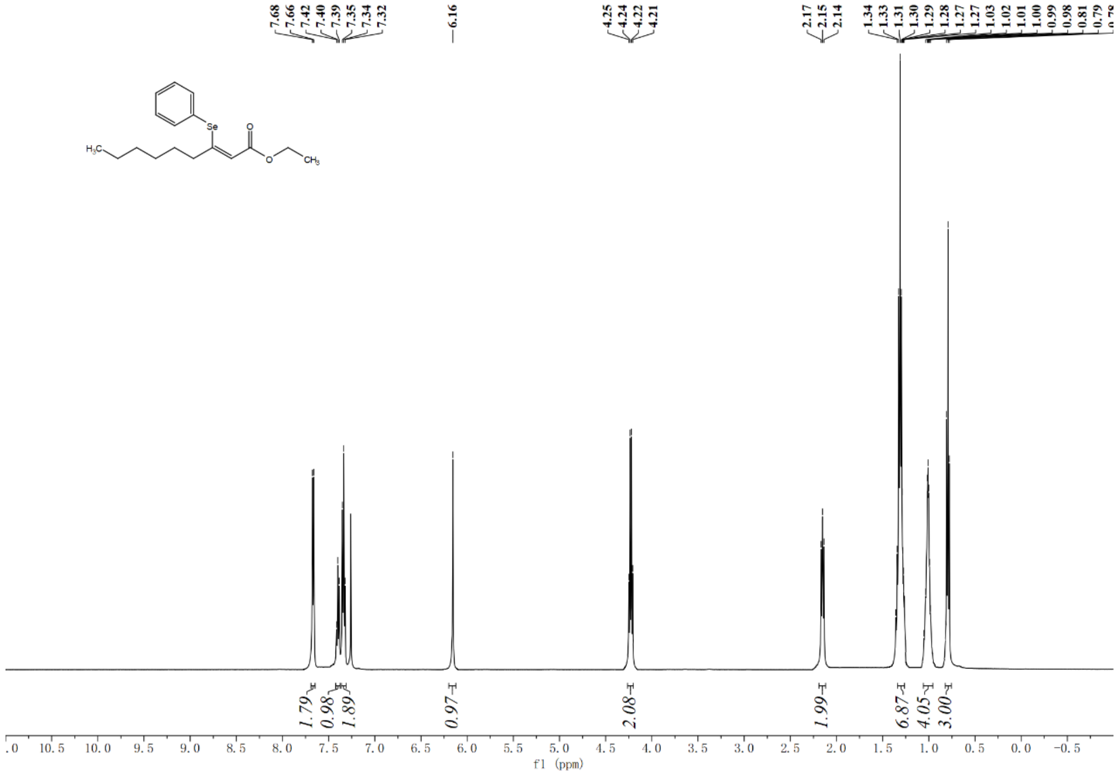

^{13}C{^{1}H} NMR (126 MHz) Spectrum of **4oaa** in $CDCl_3$

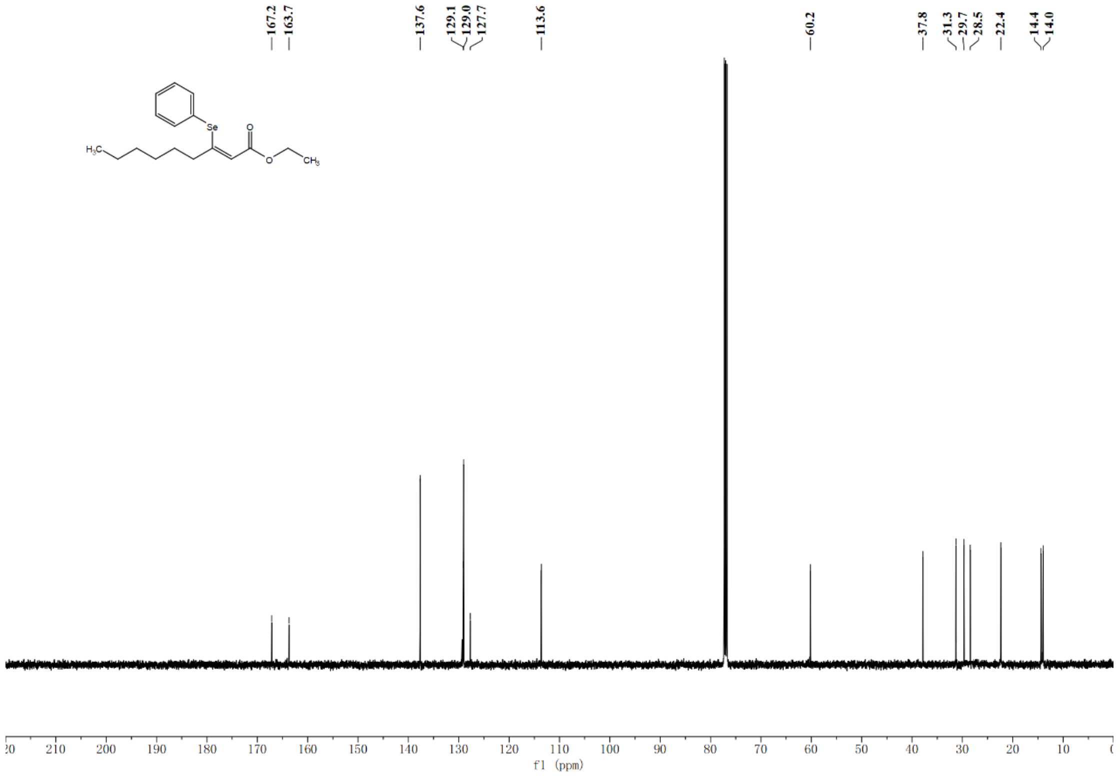

Supplement: Supplementary file 1 [file ol5c02491_si_001.pdf]
